# Supplementary material for: A simple strategy to enhance the speed of protein secondary structure prediction without sacrificing accuracy
Source: PLoS One. 2020 Jun 30;15(6):e0235153. doi: 10.1371/journal.pone.0235153 (PMC7326220; doi:10.1371/journal.pone.0235153)
Supplement: S1 File — (PDF) [file pone.0235153.s001.pdf]

S1 File. Example of PSSMs generated from homologs with high or low sequence homology.

The output of PSI-BLAST shown latter in this file comprises two parts. The left half is the PSSM, and the right half is the position propensity matrix (PPM), *i.e.*, the probability distribution of residue-residue substitutions, of the PSSM.

The first two PSSMs were generated using the same query protein sequence, the amino-terminal domain of the DNA packaging ATPase from bacteriophage phi29 [PDB: 5HD9]. The target dataset for PSSM #1 was composed of protein sequences sharing <90% identities. In this PSSM, most substitution probability values are zero. Among the 3880 PPM entries, the zero probability count up to 3596, or 92.9%. Among the 194 residues of this protein, 85 residues (43.8%) have a substitution probability that concentrates on just one amino acid. The target dataset for PSSM #2 was composed of sequences sharing <25% identities. It contains only 2487, or 64.1%, zero PPM entries, and there are only 3 out of the 194 residues (1.5%) having a substitution probability concentrating on just one amino acid. The following table provides the secondary structure prediction accuracy of several state-of-the-art methods computed using these two PSSMs. No matter measured by Q3/8 or SOV3/8, the PSSM generated from the target dataset of low sequence homology outperforms the other PSSM.

Target dataset	PSSM #1: UniRef90 (identity <90%)				PSSM #2: UniRef25 (identity <25%)			
**Measure**	**Q3**	**SOV3**	**Q8**	**SOV8**	**Q3**	**SOV3**	**Q8**	**SOV8**
**Scorpion**	0.696	0.659	-	-	0.763	0.774	-	-
**SpineX**	0.768	0.776	-	-	0.794	0.785	-	-
**Spider2**	0.722	0.649	-	-	0.799	0.731	-	-
**Psipred3**	0.696	0.644	-	-	0.747	0.604	-	-
**DeepCNF**	0.634	0.634	0.459	0.418	0.820	0.753	0.567	0.538
**RaptorX**	0.619	0.582	0.428	0.402	0.763	0.744	0.510	0.481
**SSpro8+**	0.706	0.678	0.521	0.535	0.799	0.749	0.582	0.574
Average	*0.692*	*0.660*	*0.469*	*0.452*	*0.784*	*0.734*	*0.553*	*0.531*

The next two PSSMs were generated using the myoglobin [PDB: 1MBN]. The sequence homology of the target dataset for PSSM #3 was <90% identities. In this PSSM, 1520 substitution probability values are zero (39.2%). The sequence homology of the target dataset for PSSM #4 was <25% identities. It contained only 498 zero PPM entries (12.8%). These pairs of PSSM were just two out of the numerous examples we observed. See the main text for the hypothesis we raised based on these observations.

PSSM #1

Query protein:
N-terminal domain of the DNA packaging ATPase from bacteriophage phi29 (PDB 5HD9, chain A)

Target dataset:
UniRef90-2015, 3.82 million proteins (randomly sampled)

PSSM generator:
PSI-BLAST

(see the next page)

Last position-specific scoring matrix computed, weighted observed percentages rounded down, information per position, and relative weight of gapless real matches to pseudocounts

		A	R	N	D	C	Q	E	G	H	I	L	K	M	F	P	S	T	W	Y	V	A	R	N	D	C	Q	E	G	H	I	L	K	M	F	P	S	T	W	Y	V		
1	S	1	-1	1	0	-1	0	0	0	-1	-2	-2	0	-2	-2	-1	4	1	-3	-2	-2	0	0	0	0	0	0	0	0	0	0	0	0	0	0	0	0	0	0	0	0	0.00	0.00
2	L	-1	-2	-3	-4	-1	-2	-3	-4	-3	2	4	-2	2	0	-3	-2	-1	-2	-1	1	0	0	0	0	0	0	0	0	0	0	0	0	0	0	0	0	0	0	0	0	0.00	0.00
3	F	-3	-3	-4	-4	-3	-3	-3	-3	-2	-1	0	-3	-1	5	-4	-3	-3	10	3	-2	0	0	0	0	0	0	0	0	0	0	0	0	0	50	0	0	0	50	0	0	1.23	0.00
4	Y	-2	-2	-2	-3	-3	-2	-2	-3	2	-2	-1	-2	-1	3	-3	-2	-2	2	7	-1	0	0	0	0	0	0	0	0	0	0	0	0	0	0	0	0	0	0	100	0	0.87	0.01
5	N	-2	-1	6	1	-3	0	0	0	1	-4	-4	0	-2	-3	-2	1	0	-4	-2	-3	0	0	100	0	0	0	0	0	0	0	0	0	0	0	0	0	0	0	0	0	0.73	0.00
6	P	-1	-2	-2	-2	-3	-1	-1	-2	-2	-3	-3	-1	-3	-4	8	-1	-1	-4	-3	-3	0	0	0	0	0	0	0	0	0	0	0	0	0	0	100	0	0	0	0	0	2.05	0.00
7	Q	-1	1	0	0	-3	6	2	-2	0	-3	-2	1	0	-4	-1	0	-1	-2	-2	-2	0	0	0	0	0	100	0	0	0	0	0	0	0	0	0	0	0	0	0	0	0.66	0.00
8	K	-1	2	0	-1	-3	1	1	-2	-1	-3	-3	5	-2	-3	-1	0	-1	-3	-2	-3	0	0	0	0	0	0	0	0	0	0	0	100	0	0	0	0	0	0	0	0	0.52	0.00
9	X	-1	-1	-1	-1	-1	-1	-1	-1	-1	-1	-1	-1	-1	-1	-1	-1	-1	-1	-1	-1	0	0	0	0	0	0	0	0	0	0	0	0	50	0	0	0	0	0	0	0	0.00	0.00
10	L	-2	-2	-4	-4	-1	-2	-3	-4	-3	2	4	-3	2	0	-3	-3	-1	-2	-1	1	0	0	0	0	0	0	0	0	0	0	100	0	0	0	0	0	0	0	0	0	0.46	0.00
11	S	1	-1	1	0	-1	0	0	0	-1	-3	-3	0	-2	-3	-1	4	2	-3	-2	-2	0	0	0	0	0	0	0	0	0	0	0	0	0	0	0	100	0	0	0	0	0.46	0.00
12	Y	-2	-2	-2	-3	-3	-2	-2	-3	2	-2	-1	-2	-1	3	-3	-2	-2	2	7	-1	0	0	0	0	0	0	0	0	0	0	0	0	0	0	0	0	0	0	100	0	0.87	0.01
13	D	-2	-1	5	5	-3	0	1	-1	0	-4	-4	0	-3	-4	-2	0	-1	-4	-3	-3	0	0	50	50	0	0	0	0	0	0	0	0	0	0	0	0	0	0	0	0	0.67	0.00
14	R	-1	5	0	-1	-4	4	1	-2	0	-3	-2	2	-1	-3	-2	0	-1	-3	-2	-3	0	50	0	0	0	50	0	0	0	0	0	0	0	0	0	0	0	0	0	0	0.57	0.00
15	I	-2	-3	-3	-3	-2	-2	-3	-4	0	3	1	-2	0	2	-3	-2	-1	1	5	1	0	0	0	0	0	0	0	0	0	50	0	0	0	0	0	0	0	0	50	0	0.38	0.00
16	L	-2	-2	-4	-4	-1	-2	-3	-4	-3	2	4	-3	2	0	-3	-3	-1	-2	-1	1	0	0	0	0	0	0	0	0	0	0	100	0	0	0	0	0	0	0	0	0	0.46	0.00
17	N	-2	-1	6	1	-3	0	0	0	1	-4	-4	0	-2	-3	-2	1	0	-4	-2	-3	0	0	100	0	0	0	0	0	0	0	0	0	0	0	0	0	0	0	0	0	0.73	0.00
18	F	-2	-3	-3	-4	-3	-4	-4	-3	-1	0	0	-3	0	7	-4	-3	-2	1	3	-1	0	0	0	0	0	0	0	0	0	0	0	0	0	100	0	0	0	0	0	0	0.74	0.00
19	V	0	-3	-3	-4	-1	-2	-3	-4	-3	3	1	-3	1	-1	-3	-2	0	-3	-1	4	0	0	0	0	0	0	0	0	0	0	0	0	0	0	0	0	0	0	0	100	0.35	0.00
20	I	-1	-3	-4	-3	-1	-3	-4	-4	-4	5	2	-3	1	0	-3	-3	-1	-3	-1	3	0	0	0	0	0	0	0	0	0	100	0	0	0	0	0	0	0	0	0	0	0.39	0.00
21	G	0	-3	0	-1	-3	-2	-2	6	-2	-4	-4	-2	-3	-3	-2	0	-2	-3	-3	-4	0	0	0	0	0	0	0	100	0	0	0	0	0	0	0	0	0	0	0	0	1.04	0.00
22	A	3	-2	-1	-2	-1	-1	-2	4	-2	-3	-3	-1	-2	-3	-2	1	-1	-3	-3	-1	50	0	0	0	0	0	0	50	0	0	0	0	0	0	0	0	0	0	0	0	0.52	0.00
23	R	-2	6	-1	-2	-4	1	0	-3	0	-3	-2	2	-2	-3	-2	-1	-1	-3	-2	-3	0	100	0	0	0	0	0	0	0	0	0	0	0	0	0	0	0	0	0	0	0.75	0.00
24	G	0	-3	0	-1	-3	-2	-2	6	-2	-4	-4	-2	-3	-3	-2	0	-2	-3	-3	-4	0	0	0	0	0	0	0	100	0	0	0	0	0	0	0	0	0	0	0	0	1.04	0.00
25	I	-1	-3	-4	-3	-1	-3	-4	-4	-4	5	2	-3	1	0	-3	-3	-1	-3	-1	3	0	0	0	0	0	0	0	0	0	100	0	0	0	0	0	0	0	0	0	0	0.39	0.00
26	G	0	-3	0	-1	-3	-2	-2	6	-2	-4	-4	-2	-3	-3	-2	0	-2	-3	-3	-4	0	0	0	0	0	0	0	100	0	0	0	0	0	0	0	0	0	0	0	0	1.04	0.00
27	K	-1	2	0	-1	-3	1	1	-2	-1	-3	-3	5	-2	-3	-1	0	-1	-3	-2	-3	0	0	0	0	0	0	0	0	0	0	0	100	0	0	0	0	0	0	0	0	0.52	0.00
28	S	1	-1	0	-1	-1	0	-1	-1	-1	-2	-2	0	-1	-2	-1	3	4	-3	-2	-1	0	0	0	0	0	0	0	0	0	0	0	0	0	0	0	50	50	0	0	0	0.39	0.00
29	Y	-2	-2	-3	-4	-3	-2	-3	-3	1	-1	0	-3	-1	5	-4	-2	-2	2	6	-1	0	0	0	0	0	0	0	0	0	0	0	0	0	50	0	0	0	0	50	0	0.69	0.00
30	A	4	-2	-2	-2	0	-1	-1	0	-2	-1	-2	-1	-1	-2	-1	1	0	-3	-2	0	100	0	0	0	0	0	0	0	0	0	0	0	0	0	0	0	0	0	0	0	0.41	0.00
31	X	-1	-1	-1	-1	-1	-1	-1	-1	-1	-1	-1	-1	-1	-1	-1	-1	-1	-1	-1	-1	0	0	0	0	0	0	0	0	0	0	50	0	0	0	0	0	0	0	0	0	0.00	0.00
32	K	-1	2	0	-1	-3	1	1	-2	-1	-3	-3	5	-2	-3	-1	0	-1	-3	-2	-3	0	0	0	0	0	0	0	0	0	0	0	100	0	0	0	0	0	0	0	0	0.52	0.00
33	V	-1	1	-1	-2	-2	0	0	-2	-2	1	0	3	0	-2	-2	-1	0	-3	-2	2	0	0	0	0	0	0	0	0	0	0	50	0	0	0	0	0	0	0	0	50	0.17	0.00
34	Y	-2	-2	-2	-3	-3	-2	-2	-3	2	-2	-1	-2	-1	3	-3	-2	-2	2	7	-1	0	0	0	0	0	0	0	0	0	0	0	0	0	0	0	0	0	0	100	0	0.87	0.01
35	P	-1	-2	-3	-3	-2	-2	-2	-3	-3	0	2	-2	1	-1	6	-2	-1	-3	-2	0	0	0	0	0	0	0	0	0	0	0	50	0	0	0	50	0	0	0	0	0	0.80	0.00
36	I	-2	-3	-3	-4	-2	-3	-4	-4	-2	3	1	-3	1	5	-4	-3	-1	0	2	1	0	0	0	0	0	0	0	0	0	50	0	0	0	50	0	0	0	0	0	0	0.38	0.00
37	N	-1	1	4	0	-3	1	0	-1	0	-3	-3	3	-2	-3	-2	0	0	-4	-2	-3	0	0	50	0	0	0	0	0	0	0	0	50	0	0	0	0	0	0	0	0	0.45	0.00
38	R	-2	6	-1	-2	-4	1	0	-3	0	-3	-2	2	-2	-3	-2	-1	-1	-3	-2	-3	0	100	0	0	0	0	0	0	0	0	0	0	0	0	0	0	0	0	0	0	0.75	0.00
39	F	-2	-3	-3	-4	-3	-4	-4	-3	-1	0	0	-3	0	7	-4	-3	-2	1	3	-1	0	0	0	0	0	0	0	0	0	0	0	0	0	100	0	0	0	0	0	0	0.74	0.00
40	I	-1	-3	-4	-3	-1	-3	-4	-4	-4	5	2	-3	1	0	-3	-3	-1	-3	-1	3	0	0	0	0	0	0	0	0	0	100	0	0	0	0	0	0	0	0	0	0	0.39	0.00
41	K	-1	1	0	1	-4	2	4	-2	0	-3	-3	4	-2	-3	-1	0	-1	-3	-2	-3	0	0	0	0	0	0	50	0	0	0	0	50	0	0	0	0	0	0	0	0	0.50	0.00
42	Y	-1	1	-1	-2	-3	0	0	-2	1	-2	-2	3	-1	1	-2	-1	-1	1	5	-2	0	0	0	0	0	0	0	0	0	0	0	50	0	0	0	0	0	0	50	0	0.40	0.00
43	G	0	-3	0	-1	-3	-2	-2	6	-2	-4	-4	-2	-3	-3	-2	0	-2	-3	-3	-4	0	0	0	0	0	0	0	100	0	0	0	0	0	0	0	0	0	0	0	0	1.04	0.00
44	E	-1	0	0	2	-4	2	5	-2	0	-4	-3	1	-2	-4	-1	0	-1	-3	-2	-3	0	0	0	0	0	0	100	0	0	0	0	0	0	0	0	0	0	0	0	0	0.74	0.00
45	Q	-1	1	0	0	-3	6	2	-2	0	-3	-2	1	0	-4	-1	0	-1	-2	-2	-2	0	0	0	0	0	100	0	0	0	0	0	0	0	0	0	0	0	0	0	0	0.66	0.00
46	F	-2	-3	-3	-4	-3	-4	-4	-3	-1	0	0	-3	0	7	-4	-3	-2	1	3	-1	0	0	0	0	0	0	0	0	0	0	0	0	0	100	0	0	0	0	0	0	0.74	0.00
47	I	-1	-3	-4	-3	-1	-3	-4	-4	-4	5	2	-3	1	0	-3	-3	-1	-3	-1	3	0	0	0	0	0	0	0	0	0	100	0	0	0	0	0	0	0	0	0	0	0.39	0.00
48	Y	-2	-2	-2	-3	-3	-2	-2	-3	2	-2	-1	-2	-1	3	-3	-2	-2	2	7	-1	0	0	0	0	0	0	0	0	0	0	0	0	0	0	0	0	0	0	100	0	0.87	0.01
49	V	-1	-3	-3	-4	-1	-2	-3	-4	-3	2	3	-3	2	0	-3	-2	-1	-2	-1	3	0	0	0	0	0	0	0	0	0	0	50	0	0	0	0	0	0	0	0	50	0.32	0.00
50	R	-2	6	-1	-2	-4	1	0	-3	0	-3	-2	2	-2	-3	-2	-1	-1	-3	-2	-3	0	100	0	0	0	0	0	0	0	0	0	0	0	0	0	0	0	0	0	0	0.75	0.00
51	R	-2	6	-1	-2	-4	1	0	-3	0	-3	-2	2	-2	-3	-2	-1	-1	-3	-2	-3	0	100	0	0	0	0	0	0	0	0	0	0	0	0	0	0	0	0	0	0	0.75	0.00
52	Y	-2	-1	4	0	-3	-1	-1	-2	1	-2	-2	-1	-2	1	-3	0	-1	1	5	-2	0	0	50	0	0	0	0	0	0	0	0	0	0	0	0	0	0	0	50	0	0.47	0.00
53	K	-1	2	0	-1	-3	1	1	-2	-1	-3	-3	5	-2	-3	-1	0	-1	-3	-2	-3	0	0	0	0	0	0	0	0	0	0	0	100	0	0	0	0	0	0	0	0	0.52	0.00
54	P	0	-2	0	-1	-2	-1	-1	-1	-2	-3	-3	-1	-2	-3	6	3	0	-4	-3	-2	0	0	0	0	0	0	0	0	0	0	0	0	0	0	50	50	0	0	0	0	0.91	0.00
55	E	-1	0	0	2	-4	2	5	-2	0	-4	-3	1	-2	-4	-1	0	-1	-3	-2	-3	0	0	0	0	0	0	100	0	0	0	0	0	0	0	0	0	0	0	0	0	0.74	0.00
56	L	-2	-2	-4	-4	-1	-2	-3	-4	-3	2	4	-3	2	0	-3	-3	-1	-2	-1	1	0	0	0	0	0	0	0	0	0	0	100	0	0	0	0	0	0	0	0	0	0.46	0.00
57	A	3	-2	0	4	-2	-1	1	-1	-2	-2	-3	-1	-2	-3	-1	1	-1	-4	-3	-1	50	0	0	50	0	0	0	0	0	0	0	0	0	0	0	0	0	0	0	0	0.41	0.00
58	K	-1	5	0	-1	-4	1	0	-2	-1	-3	-3	4	-2	-3	-2	-1	-1	-3	-2	-3	0	50	0	0	0	0	0	0	0	0	0	50	0	0	0	0	0	0	0	0	0.56	0.00
59	V	-1	-3	-3	-3	-1	-3	-3	-4	-4	4	1	-3	1	-1	-3	-2	0	-3	-1	4	0	0	0	0	0	0	0	0	0	50	0	0	0	0	0	0	0	0	0	50	0.35	0.00
60	S	0	-1	1	5	-2	0	1	-1	-1	-3	-3	-1	-2	-3	-1	3	0	-4	-3	-3	0	0	0	50	0	0	0	0	0	0	0	0	0	0	0	50	0	0	0	0	0.49	0.00
61	N	-1	1	4	0	-3	1	0	-1	0	-3	-3	3	-2	-3	-2	0	0	-4	-2	-3	0	0	50	0	0	0	0	0	0	0	0	50	0	0	0	0	0	0	0	0	0.45	0.00
62	Y	-2	-2	-3	-4	-3	-2	-3	-3	1	-1	0	-3	-1	5	-4	-2	-2	2	6	-1	0	0	0	0	0	0	0	0	0	0	0	0	0	50	0	0	0	0	50	0	0.69	0.00
63	F	-2	-3	-3	-4	-3	-4	-4	-3	-1	0	0	-3	0	7	-4	-3	-2	1	3	-1	0	0	0	0	0	0	0	0	0	0	0	0	0	100	0	0	0	0	0	0	0.74	0.00
64	N	-1	-1	5	0	-2	0	-1	-1	0	-2	-2	0	-1	-3	-2	1	3	-3	-2	-1	0	0	50	0	0	0	0	0	0	0	0	0	0	0	0	0	50	0	0	0	0.42	0.00
65	D	-1	-2	1	5	-2	-1	1	-2	-2	-2	-2	-1	-2	-3	-1	1	3	-4	-3	-1	0	0	0	50	0	0	0	0	0	0	0	0	0	0	0	0	50	0	0	0	0.46	0.00
66	V	-1	-3	-3	-4	-1	-2	-3	-4	-3	2	3	-3	2	0	-3	-2	-1	-2	-1	3	0	0	0	0	0	0	0	0	0	0	50	0	0	0	0	0	0	0	0	50	0.32	0.00
67	A	3	1	-1	-1	-2	0	0	-1	-1	-2	-2	3	-1	-3	-1	1	0	-3	-2	-1	50	0	0	0	0	0	0	0	0	0	0	50	0	0	0	0	0	0	0	0	0.29	0.00
68	Q	-1	1	0	0	-3	6	2	-2	0	-3	-2	1	0	-4	-1	0	-1	-2	-2	-2	0	0	0	0	0	100	0	0	0	0	0	0	0	0	0	0	0	0	0	0	0.66	0.00
69	E	-1	-1	-1	0	-2	1	3	-3	-1	1	-1	0	0	-2	-2	-1	0	-3	-2	2	0	0	0	0	0	0	50	0	0	0	0	0	0	0	0	0	0	0	0	50	0.24	0.00
70	F	-2	-3	-3	-4	-3	-4	-4	-3	-1	0	0	-3	0	7	-4	-3	-2	1	3	-1	0	0	0	0	0	0	0	0	0	0	0	0	0	100	0	0	0	0	0	0	0.74	0.00
71	P	0	-2	-1	-1	-2	-1	-1	-2	-2	-2	-2	-1	-2	-3	6	1	3	-3	-2	-1	0	0	0	0	0	0	0	0	0	0	0	0	0	0	50	0	50	0	0	0	0.90	0.00
72	D	-2	-1	5	5	-3	0	1	-1	0	-4	-4	0	-3	-4	-2	0	-1	-4	-3	-3	0	0	50	50	0	0	0	0	0	0	0	0	0	0	0	0	0	0	0	0	0.67	0.00
73	H	-2	-1	-1	-2	-3	-1	-1	-3	6	-1	-1	-2	-1	5	-3	-2	-2	0	3	-2	0	0	0	0	0	0	0	0	50	0	0	0	0	50	0	0	0	0	0	0	0.51	0.00
74	E	-1	0	0	2	-4	2	5	-2	0	-4	-3	1	-2	-4	-1	0	-1	-3	-2	-3	0	0	0	0	0	0	100	0	0	0	0	0	0	0	0	0	0	0	0	0	0.74	0.00
75	L	-2	-2	-4	-4	-1	-2	-3	-4	-3	2	4	-3	2	0	-3	-3	-1	-2	-1	1	0	0	0	0	0	0	0	0	0	0	100	0	0	0	0	0	0	0	0	0	0.46	0.00
76	V	0	-3	-3	-4	-1	-2	-3	-4	-3	3	1	-3	1	-1	-3	-2	0	-3	-1	4	0	0	0	0	0	0	0	0	0	0	0	0	0	0	0	0	0	0	0	100	0.35	0.00
77	V	-1	-3	-3	-4	-1	-2	-3	-4	-3	2	3	-3	2	0	-3	-2	-1	-2	-1	3	0	0	0	0	0	0	0	0	0	0	50	0	0	0	0	0	0	0	0	50	0.32	0.00
78	K	0	1	0	-1	-2	1	0	-1	-1	-3	-3	3	-2	-3	-1	3	1	-3	-2	-2	0	0	0	0	0	0	0	0	0	0	0	50	0	0	0	50	0	0	0	0	0.34	0.00
79	G	1	-2	0	-1	-2	-1	-1	4	-2	-3	-3	-1	-2	-3	-2	3	0	-3	-3	-3	0	0	0	0	0	0	0	50	0	0	0	0	0	0	0	50	0	0	0	0	0.53	0.00
80	R	-1	4	0	-1	-2	0	-1	-2	-1	-2	-2	1	-1	-3	-2	1	3	-3	-2	-1	0	50	0	0	0	0	0	0	0	0	0	0	0	0	0	0	50	0	0	0	0.39	0.00
81	R	-1	5	0	-1	-4	1	0	-2	-1	-3	-3	4	-2	-3	-2	-1	-1	-3	-2	-3	0	50	0	0	0	0	0	0	0	0	0	50	0	0	0	0	0	0	0	0	0.56	0.00
82	F	-2	-3	-3	-4	-2	-3	-4	-4	-2	3	1	-3	1	5	-4	-3	-1	0	2	1	0	0	0	0	0	0	0	0	0	50	0	0	0	50	0	0	0	0	0	0	0.38	0.00
83	Y	-2	-3	-3	-3	-2	-2	-3	-4	0	3	1	-2	0	2	-3	-2	-1	1	5	1	0	0	0	0	0	0	0	0	0	50	0	0	0	0	0	0	0	0	50	0	0.38	0.00
84	I	-2	-3	-4	-4	-1	-3	-3	-4	-3	3	3	-3	2	0	-3	-3	-1	-2	-1	2	0	0	0	0	0	0	0	0	0	50	50	0	0	0	0	0	0	0	0	0	0.37	0.00
85	D	-2	-1	5	5	-3	0	1	-1	0	-4	-4	0	-3	-4	-2	0	-1	-4	-3	-3	0	0	50	50	0	0	0	0	0	0	0	0	0	0	0	0	0	0	0	0	0.67	0.00
86	G	0	-3	0	-1	-3	-2	-2	6	-2	-4	-4	-2	-3	-3	-2	0	-2	-3	-3	-4	0	0	0	0	0	0	0	100	0	0	0	0	0	0	0	0	0	0	0	0	1.04	0.00
87	K	-1	2	0	-1	-3	1	1	-2	-1	-3	-3	5	-2	-3	-1	0	-1	-3	-2	-3	0	0	0	0	0	0	0	0	0	0	0	100	0	0	0	0	0	0	0	0	0.52	0.00
88	L	-2	-3	-4	-4	-1	-3	-3	-4	-3	3	3	-3	2	0	-3	-3	-1	-2	-1	2	0	0	0	0	0	0	0	0	0	50	50	0	0	0	0	0	0	0	0	0	0.37	0.00
89	A	3	-2	-2	-3	-1	-2	-2	-1	-3	1	0	-2	0	-2	-2	0	0	-3	-2	3	50	0	0	0	0	0	0	0	0	0	0	0	0	0	0	0	0	0	0	50	0.21	0.00
90	G	0	-3	-1	-2	7	-3	-3	4	-3	-2	-2	-2	-2	-3	-3	-1	-1	-3	-3	-2	0	0	0	0	50	0	0	50	0	0	0	0	0	0	0	0	0	0	0	0	0.75	0.00
91	W	-2	-2	-3	-4	-3	-2	-3	-3	0	-2	-2	-3	-1	2	-4	-2	-2	10	6	-2	0	0	0	0	0	0	0	0	0	0	0	0	0	0	0	0	0	50	50	0	1.31	0.00
92	A	3	-2	-3	-3	-1	-2	-2	-1	-2	0	3	-2	1	-1	-2	0	-1	-2	-2	0	50	0	0	0	0	0	0	0	0	0	50	0	0	0	0	0	0	0	0	0	0.20	0.00
93	I	-1	1	-1	-2	-2	0	-1	-3	-2	3	0	3	0	-1	-2	-1	-1	-3	-2	1	0	0	0	0	0	0	0	0	0	50	0	50	0	0	0	0	0	0	0	0	0.16	0.00
94	P	0	-2	0	-1	-2	-1	-1	-1	-2	-3	-3	-1	-2	-3	6	3	0	-4	-3	-2	0	0	0	0	0	0	0	0	0	0	0	0	0	0	50	50	0	0	0	0	0.91	0.00
95	L	-2	-2	-4	-4	-1	-2	-3	-4	-3	2	4	-3	2	0	-3	-3	-1	-2	-1	1	0	0	0	0	0	0	0	0	0	0	100	0	0	0	0	0	0	0	0	0	0.46	0.00
96	S	1	-1	1	0	-1	0	0	0	-1	-3	-3	0	-2	-3	-1	4	2	-3	-2	-2	0	0	0	0	0	0	0	0	0	0	0	0	0	0	0	100	0	0	0	0	0.46	0.00
97	V	0	-2	-1	-2	-1	-1	-2	-3	-3	1	0	-2	0	-2	-2	0	3	-3	-2	3	0	0	0	0	0	0	0	0	0	0	0	0	0	0	0	0	50	0	0	50	0.25	0.00
98	W	-3	-3	-4	-5	-3	-2	-3	-3	-3	-3	-2	-3	-2	1	-4	-3	-3	12	2	-3	0	0	0	0	0	0	0	0	0	0	0	0	0	0	0	0	0	100	0	0	2.33	0.01
99	Q	-1	0	-1	-2	-2	4	0	-3	-1	1	0	0	0	-2	-2	-1	0	-3	-1	2	0	0	0	0	0	50	0	0	0	0	0	0	0	0	0	0	0	0	0	50	0.23	0.00
100	S	0	-1	1	5	-2	0	1	-1	-1	-3	-3	-1	-2	-3	-1	3	0	-4	-3	-3	0	0	0	50	0	0	0	0	0	0	0	0	0	0	0	50	0	0	0	0	0.49	0.00
101	E	-1	-1	-2	0	-2	1	3	-3	-1	0	2	0	1	-1	-2	-1	-1	-2	-2	0	0	0	0	0	0	0	50	0	0	0	50	0	0	0	0	0	0	0	0	0	0.26	0.00
102	K	-1	2	0	-1	-3	1	1	-2	-1	-3	-3	5	-2	-3	-1	0	-1	-3	-2	-3	0	0	0	0	0	0	0	0	0	0	0	100	0	0	0	0	0	0	0	0	0.52	0.00
103	S	1	-2	0	-1	-2	-1	-1	4	-2	-3	-3	-1	-2	-3	-2	3	0	-3	-3	-3	0	0	0	0	0	0	0	50	0	0	0	0	0	0	0	50	0	0	0	0	0.53	0.00
104	N	0	-1	5	1	-2	0	0	0	0	-3	-3	0	-2	-3	-2	3	1	-4	-2	-2	0	0	50	0	0	0	0	0	0	0	0	0	0	0	0	50	0	0	0	0	0.45	0.00
105	A	3	-1	-1	0	-2	1	4	-1	-1	-2	-2	0	-2	-3	-1	1	0	-3	-2	-1	50	0	0	0	0	0	50	0	0	0	0	0	0	0	0	0	0	0	0	0	0.36	0.00
106	Y	-2	-2	-2	-3	-3	-2	-2	-3	2	-2	-1	-2	-1	3	-3	-2	-2	2	7	-1	0	0	0	0	0	0	0	0	0	0	0	0	0	0	0	0	0	0	100	0	0.87	0.01
107	P	-1	-2	0	4	-3	-1	1	-2	-2	-3	-4	-1	-3	-4	6	-1	-1	-4	-3	-3	0	0	0	50	0	0	0	0	0	0	0	0	0	0	50	0	0	0	0	0	1.07	0.00
108	N	-1	0	4	2	-3	1	4	-1	0	-4	-3	0	-2	-3	-2	0	0	-4	-2	-3	0	0	50	0	0	0	50	0	0	0	0	0	0	0	0	0	0	0	0	0	0.54	0.00
109	V	0	-3	-3	-4	-1	-2	-3	-4	-3	3	1	-3	1	-1	-3	-2	0	-3	-1	4	0	0	0	0	0	0	0	0	0	0	0	0	0	0	0	0	0	0	0	100	0.35	0.00
110	S	0	-1	-1	-1	-1	0	-1	-1	-1	0	1	-1	4	-1	-2	3	1	-2	-1	0	0	0	0	0	0	0	0	0	0	0	0	0	50	0	0	50	0	0	0	0	0.19	0.00
111	T	1	-1	0	-1	-1	0	-1	-1	-1	-2	-2	0	-1	-2	-1	3	4	-3	-2	-1	0	0	0	0	0	0	0	0	0	0	0	0	0	0	0	50	50	0	0	0	0.39	0.00
112	I	-1	-3	-4	-3	-1	-3	-4	-4	-4	5	2	-3	1	0	-3	-3	-1	-3	-1	3	0	0	0	0	0	0	0	0	0	100	0	0	0	0	0	0	0	0	0	0	0.39	0.00
113	V	-1	-3	-3	-4	-1	-2	-3	-4	-3	2	3	-3	2	0	-3	-2	-1	-2	-1	3	0	0	0	0	0	0	0	0	0	0	50	0	0	0	0	0	0	0	0	50	0.32	0.00
114	F	-2	-2	-3	-4	-3	-2	-3	-3	1	-1	0	-3	-1	5	-4	-2	-2	2	6	-1	0	0	0	0	0	0	0	0	0	0	0	0	0	50	0	0	0	0	50	0	0.69	0.00
115	D	-2	-2	1	6	-4	0	2	-1	-1	-3	-4	-1	-3	-4	-2	0	-1	-5	-3	-4	0	0	0	100	0	0	0	0	0	0	0	0	0	0	0	0	0	0	0	0	0.93	0.00
116	E	-1	0	0	2	-4	2	5	-2	0	-4	-3	1	-2	-4	-1	0	-1	-3	-2	-3	0	0	0	0	0	0	100	0	0	0	0	0	0	0	0	0	0	0	0	0	0.74	0.00
117	F	-2	-3	-4	-4	-2	-3	-3	-4	-2	1	3	-3	1	5	-4	-3	-2	0	2	0	0	0	0	0	0	0	0	0	0	0	50	0	0	50	0	0	0	0	0	0	0.43	0.00
118	I	-1	-3	-4	-3	-1	-3	-4	-4	-4	5	2	-3	1	0	-3	-3	-1	-3	-1	3	0	0	0	0	0	0	0	0	0	100	0	0	0	0	0	0	0	0	0	0	0.39	0.00

119	R	-2	4	1	4	-4	0	1	-2	-1	-3	-3	1	-2	-3	-2	-1	-1	-4	-3	-3	0	50	0	50	0	0	0	0	0	0	0	0	0	0	0	0	0	0	0	0	0.56	0.00
120	E	-1	-1	-1	0	-2	1	3	-3	-1	1	-1	0	0	-2	-2	-1	0	-3	-2	2	0	0	0	0	0	0	50	0	0	0	0	0	0	0	0	0	0	0	0	50	0.24	0.00
121	K	0	1	0	-1	-2	0	0	-2	-1	-2	-2	3	-1	-3	-1	1	3	-3	-2	-1	0	0	0	0	0	0	0	0	0	0	0	50	0	0	0	50	0	0	0	0	0.32	0.00
122	D	0	-1	1	5	-2	0	1	-1	-1	-3	-3	-1	-2	-3	-1	3	0	-4	-3	-3	0	0	0	50	0	0	0	0	0	0	0	0	0	0	0	50	0	0	0	0	0.49	0.00
123	N	-1	1	4	0	-3	1	0	-1	0	-3	-3	3	-2	-3	-2	0	0	-4	-2	-3	0	0	50	0	0	0	0	0	0	0	0	50	0	0	0	0	0	0	0	0	0.45	0.00
124	S	0	1	0	-1	-2	1	0	-1	-1	-3	-3	3	-2	-3	-1	3	1	-3	-2	-2	0	0	0	0	0	0	0	0	0	0	0	50	0	0	0	50	0	0	0	0	0.34	0.00
125	N	-2	4	4	0	-3	1	0	-1	0	-3	-3	1	-2	-3	-2	0	-1	-4	-2	-3	0	50	50	0	0	0	0	0	0	0	0	0	0	0	0	0	0	0	0	0	0.52	0.00
126	Y	-2	-2	-2	-3	-3	-2	-2	-3	2	-2	-1	-2	-1	3	-3	-2	-2	2	7	-1	0	0	0	0	0	0	0	0	0	0	0	0	0	0	0	0	0	0	100	0	0.87	0.01
127	I	-2	-3	-4	-4	-1	-3	-3	-4	-3	3	3	-3	2	0	-3	-3	-1	-2	-1	2	0	0	0	0	0	0	0	0	0	50	50	0	0	0	0	0	0	0	0	0	0.37	0.00
128	P	-1	-2	0	4	-3	-1	1	-2	-2	-3	-4	-1	-3	-4	6	-1	-1	-4	-3	-3	0	0	0	50	0	0	0	0	0	0	0	0	0	0	50	0	0	0	0	0	1.07	0.00
129	N	-2	-1	6	1	-3	0	0	0	1	-4	-4	0	-2	-3	-2	1	0	-4	-2	-3	0	0	100	0	0	0	0	0	0	0	0	0	0	0	0	0	0	0	0	0	0.73	0.00
130	E	-1	0	0	2	-4	2	5	-2	0	-4	-3	1	-2	-4	-1	0	-1	-3	-2	-3	0	0	0	0	0	0	100	0	0	0	0	0	0	0	0	0	0	0	0	0	0.74	0.00
131	V	0	-3	-3	-4	-1	-2	-3	-4	-3	3	1	-3	1	-1	-3	-2	0	-3	-1	4	0	0	0	0	0	0	0	0	0	0	0	0	0	0	0	0	0	0	0	100	0.35	0.00
132	S	0	0	0	1	-2	1	4	-1	-1	-3	-3	0	-2	-3	-1	3	1	-3	-2	-2	0	0	0	0	0	0	50	0	0	0	0	0	0	0	0	50	0	0	0	0	0.43	0.00
133	A	4	-2	-2	-2	0	-1	-1	0	-2	-1	-2	-1	-1	-2	-1	1	0	-3	-2	0	100	0	0	0	0	0	0	0	0	0	0	0	0	0	0	0	0	0	0	0	0.41	0.00
134	L	-2	-2	-4	-4	-1	-2	-3	-4	-3	2	4	-3	2	0	-3	-3	-1	-2	-1	1	0	0	0	0	0	0	0	0	0	0	100	0	0	0	0	0	0	0	0	0	0.46	0.00
135	L	-2	-2	-4	-4	-1	-2	-3	-4	-3	2	4	-3	2	0	-3	-3	-1	-2	-1	1	0	0	0	0	0	0	0	0	0	0	100	0	0	0	0	0	0	0	0	0	0.46	0.00
136	N	-2	-1	6	1	-3	0	0	0	1	-4	-4	0	-2	-3	-2	1	0	-4	-2	-3	0	0	100	0	0	0	0	0	0	0	0	0	0	0	0	0	0	0	0	0	0.73	0.00
137	L	-2	-3	-4	-4	-2	-3	-3	-4	-2	1	3	-3	1	5	-4	-3	-2	0	2	0	0	0	0	0	0	0	0	0	0	0	50	0	0	50	0	0	0	0	0	0	0.43	0.00
138	X	-1	-1	-1	-1	-1	-1	-1	-1	-1	-1	-1	-1	-1	-1	-1	-1	-1	-1	-1	-1	0	0	0	0	0	0	0	0	0	50	0	0	0	0	0	0	0	0	0	0	0.00	0.00
139	D	-2	-2	0	4	-3	-2	0	-2	-1	-1	-1	-2	-1	5	-3	-1	-2	-1	1	-2	0	0	0	50	0	0	0	0	0	0	0	0	0	50	0	0	0	0	0	0	0.43	0.00
140	T	1	-1	0	-1	-1	0	-1	-1	-1	-2	-2	0	-1	-2	-1	3	4	-3	-2	-1	0	0	0	0	0	0	0	0	0	0	0	0	0	0	0	50	50	0	0	0	0.39	0.00
141	V	0	-3	-3	-4	-1	-2	-3	-4	-3	3	1	-3	1	-1	-3	-2	0	-3	-1	4	0	0	0	0	0	0	0	0	0	0	0	0	0	0	0	0	0	0	0	100	0.35	0.00
142	F	-2	-3	-3	-4	-3	-4	-4	-3	-1	0	0	-3	0	7	-4	-3	-2	1	3	-1	0	0	0	0	0	0	0	0	0	0	0	0	0	100	0	0	0	0	0	0	0.74	0.00
143	R	-2	6	-1	-2	-4	1	0	-3	0	-3	-2	2	-2	-3	-2	-1	-1	-3	-2	-3	0	100	0	0	0	0	0	0	0	0	0	0	0	0	0	0	0	0	0	0	0.75	0.00
144	N	-2	4	4	0	-3	1	0	-1	0	-3	-3	1	-2	-3	-2	0	-1	-4	-2	-3	0	50	50	0	0	0	0	0	0	0	0	0	0	0	0	0	0	0	0	0	0.52	0.00
145	R	-2	6	-1	-2	-4	1	0	-3	0	-3	-2	2	-2	-3	-2	-1	-1	-3	-2	-3	0	100	0	0	0	0	0	0	0	0	0	0	0	0	0	0	0	0	0	0	0.75	0.00
146	E	-1	-1	1	5	-4	1	4	-2	-1	-4	-4	0	-3	-4	-1	0	-1	-4	-3	-3	0	0	0	50	0	0	50	0	0	0	0	0	0	0	0	0	0	0	0	0	0.69	0.00
147	R	-1	4	0	-2	-3	0	-1	4	-1	-4	-3	1	-2	-3	-2	-1	-1	-3	-3	-3	0	50	0	0	0	0	0	50	0	0	0	0	0	0	0	0	0	0	0	0	0.56	0.00
148	V	0	-3	-3	-4	7	-3	-3	-3	-3	1	0	-3	0	-2	-3	-1	0	-3	-2	3	0	0	0	0	50	0	0	0	0	0	0	0	0	0	0	0	0	0	0	50	0.54	0.00
149	R	-2	4	0	-2	-4	1	0	-2	6	-3	-3	1	-2	-2	-2	-1	-2	-3	1	-3	0	50	0	0	0	0	0	0	50	0	0	0	0	0	0	0	0	0	0	0	0.58	0.00
150	C	3	-3	-2	-3	7	-2	-2	-1	-2	-1	-2	-2	-1	-3	-2	0	0	-3	-2	-1	50	0	0	0	50	0	0	0	0	0	0	0	0	0	0	0	0	0	0	0	0.56	0.00
151	I	-2	-3	-3	-3	-2	-2	-3	-4	0	3	1	-2	0	2	-3	-2	-1	1	5	1	0	0	0	0	0	0	0	0	0	50	0	0	0	0	0	0	0	0	50	0	0.38	0.00
152	C	-1	-3	-3	-4	7	-3	-4	-3	-3	0	3	-3	1	-1	-3	-2	-1	-2	-2	0	0	0	0	0	50	0	0	0	0	0	50	0	0	0	0	0	0	0	0	0	0.56	0.00
153	L	-2	-2	-4	-4	-1	-2	-3	-4	-3	2	4	-3	2	0	-3	-3	-1	-2	-1	1	0	0	0	0	0	0	0	0	0	0	100	0	0	0	0	0	0	0	0	0	0.46	0.00
154	S	1	-1	1	0	-1	0	0	0	-1	-3	-3	0	-2	-3	-1	4	2	-3	-2	-2	0	0	0	0	0	0	0	0	0	0	0	0	0	0	0	100	0	0	0	0	0.46	0.00
155	N	-2	-1	6	1	-3	0	0	0	1	-4	-4	0	-2	-3	-2	1	0	-4	-2	-3	0	0	100	0	0	0	0	0	0	0	0	0	0	0	0	0	0	0	0	0	0.73	0.00
156	A	4	-2	-2	-2	0	-1	-1	0	-2	-1	-2	-1	-1	-2	-1	1	0	-3	-2	0	100	0	0	0	0	0	0	0	0	0	0	0	0	0	0	0	0	0	0	0	0.41	0.00
157	V	1	-2	-1	-2	-1	-1	-1	-2	-2	1	0	-1	0	-2	-2	3	1	-3	-2	2	0	0	0	0	0	0	0	0	0	0	0	0	0	0	0	50	0	0	0	50	0.18	0.00
158	S	0	-1	5	1	-2	0	0	0	0	-3	-3	0	-2	-3	-2	3	1	-4	-2	-2	0	0	50	0	0	0	0	0	0	0	0	0	0	0	0	50	0	0	0	0	0.45	0.00
159	V	-1	-3	-3	-4	-2	-3	-3	-3	-2	2	1	-3	0	5	-3	-2	-1	0	2	3	0	0	0	0	0	0	0	0	0	0	0	0	0	50	0	0	0	0	0	50	0.35	0.00
160	V	-1	-1	4	0	-2	-1	-1	-2	-1	1	-1	-1	0	-2	-2	0	0	-4	-2	2	0	0	50	0	0	0	0	0	0	0	0	0	0	0	0	0	0	0	0	50	0.23	0.00
161	N	-2	-1	6	1	-3	0	0	0	1	-4	-4	0	-2	-3	-2	1	0	-4	-2	-3	0	0	100	0	0	0	0	0	0	0	0	0	0	0	0	0	0	0	0	0	0.73	0.00
162	P	-1	-2	-2	-2	-3	-1	-1	-2	-2	-3	-3	-1	-3	-4	8	-1	-1	-4	-3	-3	0	0	0	0	0	0	0	0	0	0	0	0	0	0	100	0	0	0	0	0	2.05	0.00
163	Y	-2	-2	-2	-3	-3	-2	-2	-3	2	-2	-1	-2	-1	3	-3	-2	-2	2	7	-1	0	0	0	0	0	0	0	0	0	0	0	0	0	0	0	0	0	0	100	0	0.87	0.01
164	F	-2	-3	-3	-4	-3	-4	-4	-3	-1	0	0	-3	0	7	-4	-3	-2	1	3	-1	0	0	0	0	0	0	0	0	0	0	0	0	0	100	0	0	0	0	0	0	0.74	0.00
165	L	3	-2	-3	-3	-1	-2	-2	-1	-2	0	3	-2	1	-1	-2	0	-1	-2	-2	0	50	0	0	0	0	0	0	0	0	0	50	0	0	0	0	0	0	0	0	0	0.20	0.00
166	F	-2	-3	-3	-4	-3	-4	-4	-3	-1	0	0	-3	0	7	-4	-3	-2	1	3	-1	0	0	0	0	0	0	0	0	0	0	0	0	0	100	0	0	0	0	0	0	0.74	0.00
167	F	-2	-3	-4	-4	-2	-3	-3	-4	-2	1	3	-3	1	5	-4	-3	-2	0	2	0	0	0	0	0	0	0	0	0	0	0	50	0	0	50	0	0	0	0	0	0	0.43	0.00
168	N	-1	1	4	0	-3	1	0	-1	0	-3	-3	3	-2	-3	-2	0	0	-4	-2	-3	0	0	50	0	0	0	0	0	0	0	0	50	0	0	0	0	0	0	0	0	0.45	0.00
169	L	-2	-3	-4	-4	-2	-3	-3	-4	-2	1	3	-3	1	5	-4	-3	-2	0	2	0	0	0	0	0	0	0	0	0	0	0	50	0	0	50	0	0	0	0	0	0	0.43	0.00
170	V	-1	-2	-3	-3	-2	-2	-2	-3	0	1	0	-2	0	2	-3	-2	-1	1	5	3	0	0	0	0	0	0	0	0	0	0	0	0	0	0	0	0	0	0	50	50	0.37	0.00
171	P	-1	-2	0	4	-3	-1	1	-2	-2	-3	-4	-1	-3	-4	6	-1	-1	-4	-3	-3	0	0	0	50	0	0	0	0	0	0	0	0	0	0	50	0	0	0	0	0	1.07	0.00
172	D	-2	-2	1	6	-4	0	2	-1	-1	-3	-4	-1	-3	-4	-2	0	-1	-5	-3	-4	0	0	0	100	0	0	0	0	0	0	0	0	0	0	0	0	0	0	0	0	0.93	0.00
173	V	-1	-1	4	0	-2	-1	-1	-2	-1	1	-1	-1	0	-2	-2	0	0	-4	-2	2	0	0	50	0	0	0	0	0	0	0	0	0	0	0	0	0	0	0	0	50	0.23	0.00
174	N	-1	-1	4	0	-3	-1	-1	4	-1	-4	-4	-1	-3	-3	-2	0	-1	-3	-3	-3	0	0	50	0	0	0	0	50	0	0	0	0	0	0	0	0	0	0	0	0	0.62	0.00
175	K	-1	2	0	-1	-3	1	1	-2	-1	-3	-3	5	-2	-3	-1	0	-1	-3	-2	-3	0	0	0	0	0	0	0	0	0	0	0	100	0	0	0	0	0	0	0	0	0.52	0.00
176	R	-2	6	-1	-2	-4	1	0	-3	0	-3	-2	2	-2	-3	-2	-1	-1	-3	-2	-3	0	100	0	0	0	0	0	0	0	0	0	0	0	0	0	0	0	0	0	0	0.75	0.00
177	F	-2	-3	-3	-4	-3	-4	-4	-3	-1	0	0	-3	0	7	-4	-3	-2	1	3	-1	0	0	0	0	0	0	0	0	0	0	0	0	0	100	0	0	0	0	0	0	0.74	0.00
178	N	-2	-1	6	1	-3	0	0	0	1	-4	-4	0	-2	-3	-2	1	0	-4	-2	-3	0	0	100	0	0	0	0	0	0	0	0	0	0	0	0	0	0	0	0	0	0.73	0.00
179	V	-1	-3	-3	-4	-1	-2	-3	-4	-3	2	3	-3	2	0	-3	-2	-1	-2	-1	3	0	0	0	0	0	0	0	0	0	0	50	0	0	0	0	0	0	0	0	50	0.32	0.00
180	Y	-1	1	-1	-2	-3	0	0	-2	1	-2	-2	3	-1	1	-2	-1	-1	1	5	-2	0	0	0	0	0	0	0	0	0	0	0	50	0	0	0	0	0	0	50	0	0.40	0.00
181	D	-1	0	1	5	-4	4	2	-2	0	-3	-3	0	-2	-4	-2	0	-1	-3	-2	-3	0	0	0	50	0	50	0	0	0	0	0	0	0	0	0	0	0	0	0	0	0.58	0.00
182	D	-2	-2	0	4	-3	-1	0	-2	1	-2	-2	-1	-2	1	-2	-1	-1	0	5	-2	0	0	0	50	0	0	0	0	0	0	0	0	0	0	0	0	0	0	50	0	0.52	0.00
183	A	4	-2	-2	-2	0	-1	-1	0	-2	-1	-2	-1	-1	-2	-1	1	0	-3	-2	0	100	0	0	0	0	0	0	0	0	0	0	0	0	0	0	0	0	0	0	0	0.41	0.00
184	L	-2	-2	-4	-4	-1	-2	-3	-4	-3	2	4	-3	2	0	-3	-3	-1	-2	-1	1	0	0	0	0	0	0	0	0	0	0	100	0	0	0	0	0	0	0	0	0	0.46	0.00
185	I	-1	-3	-4	-3	-1	-3	-4	-4	-4	5	2	-3	1	0	-3	-3	-1	-3	-1	3	0	0	0	0	0	0	0	0	0	100	0	0	0	0	0	0	0	0	0	0	0.39	0.00
186	E	-1	0	0	2	-4	2	5	-2	0	-4	-3	1	-2	-4	-1	0	-1	-3	-2	-3	0	0	0	0	0	0	100	0	0	0	0	0	0	0	0	0	0	0	0	0	0.74	0.00
187	I	-2	-3	-3	-4	-2	-3	-4	-4	-2	3	1	-3	1	5	-4	-3	-1	0	2	1	0	0	0	0	0	0	0	0	0	50	0	0	0	50	0	0	0	0	0	0	0.38	0.00
188	P	-1	-2	-2	-2	-3	-1	-1	-2	-2	-3	-3	-1	-3	-4	8	-1	-1	-4	-3	-3	0	0	0	0	0	0	0	0	0	0	0	0	0	0	100	0	0	0	0	0	2.05	0.00
189	D	-2	-2	1	6	-4	0	2	-1	-1	-3	-4	-1	-3	-4	-2	0	-1	-4	-3	-3	0	0	0	0	0	0	0	0	0	0	0	0	0	0	0	0	0	0	0	0	0.00	0.00
190	S	1	-1	1	0	-1	0	0	0	-1	-2	-2	0	-2	-2	-1	4	1	-3	-2	-2	0	0	0	0	0	0	0	0	0	0	0	0	0	0	0	0	0	0	0	0	0.00	0.00
191	L	-1	-2	-3	-4	-1	-2	-3	-4	-3	2	4	-2	2	0	-3	-2	-1	-2	-1	1	0	0	0	0	0	0	0	0	0	0	0	0	0	0	0	0	0	0	0	0	0.00	0.00
192	D	-2	-2	1	6	-4	0	2	-1	-1	-3	-4	-1	-3	-4	-2	0	-1	-4	-3	-3	0	0	0	0	0	0	0	0	0	0	0	0	0	0	0	0	0	0	0	0	0.00	0.00
193	F	-2	-3	-3	-4	-2	-3	-3	-3	-1	0	0	-3	0	6	-4	-2	-2	1	3	-1	0	0	0	0	0	0	0	0	0	0	0	0	0	0	0	0	0	0	0	0	0.00	0.00
194	S	1	-1	1	0	-1	0	0	0	-1	-2	-2	0	-2	-2	-1	4	1	-3	-2	-2	0	0	0	0	0	0	0	0	0	0	0	0	0	0	0	0	0	0	0	0	0.00	0.00

	K	Lambda
Standard Ungapped	0.1414	0.3234
Standard Gapped	0.0410	0.2670
PSI Ungapped	0.1414	0.3234
PSI Gapped	0.0410	0.2670

PSSM #2

Query protein:
N-terminal domain of the DNA packaging ATPase from bacteriophage phi29 (PDB 5HD9, chain A)

Target dataset:
UniRef25-2015, 3.82 million proteins (randomly sampled)

PSSM generator:
PSI-BLAST

(see the next page)

Last position-specific scoring matrix computed, weighted observed percentages rounded down, information per position, and relative weight of gapless real matches to pseudocounts

		A	R	N	D	C	Q	E	G	H	I	L	K	M	F	P	S	T	W	Y	V	A	R	N	D	C	Q	E	G	H	I	L	K	M	F	P	S	T	W	Y	V		
1	S	0	-1	2	2	-2	0	0	-1	-1	-3	-3	-1	-2	-3	-1	4	1	-4	-2	-2	0	0	14	14	0	0	0	0	0	0	0	0	0	0	0	73	0	0	0	0	0.49	0.07
2	L	-1	-2	-2	-3	-2	-1	-2	-3	-3	2	1	0	3	-1	3	-1	1	-3	-2	1	0	0	0	0	0	0	0	0	0	22	14	10	19	0	15	0	12	0	0	9	0.23	0.05
3	F	-3	-3	-4	-5	-3	-3	-4	-4	-1	-2	-1	-4	-1	6	-4	-3	-3	6	6	-2	0	0	0	0	0	0	0	0	0	0	0	0	0	45	0	0	0	14	42	0	1.20	0.19
4	Y	-3	-2	-3	-4	-3	1	-3	-4	0	1	0	-3	-1	4	-4	-3	-2	1	6	-1	0	0	0	0	0	9	0	0	0	11	7	0	0	25	0	0	0	0	48	0	0.71	0.15
5	N	-2	-2	3	5	-3	0	2	-2	-1	-3	-3	-1	1	-3	-2	1	-1	-4	-3	-3	0	0	23	39	0	0	17	0	0	0	0	0	9	0	0	12	0	0	0	0	0.56	0.12
6	P	-2	-3	-3	-3	-2	-3	-3	0	-3	2	1	-3	2	4	1	-2	-2	-2	0	2	0	0	0	0	0	0	0	8	0	23	14	0	6	26	9	0	0	0	0	14	0.27	0.07
7	Q	-1	-1	3	1	-3	2	1	-1	-1	-3	-3	1	-2	-3	2	3	1	-4	-3	-3	0	0	17	6	0	12	7	0	0	0	0	7	0	0	11	33	7	0	0	0	0.38	0.09
8	K	-2	2	2	3	-4	0	2	-2	-1	-3	-1	4	-2	-3	-2	-1	-1	-4	-3	-3	0	6	9	18	0	0	12	0	0	0	7	48	0	0	0	0	0	0	0	0	0.43	0.11
9	X	-1	-1	-1	-1	-1	-1	-1	-1	-1	-1	-1	-1	-1	-1	-1	-1	-1	-1	-1	-1	6	0	0	0	0	0	0	0	0	28	34	0	0	7	0	0	7	0	0	13	0.00	0.00
10	L	-2	-3	-3	-3	-2	-2	0	-4	-3	2	4	-2	4	0	-3	-3	-2	-3	-2	0	0	0	0	0	0	0	10	0	0	14	59	0	17	0	0	0	0	0	0	0	0.46	0.12
11	S	0	-2	0	1	-2	0	2	-1	-2	-4	-4	-1	-3	-4	-2	5	0	-4	-3	-3	0	0	0	8	0	0	16	0	0	0	0	0	0	0	0	76	0	0	0	0	0.66	0.17
12	Y	-2	-1	-2	-1	-3	0	3	-3	-1	-1	1	1	2	1	-3	-2	-2	-1	4	-2	0	0	0	0	0	0	31	0	0	0	16	15	8	6	0	0	0	0	23	0	0.30	0.10
13	D	-1	1	3	4	-3	0	1	-2	-1	-3	-3	1	-2	-4	-2	0	-1	-4	-3	-1	6	8	24	34	0	0	8	0	0	0	0	13	0	0	0	0	0	0	0	7	0.43	0.09
14	R	1	1	-1	0	-2	-1	-1	0	2	-2	-2	0	1	-1	2	-1	-1	5	2	-1	19	9	0	8	0	0	0	6	7	0	0	7	8	0	10	0	0	11	9	7	0.19	0.06
15	I	-1	-2	-3	-3	-2	-2	-3	-3	-3	3	3	-1	1	1	-3	0	-1	-2	-1	2	0	0	0	0	0	0	0	0	0	28	35	7	0	8	0	10	0	0	0	12	0.25	0.08
16	L	-2	-3	-3	-4	-2	-2	-3	-4	-2	3	1	-3	4	2	-4	-2	0	6	2	0	0	0	0	0	0	0	0	0	0	30	11	0	19	12	0	0	8	14	6	0	0.46	0.12
17	N	-1	1	3	-1	-3	-1	-1	2	2	0	-1	-1	-1	-1	-3	1	-1	-2	2	-2	0	9	19	0	0	0	0	17	6	8	8	0	0	0	0	18	0	0	13	0	0.17	0.08
18	F	-2	-3	-4	-4	2	-3	-4	-4	-3	2	1	-4	4	5	-4	-3	-2	-1	1	2	0	0	0	0	5	0	0	0	0	13	7	0	16	44	0	0	0	0	0	14	0.58	0.16
19	V	-2	-3	0	-3	2	-3	-3	-4	-3	3	2	-3	0	1	-3	-2	-1	-3	-1	3	0	0	7	0	7	0	0	0	0	28	19	0	0	8	0	0	0	0	0	32	0.37	0.12
20	I	-2	-4	-3	-1	-2	-3	-3	-4	-4	5	1	-3	2	-1	-3	-3	0	-4	-2	3	0	0	0	5	0	0	0	0	0	57	4	0	4	0	0	0	7	0	0	22	0.55	0.17
21	G	-1	-3	-1	-2	-3	-2	-2	5	-3	-4	-4	-2	-3	0	-3	2	2	-3	-3	-3	0	0	0	0	0	0	0	55	0	0	0	0	0	6	0	25	14	0	0	0	0.71	0.18
22	A	2	-1	3	-1	-3	0	1	2	-1	-3	-3	1	0	-3	-2	1	-1	-4	-3	-2	21	0	24	0	0	0	10	14	0	0	0	14	5	0	0	12	0	0	0	0	0.30	0.11
23	R	-3	7	-2	-3	-5	0	-1	-4	-2	-5	-4	2	-3	-4	-4	-2	-3	-4	-3	-4	0	92	0	0	0	0	0	0	0	0	0	8	0	0	0	0	0	0	0	0	1.48	0.31
24	G	0	-2	3	0	-3	-2	-2	5	-2	-4	-4	0	-3	-4	-3	0	-2	-4	-4	-4	6	0	16	5	0	0	0	59	0	0	0	7	0	0	0	7	0	0	0	0	0.76	0.17
25	I	1	-2	1	0	-2	1	0	-1	-2	2	0	-1	0	-2	-2	0	0	-3	-2	1	13	0	11	7	0	8	6	5	0	20	9	0	0	0	0	6	6	0	0	10	0.07	0.05
26	G	-1	-4	-2	-3	-4	-3	-4	7	-4	-5	-5	-3	-4	-5	-4	-2	-3	-4	-5	-5	0	0	0	0	0	0	0	100	0	0	0	0	0	0	0	0	0	0	0	0	1.71	0.30
27	K	-2	1	-1	-2	-4	0	0	-3	-2	-4	-4	7	-3	-4	-2	-1	-2	-4	-3	-3	0	0	0	0	0	0	0	0	0	0	1	94	0	0	0	0	0	0	0	0	1.16	0.28
28	S	-1	-2	-1	-2	-2	-1	-2	-2	-2	-2	-3	-1	-2	-3	-2	3	5	-3	-3	-2	0	0	0	0	0	0	0	0	0	0	1	0	0	0	0	32	63	0	0	0	0.77	0.22
29	Y	-3	-3	-3	-4	-3	-3	-3	-4	0	-2	-2	-3	-2	4	-4	-2	0	1	8	-2	0	0	0	0	0	0	0	0	0	0	1	0	0	20	0	0	7	0	67	0	1.22	0.26
30	A	2	-2	1	-1	-2	0	-1	3	-2	-3	-3	-1	-2	0	-2	2	0	-3	-2	-2	18	0	7	0	0	5	0	33	0	0	1	0	0	7	0	21	6	0	0	0	0.34	0.10
31	X	-1	-1	-1	-1	-1	-1	-1	-1	-1	-1	-1	-1	-1	-1	-1	-1	-1	-1	-1	-1	14	0	0	0	0	0	0	4	0	12	8	0	0	15	0	0	22	15	0	0	0.00	0.00
32	K	0	0	1	-2	-3	0	-1	-2	-2	-2	1	4	-1	-2	-2	-1	0	4	-2	-2	7	0	7	0	0	0	0	0	0	0	19	48	0	0	0	0	7	8	0	0	0.35	0.14
33	V	-2	3	1	-1	-3	0	2	-3	4	0	-2	2	-1	-2	-2	-1	-1	-2	1	-1	0	17	6	0	0	0	14	0	15	13	1	22	0	0	0	0	0	0	6	4	0.26	0.10
34	Y	-2	2	-2	-3	-3	-1	-2	-3	4	-1	0	1	-1	1	-3	-2	-2	6	4	-2	0	13	0	0	0	0	0	0	16	5	12	10	0	5	0	0	0	12	22	0	0.44	0.13
35	P	-1	-2	-2	-3	2	-2	-2	-3	-2	2	1	-2	3	3	0	1	-1	-2	0	1	0	0	0	0	5	0	0	0	0	17	7	0	12	20	4	19	0	0	0	13	0.19	0.10
36	I	-1	-3	-3	-3	2	-2	-3	-3	-2	4	2	-3	1	0	-3	-2	-1	-2	2	2	1	1	1	1	5	1	1	1	0	37	24	1	0	1	1	1	1	0	9	14	0.31	0.10
37	N	-2	0	4	0	-4	0	3	-2	-1	-1	-3	4	-2	-3	1	-1	-1	-4	-3	-3	0	0	26	0	0	0	24	0	0	5	1	34	0	0	6	0	0	0	0	0	0.47	0.15
38	R	1	2	0	2	-3	1	1	0	-1	-2	0	1	-1	-2	-2	-1	-1	3	-2	-2	14	18	5	19	0	6	5	7	0	0	12	6	0	0	0	0	0	6	0	0	0.19	0.07
39	F	0	-3	-3	-3	-2	-3	-3	0	-1	1	1	-3	0	5	-3	-2	-2	0	4	-1	13	0	0	0	0	0	0	7	0	13	14	0	0	33	0	0	0	0	16	0	0.36	0.12
40	I	0	-1	-2	-2	-2	-1	1	-3	-2	1	1	0	0	3	-2	-1	0	-2	0	1	8	0	0	0	0	0	13	0	0	15	20	10	0	19	0	0	6	0	0	6	0.12	0.07
41	K	-2	0	2	1	-4	2	2	-2	-1	-3	-3	4	-2	-4	2	-1	-1	-4	-3	-3	0	0	11	6	0	8	16	0	0	0	1	46	0	0	9	0	0	0	0	0	0.51	0.14
42	Y	-1	1	2	1	-3	1	0	-2	1	-2	-2	2	-2	-1	-2	0	2	-2	3	-2	0	5	13	7	0	6	0	0	4	0	1	17	0	0	0	6	22	0	15	0	0.25	0.10
43	G	-1	1	1	1	-3	1	1	5	-2	-4	-4	-1	-3	-4	-2	-1	-2	-3	-3	-3	0	9	5	6	0	7	12	57	0	0	1	0	0	0	0	0	0	0	0	0	0.66	0.15
44	E	-2	1	-1	0	-4	2	4	-3	-1	-4	-3	3	-2	0	-2	0	-2	-3	1	-3	0	6	0	0	0	9	42	0	0	0	0	25	0	7	0	6	0	0	5	0	0.51	0.16
45	Q	0	3	-1	-1	-4	5	2	-3	-1	-4	-3	3	-2	-4	-2	-1	-2	-3	-3	-3	8	18	0	0	0	41	12	0	0	0	0	21	0	0	0	0	0	0	0	0	0.66	0.19
46	F	0	-4	-4	-4	-3	-4	-4	-4	-3	-1	1	-4	-1	7	-4	-1	-3	-1	1	-2	12	0	0	0	0	0	0	0	0	0	12	0	0	72	0	4	0	0	0	0	0.97	0.24
47	I	-1	-3	-4	-4	-2	-3	-4	-4	-4	4	2	-3	0	2	-4	-3	-2	4	-1	2	6	0	0	0	0	0	0	0	0	39	19	0	0	10	0	0	0	8	0	18	0.46	0.13
48	Y	-3	-3	-4	-5	-3	-3	-4	-4	-1	0	1	-4	-1	3	-5	-3	-3	9	6	-2	0	0	0	0	0	0	0	0	0	5	15	0	0	13	0	0	0	29	37	0	1.23	0.24
49	V	-1	-3	-4	-4	-2	-3	-4	-4	-4	2	4	-3	1	-1	-4	-3	-2	-3	-2	2	5	0	0	0	0	0	0	0	0	13	63	0	0	0	0	0	0	0	0	19	0.58	0.16
50	R	-1	7	-2	-3	-4	0	1	-3	-1	-4	-3	1	-3	-3	-3	-2	-2	-3	1	-4	7	78	0	0	0	0	8	0	0	0	0	0	0	0	0	0	0	0	7	0	1.05	0.26
51	R	-3	7	-2	-3	-4	-1	-2	-4	-2	0	-3	1	-2	-4	-4	-2	-2	-4	-3	0	0	84	0	0	0	0	0	0	0	8	0	0	0	0	0	0	0	0	0	8	1.13	0.28
52	Y	-1	-2	0	-2	-3	0	-2	-3	-1	-2	-2	-2	-2	3	-3	1	3	-1	5	-2	0	0	6	0	0	6	0	0	0	0	0	0	0	13	0	14	30	0	31	0	0.49	0.18
53	K	-1	0	-1	2	-3	3	0	-2	-2	-3	-1	4	-2	-4	1	-1	1	-4	-3	-2	5	0	0	13	0	22	0	0	0	0	5	35	0	0	6	0	14	0	0	0	0.38	0.15
54	P	-1	-2	1	2	-2	-1	0	0	-1	-3	-3	0	-2	-2	0	2	3	-3	1	-2	0	0	5	11	0	0	6	6	0	0	0	5	0	0	3	22	35	0	7	0	0.32	0.13
55	E	-2	-1	-1	4	-4	2	5	-3	-1	-4	-4	-1	-3	-3	-2	1	-1	-3	1	-3	0	0	0	25	0	10	46	0	0	0	0	0	0	0	0	12	0	0	7	0	0.68	0.18
56	L	0	-2	-3	-3	-2	-2	-3	-3	-3	2	3	0	2	-1	-3	-1	-1	-3	-2	1	10	0	0	0	0	0	0	0	0	14	47	7	8	0	0	6	0	0	0	8	0.30	0.11
57	A	0	1	-1	2	-4	4	3	-2	-1	-3	-3	2	-2	0	-2	-1	0	-3	-2	-3	7	5	0	11	0	24	28	0	0	0	0	12	0	7	0	0	6	0	0	0	0.41	0.13
58	K	1	0	-1	-2	-2	-1	0	0	-2	-1	-2	0	-2	1	2	3	0	-3	-2	-2	15	5	0	0	0	0	4	4	0	7	0	7	0	8	12	39	0	0	0	0	0.26	0.12
59	V	1	0	-2	-2	-2	-1	0	-2	-2	1	1	0	1	-1	-2	-1	-1	5	-1	2	20	5	0	0	0	0	7	0	0	7	15	5	5	0	0	0	0	12	0	21	0.20	0.08
60	S	2	2	0	-1	-3	1	1	-2	-1	-3	-1	3	-2	-3	1	0	-1	-3	-2	-2	28	11	4	0	0	7	8	0	0	0	6	26	0	0	6	3	0	0	0	0	0.27	0.10
61	N	0	1	3	3	-3	-1	0	0	-1	-3	-3	2	-2	-4	-2	0	2	-4	-3	-2	7	6	21	19	0	0	0	5	0	0	0	18	0	0	0	4	20	0	0	0	0.35	0.12
62	Y	-1	-3	-4	-4	-2	-3	-3	-4	-2	2	0	-3	0	4	-4	-3	-2	5	4	2	5	0	0	0	0	0	0	0	0	17	6	0	0	24	0	0	0	8	21	19	0.51	0.14
63	F	-2	-3	0	-3	-3	-3	-3	-1	-2	1	2	-3	0	6	-4	-2	0	-1	1	-1	0	0	7	0	0	0	0	6	0	6	24	0	0	49	0	0	8	0	0	0	0.52	0.17
64	N	-1	0	5	4	-4	-1	2	-2	-1	-3	-3	-1	1	-4	-3	-1	0	-4	-3	-3	4	6	37	29	0	0	11	0	0	0	0	0	8	0	0	0	5	0	0	0	0.56	0.16
65	D	-2	-1	1	4	-4	-1	0	0	-2	-3	-2	2	-2	-4	3	1	-1	-4	-3	-3	0	0	6	35	0	0	0	7	0	0	6	22	0	0	14	9	0	0	0	0	0.46	0.14
66	V	-2	0	-3	-4	-2	-3	-3	-4	-2	2	1	-3	0	3	-4	-3	-2	-1	4	3	0	6	0	0	0	0	0	0	0	15	14	0	0	16	0	0	0	0	20	28	0.39	0.13
67	A	0	-1	1	-2	4	1	-1	-2	-2	0	1	1	0	0	-2	0	0	-3	-1	0	8	0	13	0	12	11	0	0	0	5	19	10	0	7	0	5	6	0	0	6	0.10	0.07
68	Q	0	2	1	-1	-3	3	2	-2	2	-4	-3	2	-2	-4	1	1	-1	-3	-2	-3	5	11	8	0	0	20	14	0	8	0	0	17	0	0	6	13	0	0	0	0	0.37	0.11
69	E	-1	-1	0	3	-3	1	3	0	2	-2	-1	0	-2	-2	-2	-1	0	-2	1	-2	1	0	0	20	0	7	29	6	6	0	8	5	0	0	0	1	9	0	7	1	0.28	0.09
70	F	-2	0	1	-2	-3	-2	-3	0	-1	-1	0	-2	-1	5	-3	-2	-2	0	4	0	1	5	12	0	0	0	0	9	0	0	7	0	0	40	0	1	0	0	15	7	0.40	0.13
71	P	-1	-1	-2	-1	-3	1	2	-3	-2	-3	-1	1	-2	-3	6	-1	0	-3	-3	-2	1	0	0	0	0	9	15	1	0	0	7	12	0	0	46	1	7	0	0	1	0.83	0.15
72	D	0	-1	-1	2	-3	2	3	0	-1	-2	0	1	-1	-3	-2	-1	0	-3	-2	-2	6	0	0	13	0	9	30	8	0	0	13	12	0	0	0	1	5	0	0	1	0.25	0.09
73	H	-1	-2	-1	1	-2	-1	0	-3	3	2	-1	0	1	-1	-2	-1	0	4	2	1	1	0	0	12	0	0	5	1	10	17	1	7	5	0	0	1	5	6	12	17	0.15	0.08
74	E	-2	-1	3	2	-4	0	5	-3	-1	-4	-4	0	-3	0	3	-1	-2	-4	-2	-3	0	0	17	9	0	0	49	0	0	0	0	5	0	7	13	0	0	0	0	0	0.63	0.18
75	L	0	-2	-3	-3	-2	-2	-3	-3	-2	1	1	-1	2	4	-3	-1	1	-1	2	1	11	0	0	0	0	0	0	0	0	10	16	6	7	26	0	0	12	0	5	6	0.22	0.09
76	V	-1	1	-1	-2	-2	0	1	-3	3	1	0	1	-1	-1	-2	0	0	-2	1	1	0	9	0	0	0	0	15	0	11	17	6	8	0	0	0	6	7	0	8	12	0.12	0.08
77	V	0	-3	-3	-3	-2	-2	-1	-3	-3	3	2	-2	0	-1	-3	0	0	-3	-2	4	7	0	0	0	0	0	5	0	0	16	16	0	0	0	0	6	6	0	0	44	0.31	0.12
78	K	-2	3	1	-1	-4	0	1	-3	-1	0	-3	5	-2	-4	-2	0	-1	-4	-3	-2	0	18	7	0	0	0	10	0	0	11	0	50	0	0	0	5	0	0	0	0	0.50	0.16
79	G	1	-3	-1	-2	-3	-2	-3	6	-3	-4	-4	-2	-3	-4	-3	1	1	-4	-4	-3	8	0	0	0	0	0	0	67	0	0	0	0	0	0	0	13	11	0	0	0	0.91	0.20
80	R	-2	3	2	2	-3	0	0	-1	-1	-1	-2	1	-2	2	-3	-1	0	-2	1	-2	0	23	13	12	0	0	6	5	0	6	0	12	0	14	0	0	6	0	4	0	0.21	0.09
81	R	0	1	-2	-1	-3	1	2	0	-2	-1	-1	3	2	3	-2	-1	-2	-2	-1	-2	7	3	0	0	0	4	16	6	0	5	0	31	8	19	0	0	0	0	0	0	0.22	0.12
82	F	-1	-2	-2	-2	-2	-2	0	0	-1	1	0	-2	1	3	-3	-1	0	-1	3	1	0	0	0	0	0	0	9	10	0	8	7	0	7	18	0	5	6	0	13	16	0.13	0.07
83	Y	-2	-2	-3	-3	-3	0	1	-3	0	-2	-2	0	-2	3	-3	-2	-2	4	6	0	0	0	0	0	0	5	11	0	0	0	1	6	0	13	0	0	0	6	46	9	0.64	0.17
84	I	-2	-2	-3	-4	-3	-2	-3	-4	-1	4	-1	1	-1	0	-4	-3	-2	-1	6	1	0	0	0	0	0	0	0	0	0	38	0	15	0	0	0	0	0	0	38	8	0.60	0.20
85	D	-2	1	2	4	-4	1	0	-2	-1	-4	-4	3	-3	-3	-2	0	-1	-3	1	-3	0	5	13	37	0	6	0	0	0	0	0	25	0	0	0	6	0	0	8	0	0.51	0.15
86	G	-1	-1	2	2	-3	1	2	3	-1	-4	-3	0	-3	0	-2	0	-1	3	-2	-3	0	0	12	13	0	8	19	26	0	0	0	6	0	5	0	7	0	4	0	0	0.35	0.10
87	K	-2	0	-1	0	-4	3	4	-3	-1	0	-2	3	1	-3	-2	-1	0	-4	-3	-1	0	0	0	0	0	13	41	0	0	6	0	24	5	0	0	0	6	0	0	6	0.45	0.16
88	L	-1	-3	-2	-3	3	-2	-2	-1	2	1	1	-2	3	-2	2	0	0	-3	-2	2	0	0	0	0	10	0	0	5	8	6	12	0	17	0	12	6	4	0	0	20	0.22	0.10
89	A	0	0	-3	-3	5	-2	-3	-3	-3	3	0	0	-1	1	0	-1	-1	-3	-1	1	11	6	0	0	18	0	0	0	0	33	0	6	0	9	5	5	0	0	0	6	0.25	0.12
90	G	-1	-3	-2	-3	5	-3	-3	5	-3	0	0	-3	-2	-3	-3	-1	-2	-3	-3	-2	0	0	0	0	16	0	0	60	0	7	13	0	0	0	0	0	0	0	0	0	0.73	0.17
91	W	-2	0	-3	-3	-3	-2	-2	-3	2	-1	1	0	-1	2	-3	-2	-2	6	5	0	0	6	0	0	0	0	0	0	6	0	20	7	0	6	0	0	0	11	31	9	0.45	0.13
92	A	1	-3	-3	-4	2	-3	-3	-1	-2	1	0	-3	1	5	-4	-2	-2	-1	3	-1	13	0	0	0	6	0	0	6	0	13	5	0	6	37	0	0	0	0	13	0	0.40	0.14
93	I	0	-3	-2	-3	-2	-2	-3	-1	-3	4	0	-2	0	1	-3	1	1	-3	-2	1	8	0	0	0	0	0	0	5	0	44	0	0	0	6	0	19	12	0	0	6	0.26	0.13
94	P	2	-2	-1	1	-2	-1	2	0	2	-3	-3	-1	-2	-3	3	2	-1	-4	-2	-2	27	0	0	7	0	0	18	5	8	0	0	0	0	0	16	20	0	0	0	0	0.39	0.12
95	L	-3	-3	-5	-5	-2	-3	-4	-5	-4	3	5	-4	1	-1	-4	-4	-2	-3	-2	0	0	0	0	0	0	0	0	0	0	19	81	0	0	0	0	0	0	0	0	0	0.84	0.21
96	S	-1	-1	4	-1	-3	-1	0	-2	-1	-3	-3	1	-2	0	-2	4	1	-3	1	-3	0	0	25	0	0	0	6	0	0	0	0	8	0	6	0	45	5	0	6	0	0.43	0.16
97	V	0	-1	1	-2	2	1	-1	0	1	-1	0	0	1	-2	-2	1	2	-3	-2	0	5	0	8	0	7	8	0	5	6	0	10	6	5	0	0	9	21	0	0	10	0.11	0.07
98	W	3	-2	0	-2	-2	-2	-2	-2	-2	-2	-1	-2	-2	0	1	2	-1	6	1	-2	39	0	5	0	0	0	0	0	0	0	8	0	0	5	8	16	0	13	6	0	0.38	0.14
99	Q	-1	-1	3	1	-3	3	2	-2	2	-4	-3	-1	-2	-2	-2	1	-1	-2	2	-3	0	0	24	7	0	19	17	0	5	0	0	0	0	0	0	16	0	0	12	0	0.44	0.14
100	S	0	1	1	2	-3	2	0	-2	-1	-3	-2	3	-2	-3	-2	1	1	-4	-3	-2	8	5	6	12	0	12	0	0	0	0	5	34	0	0	0	10	8	0	0	0	0.30	0.11
101	E	1	-2	-1	-2	-2	-1	0	-2	-2	2	2	-1	2	1	-3	-1	-1	-2	1	1	23	0	4	0	0	0	7	0	0	17	21	4	8	5	0	0	0	0	5	6	0.12	0.08
102	K	-2	5	-1	-2	-5	0	-1	-3	-2	-4	-4	5	-3	-4	-3	-2	-2	-4	-3	-4	0	39	0	0	0	0	0	0	0	0	0	61	0	0	0	0	0	0	0	0	1.04	0.25
103	S	0	1	-1	-1	-3	-1	1	3	1	-4	-4	-1	-3	-4	-2	4	0	-4	-3	-3	0	8	0	0	0	0	12	23	5	0	0	0	0	0	0	52	0	0	0	0	0.51	0.18
104	N	1	-2	1	-2	2	-2	-2	-2	1	2	1	-2	0	-2	0	1	0	-3	-2	1	13	0	10	0	5	0	0	0	7	17	20	0	0	0	5	14	3	0	0	6	0.11	0.07
105	A	0	-2	-1	2	-2	1	0	2	-1	-1	0	-1	-1	0	-2	1	-1	-2	1	-1	8	0	0	14	0	8	5	20	0	0	13	0	0	6	0	14	0	0	6	7	0.12	0.07
106	Y	-2	-2	0	1	-3	-2	0	-3	-1	-1	0	-2	3	4	-3	-2	0	-1	4	-2	0	0	6	12	0	0	8	0	0	0	7	0	12	25	0	0	6	0	24	0	0.38	0.14
107	P	-2	1	2	-1	-4	2	-1	0	-2	-4	-4	0	-3	-4	6	0	0	-4	-3	-3	0	7	15	0	0	14	0	6	0	0	0	6	0	0	41	6	5	0	0	0	0.79	0.16
108	N	-2	-2	5	3	-4	-1	1	0	-1	-3	-2	-1	-3	-2	-3	-1	0	-3	2	-3	0	0	40	22	0	0	7	8	0	0	5	0	0	0	0	6	0	11	0	0	0.51	0.16
109	V	1	-3	-3	-4	-2	-3	-3	-3	-2	2	0	-3	0	1	1	-2	-1	-2	3	4	13	0	0	0	0	0	0	0	0	14	0	0	0	6	6	0	0	0	15	46	0.38	0.15
110	S	-1	0	1	1	-3	1	0	-2	-1	-2	-3	3	-2	-2	-2	2	1	-3	2	-1	0	0	6	10	0	7	3	0	0	0	0	27	0	0	0	24	8	0	9	5	0.27	0.11
111	T	-2	1	0	-2	-2	-1	-2	-3	4	-1	1	0	-1	1	-3	-1	2	-1	3	-1	0	7	5	0	0	0	0	0	14	0	20	5	0	5	0	0	23	0	18	0	0.24	0.11
112	I	-2	-4	-4	-4	-2	-4	-4	-5	-4	6	0	-4	2	-1	-4	-3	-2	-4	-2	3	0	0	0	0	0	0	0	0	0	79	0	0	7	0	0	0	0	0	0	15	0.92	0.24
113	V	-2	-3	-4	-4	-2	-3	-4	-4	-3	3	3	-3	1	3	-4	-3	-2	-2	-1	3	0	0	0	0	0	0	0	0	0	27	29	0	0	18	0	0	0	0	0	25	0.50	0.14
114	F	-3	-4	-4	-5	-3	-4	-4	-4	-2	1	0	-4	2	7	-5	-3	-3	0	5	-1	0	0	0	0	0	0	0	0	0	8	5	0	6	60	0	0	0	0	21	0	1.01	0.23
115	D	-3	-3	0	7	-5	-1	2	-3	-2	-5	-5	-2	-4	-5	-3	-2	-2	-6	-4	-5	0	0	0	87	0	0	13	0	0	0	0	0	0	0	0	0	0	0	0	0	1.48	0.29
116	E	-2	-2	-2	0	-5	1	7	-4	-2	-5	-5	-1	-4	-5	-3	-2	-2	-5	-4	-4	0	0	0	0	0	0	100	0	0	0	0	0	0	0	0	0	0	0	0	0	1.50	0.33
117	F	0	-4	-4	-4	3	-4	-4	-4	-2	0	-1	-4	-1	6	-4	-3	-2	-1	3	0	12	0	0	0	8	0	0	0	0	6	0	0	0	59	0	0	0	0	9	5	0.79	0.21
118	I	-2	-3	0	-3	-2	-2	-3	-3	-3	4	2	-2	2	-1	-3	-2	-1	-3	-2	2	1	1	9	1	0	1	1	1	0	50	19	1	5	0	1	1	1	0	0	9	0.36	0.12

119	R	1	0	0	-2	2	0	-1	-2	-2	1	-2	-1	-1	-3	5	0	0	-3	-2	-1	15	7	6	0	5	6	0	1	0	14	1	0	0	0	30	7	7	0	0	0	0.41	0.10
120	E	-1	0	-1	2	-3	1	4	-3	-1	-2	-1	0	-2	1	1	-1	1	-3	-2	-2	5	6	0	15	0	7	36	0	0	0	7	0	0	8	6	0	10	0	0	0	0.32	0.12
121	K	0	0	-1	2	-3	1	0	-2	-1	-2	-1	4	-2	-3	1	0	-1	-3	-2	-2	6	1	1	13	0	6	1	1	0	1	7	48	0	0	6	8	1	0	0	1	0.34	0.12
122	D	-1	-1	1	1	-2	0	2	0	-1	-1	-1	1	-1	-2	-1	1	2	-2	-2	-1	2	1	6	8	1	1	25	6	1	1	9	7	1	1	2	7	18	0	1	2	0.16	0.05
123	N	-1	-1	4	1	-2	-1	-1	2	-1	-2	-1	1	-2	-2	-2	1	1	-3	-2	-2	1	1	32	7	0	1	1	17	0	1	7	9	0	1	1	10	8	0	1	1	0.28	0.08
124	S	-1	0	1	-1	-3	0	1	3	2	-3	-1	0	-2	-2	-3	1	-1	5	-1	-3	0	5	7	0	0	5	11	24	8	0	10	4	0	0	0	15	0	11	0	0	0.29	0.10
125	N	-2	3	2	2	-4	1	0	-1	2	-3	-3	2	-2	0	1	-1	-2	-2	1	-3	0	18	13	13	0	6	0	5	7	0	0	16	0	6	8	0	0	0	8	0	0.28	0.10
126	Y	-3	-3	-4	-4	-4	-3	-4	0	0	0	-2	-3	-2	3	-4	-3	-3	1	8	-2	0	0	0	0	0	0	0	8	0	5	0	0	0	8	0	0	0	0	79	0	1.40	0.31
127	I	0	-3	-3	-4	-2	-3	-3	-3	-3	2	4	-3	1	-1	-3	0	0	-3	-2	1	7	0	0	0	0	0	0	0	0	15	56	0	0	0	0	8	7	0	0	8	0.38	0.13
128	P	-1	1	-1	0	-4	0	-1	0	-2	-4	-4	4	-3	-4	4	0	0	-4	-3	-3	0	5	0	6	0	0	0	8	0	0	0	44	0	0	22	8	7	0	0	0	0.57	0.16
129	N	-3	-2	5	6	-4	-1	1	-2	-1	-4	-5	-1	-4	-5	-3	0	-2	-5	-4	-4	0	0	39	51	0	0	6	0	0	0	0	0	0	0	0	5	0	0	0	0	1.01	0.23
130	E	-2	-2	-1	2	-5	1	6	-3	-1	-5	-4	-1	-3	-5	-2	0	-2	-4	-3	-4	0	0	0	8	0	0	84	0	0	0	0	0	0	0	0	8	0	0	0	0	1.15	0.27
131	V	-1	-2	-3	-3	-2	0	-2	-3	-2	0	1	-2	1	1	4	-2	0	-2	1	2	4	0	0	0	0	6	0	0	0	0	13	0	4	6	27	0	8	0	5	26	0.39	0.12
132	S	-1	1	1	-1	-3	0	2	-3	2	2	-1	1	-1	-2	-2	1	0	-3	-2	0	0	8	7	0	0	0	24	0	6	21	0	13	0	0	0	12	5	0	0	5	0.18	0.10
133	A	0	0	1	-2	-3	0	0	-2	-2	-2	1	4	1	-3	-2	-1	-1	-4	-3	-1	11	0	8	0	0	0	5	0	0	0	15	50	6	0	0	0	0	0	0	4	0.33	0.15
134	L	-3	-3	-4	-5	-3	-3	-4	-5	-3	1	4	-4	1	5	-4	-3	-2	-1	2	0	0	0	0	0	0	0	0	0	0	6	57	0	0	31	0	0	0	0	6	0	0.69	0.18
135	L	-1	0	0	0	-2	-1	1	-3	-2	2	1	0	1	-1	-2	0	1	-3	0	0	0	6	4	6	0	0	10	0	0	24	13	7	4	0	0	8	12	0	5	0	0.08	0.07
136	N	-1	1	3	3	-3	0	2	-2	-1	-4	-4	-1	-3	-4	-2	3	1	-4	-3	-3	0	8	22	20	0	0	12	0	0	0	0	0	0	0	0	31	7	0	0	0	0.48	0.15
137	L	-2	-3	-4	-4	2	-3	-4	-5	-4	3	4	-4	1	2	-4	-3	-2	-2	-1	1	0	0	0	0	6	0	0	0	0	25	54	0	0	8	0	0	0	0	0	7	0.55	0.15
138	X	-1	-1	-1	-1	-1	-1	-1	-1	-1	-1	-1	-1	-1	-1	-1	-1	-1	-1	-1	-1	0	0	0	0	5	0	0	0	7	32	12	0	3	0	0	0	0	6	27	4	0.00	0.00
139	D	-1	-1	1	2	-3	1	2	1	-1	-2	-2	1	1	-3	-2	1	0	-3	-3	-1	0	0	6	17	0	7	20	12	0	0	0	8	8	0	0	11	7	0	0	5	0.24	0.08
140	T	0	-2	-1	1	-2	-1	-1	-2	-2	-3	-3	-2	-2	-4	-2	4	4	-4	-3	-2	0	0	0	8	0	0	0	0	0	0	0	0	0	0	0	49	43	0	0	0	0.71	0.23
141	V	1	-3	-3	-4	-2	-3	-3	-3	-4	4	1	-3	2	-1	-3	-2	0	-3	-2	3	12	0	0	0	0	0	0	0	0	35	7	0	7	0	0	0	6	0	0	33	0.41	0.14
142	F	1	-2	0	1	-3	2	-1	0	-2	0	-2	-2	-1	2	-3	-1	0	7	0	-1	17	0	7	9	0	13	0	6	0	10	0	0	0	13	0	0	8	18	0	0	0.32	0.12
143	R	-3	7	-2	-3	-5	0	-1	-4	-2	-5	-4	2	-3	-4	-4	-2	-3	-4	-3	-4	0	92	0	0	0	0	0	0	0	0	0	8	0	0	0	0	0	0	0	0	1.47	0.31
144	N	0	-2	2	2	-2	0	0	1	-1	-1	1	-1	1	0	-2	-1	0	-2	0	-1	8	0	15	12	0	5	6	15	0	3	16	0	6	5	0	0	4	0	4	0	0.09	0.06
145	R	-2	5	1	-1	-4	1	0	0	-1	-3	-3	2	-2	-3	-2	-1	0	-3	-2	-3	1	51	9	0	0	5	5	7	0	0	1	14	0	0	0	0	5	0	0	0	0.57	0.15
146	E	-2	-2	0	5	-4	0	4	0	-2	-4	-4	-1	-4	-4	2	-1	-2	-5	-4	-4	0	0	0	45	0	0	37	7	0	0	0	0	0	0	11	0	0	0	0	0	0.83	0.18
147	R	-1	2	1	4	-3	0	0	2	-1	-3	-3	2	-3	-3	0	-1	0	-4	-3	-3	1	14	7	33	0	0	0	18	0	0	1	14	0	0	5	0	5	0	0	0	0.39	0.10
148	V	-1	-2	-2	-3	2	-2	-2	-3	1	3	1	0	0	-2	1	-1	1	-3	-2	3	0	0	0	0	6	0	0	0	5	27	6	7	0	0	7	4	12	0	0	27	0.21	0.09
149	R	-2	4	-1	-2	-4	2	1	-3	-1	-3	-3	4	-2	-3	-2	-1	0	-3	1	-1	0	37	0	0	0	7	5	0	0	0	0	33	0	0	0	0	7	0	6	5	0.52	0.14
150	C	-2	-3	-4	-4	4	-3	-3	-4	-3	3	2	-1	0	-1	-3	-2	-1	-3	0	3	0	0	0	0	15	0	0	0	0	31	21	6	0	0	0	0	0	0	5	22	0.38	0.12
151	I	-2	-4	-4	-4	-2	-3	-4	-4	-3	4	2	-3	0	4	-4	-3	-2	-2	2	2	0	0	0	0	0	0	0	0	0	47	14	0	0	21	0	0	0	0	7	12	0.52	0.14
152	C	2	-3	-3	-4	7	-2	-3	-1	-3	-1	0	-3	4	1	-3	-1	-2	-2	1	-1	21	0	0	0	34	0	0	4	0	0	5	0	23	6	0	0	0	0	7	0	0.60	0.18
153	L	-2	-3	-4	-4	-2	-3	-3	-4	-3	3	3	-3	3	2	-3	-3	-1	-2	-1	2	0	0	0	0	0	0	0	0	0	21	39	0	9	9	0	0	0	0	0	19	0.40	0.10
154	S	3	-2	-1	-2	-2	-2	-2	4	-3	-3	-3	-2	-3	-4	-2	2	-1	-4	-3	-2	41	0	0	0	0	0	0	40	0	0	0	0	0	0	0	19	0	0	0	0	0.65	0.16
155	N	-3	-2	8	0	-4	-1	-2	-2	-1	-5	-5	-2	-4	-5	-4	-1	-1	-5	-4	-5	0	0	100	0	0	0	0	0	0	0	0	0	0	0	0	0	0	0	0	0	1.64	0.33
156	A	2	-1	3	-1	-2	2	-1	-1	-1	-3	-3	-1	-2	-4	4	2	1	-4	-3	-2	23	0	21	0	0	15	0	0	0	0	0	0	0	0	20	14	7	0	0	0	0.47	0.13
157	V	-1	-2	-1	1	-2	-2	-2	1	-2	1	-1	-2	-1	-1	-2	0	1	-2	1	3	1	0	0	9	0	0	1	13	0	6	1	1	0	0	0	7	11	0	7	43	0.16	0.09
158	S	0	-1	2	-1	-2	-1	1	-1	-1	0	-2	-1	-2	-3	-2	4	1	-3	-2	-2	1	0	14	0	0	0	7	1	0	9	1	1	0	0	0	56	7	0	0	1	0.41	0.14
159	V	0	0	-2	-3	-2	-1	-2	-3	1	2	1	0	0	3	-2	-2	-1	-1	2	0	9	7	0	0	0	0	1	1	5	21	13	7	0	19	0	1	1	0	10	4	0.14	0.06
160	V	-1	-2	-2	-3	-2	-2	-2	-1	-1	0	1	-2	2	2	-3	0	-1	0	5	2	1	0	0	0	0	0	1	6	0	0	13	1	11	7	0	8	1	0	29	21	0.27	0.10
161	N	-2	1	6	2	2	-1	-1	-1	-1	-3	-3	1	-3	-3	-2	1	-1	-4	-3	-3	1	7	56	9	7	0	1	1	0	0	1	7	0	0	0	8	1	0	0	1	0.62	0.15
162	P	-2	-3	-3	-3	-3	-2	-2	-3	-3	0	-3	-2	-3	-4	8	-2	-2	-4	-3	-2	1	0	0	0	0	0	1	1	0	9	1	1	0	0	83	1	1	0	0	1	1.89	0.25
163	Y	-3	-3	-3	-4	-3	-3	-3	-4	0	0	-1	-3	-1	5	-4	-3	-2	1	7	-2	1	0	0	0	0	0	1	1	0	6	1	1	0	27	0	1	1	0	60	1	1.06	0.21
164	F	-2	-2	0	-3	-3	-2	-3	-3	2	-1	0	-3	-1	6	-3	0	-2	0	3	-2	1	0	7	0	0	0	1	1	7	0	8	1	0	56	0	9	1	0	7	1	0.59	0.15
165	L	1	-1	1	2	-2	-1	1	-2	-2	1	0	0	-1	-2	-2	0	-1	-3	-2	1	20	0	7	17	0	0	10	1	0	10	8	9	0	0	0	1	1	0	0	14	0.11	0.05
166	F	1	0	-2	0	-2	-1	0	-2	0	-2	-1	-1	1	3	-2	-1	0	0	5	-1	18	5	0	7	0	0	7	1	0	0	1	1	7	14	0	1	6	0	31	1	0.28	0.10
167	F	-2	-3	-3	-4	-3	-3	-3	-3	-2	-1	2	-3	2	6	-4	-3	-2	4	3	-1	1	0	0	0	0	0	1	1	0	0	19	1	7	55	0	1	1	5	6	1	0.72	0.16
168	N	-1	2	2	3	-4	2	1	2	1	-4	-4	1	-2	-4	-2	0	-1	-4	-2	-3	0	14	11	19	0	16	5	19	5	0	0	6	0	0	0	4	0	0	0	0	0.37	0.09
169	L	-2	-2	-3	-4	-2	-2	-3	-4	-3	3	3	0	2	0	-3	-3	-1	-2	1	2	0	0	0	0	0	0	0	0	0	22	44	8	8	0	0	0	0	0	6	13	0.36	0.10
170	V	-1	1	-1	1	-3	0	1	-2	2	0	-1	1	-1	1	-2	1	-1	-2	1	0	0	6	0	12	0	0	11	0	8	8	0	13	0	11	0	15	0	0	6	9	0.11	0.06
171	P	-2	-2	2	-1	-3	-1	1	-3	-2	-3	-1	-2	-2	-4	7	-1	0	-4	-3	-3	0	0	14	0	0	0	9	0	0	0	9	0	0	0	59	0	8	0	0	0	1.13	0.16
172	D	-1	1	1	2	-2	0	1	-1	2	-3	-3	1	-2	-3	-1	2	1	-3	-2	-2	1	11	7	15	0	0	12	1	8	0	1	12	0	0	0	23	8	0	0	1	0.26	0.04
173	V	-1	-1	-1	-1	-2	0	1	-2	2	0	1	0	1	1	-2	0	2	-2	0	0	1	0	0	0	0	0	12	1	8	7	11	8	8	9	0	1	25	0	0	7	0.09	0.04
174	N	-1	2	0	-2	-2	-1	-1	-2	-2	-1	0	0	-1	-2	2	1	3	-3	-2	1	0	19	6	0	0	0	0	0	0	0	11	0	0	0	11	7	33	0	0	13	0.23	0.07
175	K	-1	2	1	1	-3	2	2	-2	-1	-2	0	4	-1	-3	-2	-1	-1	-3	-2	-2	0	11	8	8	0	9	10	0	0	0	13	41	0	0	0	0	0	0	0	0	0.32	0.07
176	R	-2	2	3	0	-4	1	3	-2	-1	-4	-3	2	-2	-4	2	-1	-1	-4	-3	-3	0	13	21	0	0	0	36	0	0	0	0	21	0	0	9	0	0	0	0	0	0.50	0.09
177	F	-1	-2	1	-2	-2	-2	-2	-2	-1	-1	-1	-2	-1	5	-2	0	1	0	3	-1	1	1	9	1	0	0	1	1	0	1	1	1	0	51	1	12	11	0	8	1	0.41	0.08
178	N	-1	1	3	-1	-2	-1	-1	0	-1	0	1	1	0	1	-3	0	-1	-2	-1	-1	0	9	23	0	0	0	0	8	0	8	22	11	0	11	0	8	0	0	0	0	0.10	0.05
179	V	-1	-1	-2	-3	-2	-1	-2	-3	2	0	2	1	0	3	-3	-1	2	-2	0	1	0	0	0	0	0	0	0	0	9	0	26	13	0	19	0	0	19	0	0	15	0.15	0.06
180	Y	-2	-3	-3	-4	-2	-3	-3	-4	-1	1	1	-3	0	4	-4	-3	-2	5	5	1	0	0	0	0	0	0	0	0	0	8	19	0	0	22	0	0	0	11	32	9	0.58	0.09
181	D	-2	3	0	3	-4	1	1	-2	-1	-4	-3	4	-2	-4	-2	-1	-1	-4	-3	-3	0	19	0	20	0	0	7	0	0	0	0	55	0	0	0	0	0	0	0	0	0.59	0.11
182	D	-1	-1	2	4	-3	1	4	-2	-1	-4	-4	1	-3	-4	-2	1	-1	-4	-3	-3	0	0	10	25	0	0	39	0	0	0	0	9	0	0	0	17	0	0	0	0	0.52	0.08
183	A	0	0	2	1	-3	0	2	-2	-1	-1	-2	3	1	-3	-2	0	-1	-3	-2	-1	7	0	15	9	0	0	18	0	0	8	0	34	10	0	0	0	0	0	0	0	0.24	0.06
184	L	0	-2	-1	2	3	-2	-1	-2	-2	0	2	-2	0	-1	-2	1	-1	-3	-2	-1	8	0	0	16	10	0	0	0	0	0	44	0	0	0	0	21	0	0	0	0	0.18	0.05
185	I	-1	-3	-4	-4	-2	-3	-3	-4	-3	4	2	-3	3	0	-3	-3	-1	-3	-2	3	0	0	0	0	0	0	0	0	0	54	16	0	12	0	0	0	0	0	0	18	0.44	0.07
186	E	0	1	-1	-1	-2	0	2	1	-2	-1	1	0	0	-2	-2	-1	-1	-3	-2	0	12	14	0	0	0	0	26	16	0	0	21	0	0	0	0	0	0	0	0	11	0.13	0.04
187	I	-1	-3	-4	-4	-2	-3	-3	-4	-4	4	2	-3	1	-1	-3	-3	-1	-3	-2	3	0	0	0	0	0	0	0	0	0	49	16	0	0	0	0	0	0	0	0	35	0.46	0.07
188	P	-1	0	-1	-1	-3	0	-1	2	-2	-3	-3	2	-2	-3	5	0	1	-3	-3	-2	0	0	0	0	0	0	0	20	0	0	0	27	0	0	32	0	20	0	0	0	0.56	0.02
189	D	-2	-2	1	5	-4	0	1	-2	4	-4	-4	-1	-3	-3	3	-1	-1	-4	-2	-3	0	0	0	63	0	0	0	0	20	0	0	0	0	0	17	0	0	0	0	0	0.72	0.04
190	S	0	-1	0	-1	-1	-1	-1	-1	-1	-2	-2	-1	-1	-3	-1	3	4	-3	-2	-1	0	0	0	0	0	0	0	0	0	0	0	0	0	0	0	45	55	0	0	0	0.42	0.02
191	L	1	-1	-2	-2	-2	2	-1	-2	-1	0	1	-1	0	3	-2	-1	-1	-1	1	-1	23	0	0	0	0	26	0	0	0	0	22	0	0	28	0	0	0	0	0	0	0.15	0.01
192	D	-2	-1	1	5	-4	1	4	-2	-1	-3	-4	0	-3	-4	-1	0	-1	-4	-3	-3	0	0	0	62	0	0	38	0	0	0	0	0	0	0	0	0	0	0	0	0	0.73	0.01
193	F	-2	-3	-3	-4	-3	-3	-3	-3	0	-1	0	-3	0	6	-4	-2	-2	2	5	-1	0	0	0	0	0	0	0	0	0	0	0	0	0	62	0	0	0	0	38	0	0.70	0.01
194	S	1	-1	1	0	-1	0	0	0	-1	-2	-2	0	-1	-2	-1	4	1	-3	-2	-2	0	0	0	0	0	0	0	0	0	0	0	0	0	0	0	0	0	0	0	0	0.00	0.00

	K	Lambda
Standard Ungapped	0.1414	0.3234
Standard Gapped	0.0422	0.2670
PSI Ungapped	0.1378	0.3180
PSI Gapped	0.0422	0.2670

PSSM #3

Query protein:
Myoglobin from *Physeter macrocephalus*
(PDB 1MBN, chain A)

Target dataset:
UniRef90-2015, 38.20 million proteins
(full size)

PSSM generator:
PSI-BLAST

(see the next page)

Last position-specific scoring matrix computed, weighted observed percentages rounded down, information per position, and relative weight of gapless real matches to pseudocounts

	A	R	N	D	C	Q	E	G	H	I	L	K	M	F	P	S	T	W	Y	V	A	R	N	D	C	Q	E	G	H	I	L	K	M	F	P	S	T	W	Y	V		
1 V	-2	-4	-4	-5	-3	-3	-4	-5	-5	1	0	-4	6	-3	-4	-3	2	-4	-3	5	0	0	0	0	0	0	0	0	0	2	5	0	29	0	0	0	15	0	0	50	0.96	0.37
2 L	-5	-6	-7	-7	-5	-6	-6	-7	-6	-2	6	-6	0	3	-6	-6	-4	2	-3	-3	0	0	0	0	0	0	0	0	0	0	86	0	1	10	0	0	0	2	0	0	1.67	0.62
3 S	-3	-4	-1	-4	-3	-4	-4	-4	-5	-3	-5	-4	-3	-6	-4	5	6	-6	-5	-4	1	0	2	0	0	0	0	0	0	1	0	0	1	0	0	50	44	0	0	0	1.48	0.70
4 E	4	-3	-2	4	-3	-1	1	-1	-3	-5	-5	0	-4	-4	0	-1	-1	-6	-5	-3	42	0	1	25	0	1	8	4	0	0	0	7	0	1	4	3	2	0	0	0	0.70	0.41
5 G	2	-1	-1	1	-4	1	3	-1	0	-3	-4	2	-3	-2	-3	0	0	-5	-3	-3	23	1	2	6	0	5	24	4	2	1	0	16	0	2	0	6	4	0	1	0	0.41	0.29
6 E	-5	-4	-3	6	-8	-2	6	-6	-4	-7	-7	-3	-6	-7	-5	-4	-5	-7	-6	-7	0	0	0	40	0	1	59	0	0	0	0	0	0	0	0	0	0	0	0	0	1.97	0.78
7 W	-4	4	-4	-5	-4	-1	-3	-4	-2	-5	-5	6	-3	0	-5	-4	-3	4	-3	-4	0	25	0	0	0	2	1	1	1	0	0	57	0	4	0	0	1	6	1	1	1.39	0.62
8 Q	3	-1	0	1	-2	2	1	-2	0	-4	-4	1	-3	-4	-3	1	2	-4	-4	-2	30	2	3	8	1	12	6	2	2	0	0	8	0	0	0	8	14	0	0	2	0.36	0.29
9 L	2	-2	2	-3	-3	-1	-3	-3	2	1	2	-2	2	-1	-4	-1	1	-4	-2	1	21	2	10	0	0	2	0	0	6	9	22	1	6	1	0	2	9	0	1	7	0.25	0.23
10 V	-3	-6	-7	-7	-4	-6	-6	-7	-7	6	-1	-6	-2	-3	-6	-5	-4	-6	-5	6	1	0	0	0	0	0	0	0	0	46	3	0	0	0	0	0	0	0	0	49	1.62	0.64
11 L	0	2	-1	-3	-2	2	-1	-3	-2	-2	1	3	0	-4	-3	-1	2	-2	-3	-1	8	9	3	0	1	11	1	1	1	2	14	25	3	0	0	3	15	0	0	4	0.31	0.26
12 H	3	-2	1	2	-3	1	-1	1	-1	-3	-4	0	-3	-3	-3	2	1	-5	-4	-3	25	1	6	10	0	5	3	11	2	1	0	7	0	1	0	20	9	0	0	0	0.35	0.26
13 V	-1	-4	-4	-5	1	-4	-4	-5	-1	3	3	-4	2	2	-4	-1	1	-3	-2	2	6	0	0	0	2	0	0	0	1	19	29	0	5	11	0	3	9	0	0	14	0.48	0.28
14 W	-7	-8	-9	-9	-4	-4	-8	-8	-7	-7	-6	-8	-4	3	-9	-8	-7	13	-2	-5	0	0	0	0	0	1	0	0	0	0	0	0	0	9	0	0	0	88	0	1	4.41	1.26
15 A	2	-2	-1	1	-4	-2	0	4	-3	-5	-5	-1	-4	-5	-2	2	-1	-5	-5	-4	20	2	2	9	0	1	7	36	0	0	0	4	0	0	1	15	2	0	0	0	0.60	0.34
16 K	-3	0	-3	-4	-4	-1	-3	-4	0	-4	-3	7	-5	-6	0	-3	-3	-4	-5	-4	1	4	0	0	0	1	0	1	2	1	3	78	0	0	4	1	1	0	0	1	1.62	0.70
17 V	-1	-5	-5	-6	-4	-5	-6	-6	-6	4	1	-5	3	-3	-5	-4	-3	-4	-4	6	4	0	0	0	0	0	0	0	0	25	10	0	7	0	0	0	0	0	0	52	1.16	0.51
18 E	1	-1	0	-1	-2	-1	2	2	-1	-2	-2	0	-2	-1	-2	2	-1	-2	2	-2	13	2	5	2	1	2	15	15	1	3	3	5	1	2	1	17	2	0	8	2	0.17	0.13
19 A	1	-2	-1	-1	-1	0	-1	3	-2	-2	-2	0	-2	-3	2	1	1	-3	-2	0	15	1	2	3	1	3	4	21	1	1	3	6	1	1	11	10	8	0	2	6	0.21	0.12
20 D	-3	-1	5	4	-4	2	0	-4	5	-5	-5	2	-4	-5	-4	-1	-3	-6	-4	-3	1	3	26	26	0	8	4	0	14	0	0	11	0	0	0	4	0	0	0	2	0.84	0.42
21 V	3	-2	-3	-3	3	-2	-1	-2	-2	1	0	-2	-1	-1	-2	0	-1	-3	2	3	24	1	0	0	7	1	5	3	1	6	6	1	0	1	2	6	2	0	8	25	0.27	0.23
22 A	2	-3	-1	4	-3	-2	4	1	-3	-5	-4	-1	-4	-5	0	-1	-2	-5	-4	-4	20	0	2	22	1	0	32	9	0	0	1	2	0	0	3	3	2	0	0	1	0.66	0.37
23 G	1	-2	-1	3	-4	0	4	0	-2	-2	-4	-1	-3	-4	-1	-1	0	-5	-4	-1	16	1	1	20	0	3	31	7	1	2	1	2	0	0	2	2	5	0	0	6	0.47	0.30
24 H	-1	-4	-2	-3	2	-4	-4	-5	3	4	0	-4	-1	1	-5	-4	-2	-2	4	2	5	0	2	1	5	0	0	0	9	30	6	0	1	6	0	0	1	0	15	17	0.59	0.33
25 G	-2	-5	-4	-5	-5	-5	-6	7	-6	-7	-6	-5	-5	-7	-4	-4	-2	-6	-7	-5	2	0	0	0	0	0	0	92	0	0	1	0	0	0	1	0	2	0	0	1	2.22	0.77
26 Q	3	-2	-1	-3	-1	0	-2	3	0	-3	-3	-2	-3	-4	2	0	1	-5	-3	1	30	1	3	0	1	5	1	21	3	0	1	1	0	0	8	4	10	0	0	9	0.39	0.28
27 D	0	-3	-2	2	-5	1	6	-4	-3	-5	-3	-1	-3	-6	-4	-1	-2	-6	-5	-4	10	0	1	9	0	6	61	0	0	0	3	3	0	0	0	3	1	0	0	0	1.13	0.54
28 I	5	-3	-4	-5	-2	-4	-4	-3	-5	1	-1	-4	-3	-4	-4	0	1	-5	-4	2	59	1	0	0	0	0	0	0	0	7	4	0	0	0	0	5	8	0	0	14	0.88	0.47
29 L	-5	-6	-7	-7	-5	-6	-6	-7	-6	-1	7	-6	0	-1	-6	-5	-5	-3	-4	-2	0	0	0	0	0	0	0	0	0	1	93	0	1	2	0	1	0	0	0	1	1.84	0.70
30 I	1	-2	-1	-1	0	-1	2	2	-2	1	-1	-2	-1	-3	-3	1	1	-3	-2	0	12	1	1	2	2	1	19	20	1	9	6	0	1	0	0	10	7	0	1	5	0.19	0.18
31 R	-5	9	-4	-6	-4	-3	-4	-5	-4	-7	-6	-1	-5	-7	-6	-3	-5	-4	-5	-7	0	95	0	0	0	0	0	1	0	0	0	1	0	0	0	2	0	0	0	0	2.63	0.92
32 L	-4	-5	-6	-5	3	-5	-6	-6	-5	-1	5	-5	7	1	-6	-5	0	-5	-4	-2	0	0	0	0	5	0	0	0	0	1	50	0	29	6	0	0	6	0	0	1	1.26	0.56
33 F	-5	-6	-7	-7	-5	-6	-6	-7	-5	-1	4	-6	-1	8	-5	-6	-5	-3	-1	-3	0	0	0	0	0	0	0	0	0	1	41	0	1	55	1	0	0	0	0	1	1.72	0.65
34 K	0	-3	-3	-4	-1	-1	-2	-3	-1	3	1	0	0	-2	-4	-1	2	-3	-3	4	6	1	0	0	1	2	2	1	2	18	11	5	1	1	0	2	17	0	0	29	0.37	0.26
35 S	1	-4	1	-2	0	-3	0	-1	-3	0	-2	-2	-2	-3	-4	2	0	-5	-4	5	9	0	8	1	2	0	7	3	0	2	0	1	0	1	0	15	5	0	0	43	0.47	0.34
36 H	-5	-5	-4	-6	-2	-5	-6	-7	6	-5	-4	-6	-4	5	-6	-5	-5	-2	8	-5	0	0	1	0	1	0	0	0	16	0	1	0	0	25	0	0	0	0	55	0	2.11	0.76
37 P	-5	-6	-6	-6	-7	-6	-5	-6	-6	-7	-6	-4	-7	-8	9	-4	-4	-8	-7	-7	0	0	0	0	0	0	0	0	0	0	1	1	0	0	97	1	0	0	0	0	3.22	1.03
38 E	-2	-3	-3	-2	-5	5	2	-3	-2	-5	-5	-2	-4	-4	-3	2	0	9	-3	-5	2	1	0	2	0	27	11	2	1	0	0	2	0	0	1	15	6	28	0	0	1.27	0.58
39 T	0	-5	-4	-5	-4	-4	-5	-5	-6	-4	-5	-3	-5	-6	-4	0	8	-7	-6	-4	6	0	0	0	0	0	0	0	0	0	0	1	0	0	0	5	86	0	0	0	2.01	0.83
40 L	-4	1	-3	-4	-7	6	-2	-5	-3	-6	-2	6	-3	-6	-5	-3	-3	-6	-5	-6	0	5	0	0	0	32	0	0	0	0	5	57	1	0	0	0	1	0	0	0	1.54	0.69
41 E	-2	5	-3	-1	-5	2	-1	-4	-2	-1	-4	0	-3	-5	-3	-1	5	-5	-4	-2	1	28	0	3	0	8	4	1	1	4	0	5	0	0	1	3	40	0	0	2	0.80	0.46
42 K	-5	-2	-6	-7	-6	-5	-6	-7	2	-5	-1	-1	-4	4	-7	-4	-5	-2	9	-4	0	2	0	0	0	0	0	0	4	0	5	4	0	17	0	1	0	0	66	1	2.06	0.76
43 F	-7	-7	-7	-8	-1	-8	-8	-7	-6	-4	-3	-8	-4	10	-7	-5	-6	-3	-1	-5	0	0	0	0	1	0	0	0	0	0	0	0	0	97	0	1	0	0	0	0	3.09	0.98
44 D	1	-3	-2	2	-4	-2	0	0	-2	-5	-4	-1	-3	-4	5	3	-1	-5	-4	-4	12	1	0	12	0	1	5	6	1	0	1	2	0	1	27	28	3	0	0	0	0.69	0.38
45 R	-3	-1	1	-2	-5	2	-2	-3	9	-6	-5	2	-5	-5	-4	2	-1	-6	-2	-6	1	2	5	2	0	8	1	1	49	0	0	14	0	0	0	13	3	0	1	0	1.39	0.59
46 F	-6	-6	-6	-7	-6	-6	-6	-6	-5	-3	0	-6	-1	9	-7	-6	-6	7	0	-4	0	0	0	0	0	0	0	0	0	1	7	0	1	72	0	0	0	16	1	0	2.36	0.78
47 K	-1	-1	-1	4	-4	-1	-2	4	-3	-5	-5	3	-4	-5	-1	-1	-2	-5	-4	-2	5	2	1	25	0	2	0	34	0	0	0	17	0	0	4	3	1	0	0	3	0.67	0.35
48 H	-2	-3	4	4	-4	-2	-1	0	3	-3	1	-2	-1	-2	-3	-1	-2	-4	-3	-3	2	1	23	26	0	1	2	7	9	1	19	1	1	1	1	2	1	0	0	1	0.53	0.30
49 L	-2	-2	-2	-3	0	-2	-2	-2	-1	2	4	-2	3	0	-1	-2	-1	-2	-2	0	2	2	1	1	2	2	2	2	2	10	46	2	9	3	3	3	3	0	1	3	0.39	0.19
50 K	0	-1	-1	-1	0	-1	1	-1	1	-2	-2	1	-2	-2	0	4	0	-2	1	-2	4	2	2	3	2	2	9	3	4	2	4	7	1	1	5	36	5	0	5	2	0.28	0.17
51 T	0	-1	2	2	-1	-1	0	-1	-1	-2	-1	-1	-1	-1	-1	2	1	-2	-1	-1	6	2	9	12	1	2	6	4	1	2	5	2	1	3	3	23	12	1	2	3	0.17	0.09
52 E	2	-1	0	0	-1	0	1	-1	-1	-1	-1	0	-1	-2	4	0	0	-2	-1	-1	19	2	4	4	1	4	10	3	1	3	6	4	1	2	20	5	4	1	2	4	0.24	0.10
53 A	1	-1	0	1	-1	0	1	0	-1	-1	-1	-1	-1	-2	-1	1	1	-2	-1	-1	15	2	4	11	2	4	11	8	1	2	7	3	1	2	2	12	8	1	1	4	0.09	0.06
54 E	3	-1	-1	1	-2	0	2	-1	-2	0	-1	-1	-1	-2	-1	-1	-1	-2	-2	0	32	2	2	7	1	5	17	3	1	4	7	2	1	1	2	3	3	0	1	6	0.22	0.14
55 M	-1	-2	-1	-3	-2	-1	-2	-2	2	3	1	-2	4	-2	-3	1	0	-3	-2	1	3	2	2	1	1	2	1	2	7	25	8	1	14	1	1	15	4	0	1	8	0.29	0.20
56 K	0	-1	-2	-2	-1	0	1	-3	2	0	-1	1	4	-3	5	-2	-1	-4	-3	-2	9	2	2	1	1	4	9	1	6	5	6	8	13	1	26	2	2	0	0	2	0.44	0.26
57 A	0	1	1	-1	-4	-1	-3	6	1	-5	-5	-1	-4	-5	-4	0	-3	-5	-4	-4	5	7	6	3	0	3	0	60	3	0	0	2	0	0	0	6	1	0	0	1	1.02	0.43
58 S	-3	-4	6	-2	-2	-3	-3	-4	-2	-6	-6	-3	-5	-6	-4	6	-2	-6	-5	-5	1	0	36	1	1	0	0	0	1	0	0	0	0	0	0	58	1	0	0	0	1.54	0.68
59 E	4	-3	-3	-1	-4	-1	1	-2	-1	-4	-3	-2	-4	-4	5	0	-2	-5	-4	-2	37	1	0	3	0	2	11	3	1	0	1	2	0	0	29	6	2	0	0	2	0.78	0.41
60 D	-1	0	0	1	-5	6	-1	-4	1	-5	-4	3	0	-5	1	-2	-3	-5	-4	-4	6	3	3	9	0	37	2	1	4	0	1	22	3	0	8	1	0	0	0	0	0.79	0.44
61 L	-2	-5	-6	-6	-4	-5	-6	-6	-6	2	3	-5	0	-3	-6	-5	-3	-5	-4	6	1	0	0	0	0	0	0	0	0	10	28	0	1	0	0	0	0	0	0	58	1.24	0.54
62 K	1	5	-3	-4	-5	1	-2	-4	0	-4	-1	5	-3	-5	-3	-1	-2	-5	-4	-4	11	28	0	0	0	5	0	0	2	1	6	42	0	0	0	3	1	0	0	1	0.90	0.46
63 K	4	-1	-1	-3	-4	-1	-1	-1	0	-3	-4	4	-2	-5	-3	0	0	-5	-4	-2	42	1	3	1	0	2	3	4	3	1	0	25	1	0	0	7	6	0	0	3	0.59	0.38
64 H	-6	-5	-4	-6	-4	-1	-5	-5	11	-8	-5	-5	-6	-6	-7	-5	-6	-7	-2	-8	0	0	0	0	0	2	0	1	95	0	1	0	0	0	0	0	0	0	1	0	3.68	1.13
65 G	1	-6	-4	-5	-6	-5	-6	7	-6	-7	-7	-4	-6	-7	-6	-2	-5	-6	-7	-7	9	0	0	0	0	0	0	88	0	0	0	1	0	0	0	2	0	0	0	0	2.15	0.76
66 V	1	2	-2	-3	1	1	-1	-1	-1	-2	-3	5	-3	-4	-4	-1	-1	-5	-2	-1	12	8	1	1	3	7	3	3	1	3	1	45	0	0	0	3	3	0	1	4	0.58	0.36
67 T	-3	3	-1	-4	-5	-2	-2	-5	-4	-2	-4	6	-3	-5	-4	-3	3	-6	-5	0	0	12	2	0	0	0	1	0	0	2	1	51	0	0	0	0	22	0	0	7	1.03	0.53
68 V	-3	-6	-6	-7	-4	-6	-6	-7	-7	3	-2	-6	1	-3	-6	-5	-3	-6	-5	7	0	0	0	0	0	0	0	0	0	15	1	0	3	1	0	0	0	0	0	79	1.75	0.69
69 L	1	-5	-5	-6	-3	-4	-5	-4	-5	0	4	-5	7	0	-5	-4	-3	-4	-4	1	13	0	0	0	0	0	0	1	0	3	41	0	29	3	0	0	0	0	0	9	0.98	0.47
70 T	1	-1	2	2	-3	-1	-1	2	1	-3	-3	0	-2	-4	-3	0	3	-4	-2	-3	14	3	10	10	0	1	3	19	4	0	2	6	1	0	0	5	20	0	1	1	0.32	0.23
71 A	5	-3	-3	-4	-4	-2	-3	2	-4	-5	-5	0	-3	-4	-4	3	-2	-5	-5	-3	54	0	0	0	0	1	0	17	0	0	0	6	0	1	0	19	1	0	0	0	0.95	0.49
72 L	-4	-5	-6	-6	-4	-5	-4	-6	-5	3	4	-5	-1	4	-5	-4	-3	-4	-2	3	0	0	0	0	0	0	1	0	0	14	47	0	0	18	0	0	0	0	0	17	0.94	0.42
73 G	0	-3	2	1	-4	-3	-1	5	-2	-4	-3	-2	-3	-5	-4	0	1	-5	-3	-2	7	1	11	8	0	0	3	46	1	0	2	1	1	0	0	5	11	0	1	2	0.69	0.35
74 A	-2	0	2	3	-3	0	4	-1	-1	-3	-1	1	-2	-4	-3	-1	1	-5	-4	-1	1	5	9	19	0	2	26	3	1	0	6	8	1	0	1	2	11	0	0	4	0.40	0.26
75 I	6	-4	-4	-4	-1	-4	-4	1	-5	0	0	-4	-2	-4	-3	-1	-2	-5	-4	0	63	0	0	1	1	0	0	9	0	6	9	0	1	0	0	1	1	0	0	7	0.88	0.47
76 L	0	-5	-5	-6	-2	-5	-5	-5	-5	2	2	-5	0	-3	-5	-3	-2	-5	-3	6	6	0	0	0	1	0	0	0	0	9	20	0	1	0	0	2	1	0	1	59	1.03	0.47
77 K	-1	-1	2	0	-4	1	2	0	0	-5	-5	6	-3	-5	-4	-1	-2	-5	-4	-4	4	1	9	4	0	7	10	7	2	0	0	47	0	0	0	4	2	0	0	0	0.73	0.41
78 K	-1	-2	5	-1	-4	0	-3	-4	7	-6	-4	2	-4	-5	-5	0	-3	-6	-1	-5	5	1	32	3	0	3	0	0	31	0	1	14	0	0	0	7	0	0	2	0	1.13	0.52
79 K	-4	-2	-5	-6	-4	-3	-5	-6	-5	4	4	1	5	-2	-4	-4	-3	-5	-4	0	0	1	0	0	0	1	0	0	0	27	39	11	17	0	1	0	0	0	0	3	0.91	0.44
80 G	-4	-4	0	7	-7	-3	-1	1	2	-7	-7	-4	-6	-6	-5	-2	-4	-7	-3	-6	1	1	4	71	0	0	3	12	5	0	0	0	0	0	0	2	0	0	1	0	1.69	0.66
81 H	-4	-4	6	6	-4	-1	-2	-3	2	-6	-7	-2	-6	-6	-5	-1	-2	-7	-4	-6	0	0	39	44	0	3	1	1	5	0	0	2	0	0	0	3	2	0	0	0	1.46	0.60
82 H	-3	-4	-5	-5	-4	-4	-4	-5	4	4	4	-5	2	-1	0	-3	-3	-5	-3	0	1	0	0	0	0	0	0	1	13	23	46	0	4	1	5	2	1	0	0	5	0.81	0.41
83 E	1	0	-1	0	-3	-1	2	-2	-1	-3	-3	4	-2	-4	2	0	0	-5	-3	-1	13	5	3	5	0	1	12	1	1	0	1	28	1	0	10	5	7	0	0	5	0.34	0.24
84 A	2	-2	1	0	-2	0	-1	4	-2	-4	-4	-1	-3	-5	-3	2	0	-5	-3	-2	20	1	5	5	0	3	4	32	1	0	0	3	0	0	0	16	6	0	1	2	0.49	0.29
85 E	3	-4	-2	-3	0	-3	0	1	-1	0	-2	-3	-3	-2	-3	-1	3	-5	-3	1	31	0	2	0	2	0	6	13	1	5	2	0	0	1	1	2	24	0	1	10	0.40	0.31
86 L	-4	-5	-6	-7	-5	-3	-6	-6	-5	-1	5	-5	0	4	-6	-5	-4	-3	3	-2	0	0	0	0	0	1	0	0	0	2	64	0	1	19	0	0	0	0	11	1	1.27	0.54
87 K	4	-2	-2	-3	-2	-1	-3	-2	-1	-4	-1	2	-3	-2	-4	4	0	-5	-4	-3	34	1	1	0	0	2	0	1	1	0	7	13	0	2	0	33	3	0	0	1	0.60	0.40
88 P	1	-1	0	0	-4	1	1	-3	0	-3	-1	3	-3	-4	4	0	-1	-5	-2	-3	11	1	4	5	0	7	8	1	2	0	7	22	0	0	18	7	3	0	1	1	0.36	0.24
89 L	-5	-6	-7	-7	-5	-6	-6	-7	-6	-1	6	-6	1	-3	-6	-6	-5	-5	-4	1	0	0	0	0	0	0	0	0	0	0	89	0	2	0	0	0	0	0	0	8	1.76	0.69
90 A	2	-2	-3	-4	-4	-2	-4	1	-4	-5	-6	-4	-5	-6	-4	6	-2	-6	-5	-5	16	2	0	0	0	2	0	9	0	1	0	0	0	0	0	71	0	0	0	0	1.37	0.67
91 Q	-3	-1	0	4	-5	2	5	-3	-2	-3	-4	2	-4	-5	-4	-2	0	-6	-5	-1	1	2	4	23	0	7	35	1	0	1	1	13	0	0	0	1	5	0	0	5	0.73	0.40
92 S	0	-3	-5	-4	-4	-2	-4	-5	-5	-1	5	-1	1	-2	-5	0	-1	-5	-3	-1	7	1	0	1	0	1	0	0	0	0	67	3	3	1	0	9	4	0	1	1	0.88	0.46
93 H	-6	-3	-4	-6	-8	-4	-5	-7	11	-8	-5	-5	-6	-6	-5	-6	-6	-7	-3	-8	0	1	0	0	0	0	0	0	97	0	1	0	0	0	1	0	0	0	0	0	3.79	1.15
94 A	6	-5	-4	-5	6	-4	-4	-4	-3	-3	-5	-4	-4	-3	-3	1	-3	-6	-4	-3	67	0	0	0	17	0	0	0	1	1	0	0	0	1	1	10	0	0	0	0	1.42	0.64
95 T	-3	-2	1	2	-3	1	2	-4	4	-2	-1	-2	-3	3	-4	-2	1	-3	4	-2	0	1	6	14	0	6	13	0	9	1	7	1	0	13	0	0	11	0	15	1	0.43	0.30
96 K	-4	0	0	-2	-6	2	-1	-5	-4	-1	-5	7	-1	-6	-4	-3	0	-6	-5	-1	0	3	5	1	0	7	2	0	0	4	0	64	1	0	0	1	6	0	0	4	1.14	0.54
97 H	-5	-5	-5	-6	-5	-5	-5	-6	6	-2	6	-5	-1	-2	-6	-5	-5	-5	-3	-3	0	0	0	0	0	0	0	0	20	1	75	0	1	1	0	0	0	0	0	0	1.59	0.65
98 K	-4	6	-1	-4	-3	-1	-3	-5	7	-6	-4	4	-3	-4	-5	-3	-4	-6	-3	-6	1	42	2	0	1	1	0	0	27	0	2	21	1	0	0	1	0	0	0	0	1.43	0.60
99 I	-3	-6	-6	-6	-4	-6	-6	-6	-6	4	-2	-6	-1	-4	-6	-5	-3	-6	-5	7	1	0	0	0	0	0	0	0	0	18	0	0	1	0	0	0	0	0	0	78	1.74	0.68
100 P	-4	-5	-2	8	-4	-4	1	-4	-3	-7	-7	-4	-6	-7	2	-3	-4	-7	-6	-6	1	0	1	78	0	0	7	1	0	0	0	0	0	0	11	1	1	0	0	0	1.99	0.74
101 I	-5	-4	-6	-5	-3	-5	-5	-6	-6	0	-3	-5	-3	-7	8	-4	-4	-7	-6	-1	0	1	0	0	1	0	0	0	0	5	2	0	1	0	83	1	0	0	0	4	2.47	0.85
102 K	1	-2	0	1	-2	-1	2	-1	-1	-1	-2	1	-1	-4	-2	-1	0	-4	-3	3	14	0	4	9	0	2	18	4	1	2	1	8	2	0	1	3	5	0	0	25	0.23	0.23
103 Y	-5	-4	8	-3	-5	-4	-4	-5	-2	-7	-7	-4	-6	-4	-4	-3	-4	-6	4	-6	0	1	82	0	0	0	0	0	0	0	0	0	0	1	1	1	0	0	13	0	2.27	0.85
104 L	-6	-7	-7	-7	-6	-7	-7	-7	-5	-4	0	-7	-4	9	-6	-6	-5	-3	-1	-5	0	0	0	0	0	0	0	0	0	0	7	0	0	90	0	0	1	0	0	0	2.72	0.89
105 E	-4	4	-3	-4	-6	1	1	-4	-2	-5	-5	6	-3	-6	-1	-3	-3	-6	-5	-4	0	22	0	0	0	6	7	1	0	0	0	56	1	0	4	0	0	0	0	1	1.24	0.56
106 F	-4	-2	-6	-6	-4	-5	-6	-7	-5	4	5	-4	-1	2	-4	-5	-3	-4	-2	-1	0	2	0	0	0	0	0	0	0	20	63	1	0	9	1	0	1	0	1	2	1.11	0.50
107 I	-5	-6	-7	-7	-5	-6	-6	-6	-6	3	6	-6	-1	0	-6	-6	-4	-5	-4	-2	0	0	0	0	0	0	0	0	0	14	80	0	0	3	0	0	0	0	0	0	1.54	0.62
108 S	2	-3	-1	-3	2	-3	-4	4	-4	-2	-5	-3	-3	-3	-4	4	0	-5	-5	-3	16	1	2	0	4	0	0	30	0	3	0	0	0	1	0	36	6	0	0	0	0.76	0.43
109 E	-4	-3	3	3	-6	2	2	1	8	-6	-5	-2	-5	-5	-4	-2	-4	-6	-3	-6	0	0	15	14	0	8	13	9	36	0	0	1	0	0	0	2	0	0	0	0	1.13	0.52
110 A	-1	-5	2	-5	9	-4	-5	-5	-4	2	-3	-5	1	-4	-6	-2	-2	-6	-5	3	4	0	10	0	42	0	0	0	0	12	1	0	4	0	0	2	2	0	0	22	1.37	0.56
111 I	-3	-5	-6	-6	-4	-5	-6	-6	-6	6	4	-5	2	2	-5	-5	-4	-4	-3	0	1	0	0	0	0	0	0	0	0	41	41	0	4	10	0	0	0	0	0	2	1.14	0.50
112 I	-2	-4	-4	-4	-3	1	-3	-4	-1	3	4	-4	1	-2	-4	-2	0	-4	-3	2	1	0	0	0	0	7	1	1	2	17	41	0	3	0	0	2	7	0	0	16	0.56	0.32
113 H	-3	-4	-4	-4	0	-2	1	-5	-1	3	0	0	-2	-4	-5	-3	-2	-5	-4	5	1	0	0	0	2	2	9	1	2	19	4	8	0	0	0	1	1	0	0	50	0.78	0.42
114 V	-1	-5	-5	-6	2	-5	-4	-6	-4	0	-2	-5	-2	-3	-5	-2	3	-6	-4	6	3	0	0	0	4	0	1	0	0	2	1	0	1	1	0	2	20	0	0	65	1.25	0.58
115 L	-4	-5	-6	-6	-3	-5	-6	-6	-6	2	5	-5	4	0	-4	-5	-3	-5	-4	2	1	0	0	0	0	0	0	0	0	11	61	0	11	2	0	0	0	0	0	12	1.12	0.49
116 H	6	-5	-4	-5	-2	-2	-2	0	-2	-5	-5	-3	-4	-5	-4	0	-3	-6	-5	-2	78	0	0	0	0	2	2	6	1	0	0	1	0	0	0	7	1	0	0	2	1.38	0.65
117 S	2	1	-1	-2	1	-1	2	-2	0	0	-2	-1	2	-3	-2	1	1	-4	-3	-1	19	8	3	0	3	0	18	3	3	7	2	1	7	0	1	12	7	0	0	4	0.20	0.22
118 R	-3	1	0	-2	-4	-1	0	-5	8	-5	-3	3	2	-3	-4	-3	-3	-5	1	-4	2	6	4	2	0	2	4	0	44	0	3	20	5	1	0	0	1	0	4	1	1.18	0.54

119	H	-1	-4	-3	-6	-2	-4	-5	-6	5	-3	2	-5	2	7	-6	-4	-4	-3	3	-3	5	1	1	0	1	1	0	0	13	0	17	0	4	46	0	1	0	0	10	0	1.21	0.53
120	P	-1	-3	-4	-4	-6	0	-4	4	-2	-6	-6	-3	-4	-7	7	-1	-3	-6	-6	-6	5	1	0	0	0	4	0	34	1	0	0	1	0	0	46	5	1	0	0	0	1.44	0.60
121	G	2	0	1	1	-3	-1	1	2	-1	-4	-2	3	-2	-4	-2	1	-1	-5	-4	-3	16	3	7	8	0	2	7	13	1	0	4	22	1	0	1	10	3	0	0	1	0.28	0.22
122	D	-1	-4	-1	6	-2	-1	4	-3	-2	-4	-3	-3	-5	-3	-4	-2	-3	-6	-4	-1	5	0	1	50	1	1	26	1	1	1	2	0	0	1	1	2	1	0	0	4	1.08	0.52
123	F	-5	-6	-6	-6	-3	-6	-6	-6	-4	-2	0	-6	-1	9	-5	-5	-5	-3	2	-2	0	0	0	0	1	0	0	0	0	1	8	0	1	79	0	1	0	0	4	2	2.17	0.73
124	G	-3	-4	0	-2	-4	-4	-4	0	-2	-4	-4	-3	-4	-5	-1	1	7	-5	-5	-3	1	1	3	2	0	0	0	8	1	0	1	1	0	0	4	8	69	0	0	0	1.30	0.60
125	A	3	-5	-5	-3	-4	-4	-4	-2	-6	-5	-5	-4	-4	-5	8	-2	-3	-5	-4	-1	22	0	0	1	0	1	0	2	0	0	1	0	0	0	63	2	1	0	0	5	1.78	0.70
126	D	2	-3	-2	3	-3	2	5	-3	-2	-5	-5	-2	-3	-6	-2	-1	-2	-6	-5	-2	17	0	1	13	0	8	47	2	0	0	0	1	1	0	2	3	2	0	0	2	0.86	0.46
127	A	2	-5	-5	-5	3	-4	-4	-1	-4	0	0	-4	3	-2	-4	-2	0	-3	-3	5	20	0	0	0	6	0	0	6	0	3	5	0	9	1	0	2	5	0	0	40	0.64	0.39
128	Q	-3	-2	-3	-4	-5	7	-3	-5	9	-5	-5	-3	-5	-6	-6	-4	-5	-6	-3	-6	2	1	0	0	0	51	0	1	42	1	1	1	0	0	0	0	0	0	0	0	2.23	0.88
129	G	5	-1	-4	-3	-3	-1	0	0	-3	-2	-1	-3	-1	-3	-4	-1	-2	-5	-4	2	54	3	0	1	0	3	6	5	0	0	5	1	1	1	0	4	2	0	0	14	0.64	0.41
130	A	6	-5	-4	-4	-3	-4	-4	-3	-5	-4	-5	-4	-4	-6	-3	4	-1	-6	-5	-2	65	0	0	0	0	0	0	1	0	0	0	0	0	0	1	27	3	0	0	2	1.34	0.65
131	M	-5	-5	-7	-7	-2	-5	-6	-7	-5	-2	2	-6	5	4	-7	-6	-5	10	2	0	0	1	0	0	1	0	0	0	0	1	20	0	13	16	0	0	0	36	5	8	1.70	0.64
132	N	-2	1	-1	6	-6	5	0	-4	-2	-5	-6	0	-5	-6	-4	-1	0	-6	-4	-5	2	7	1	44	0	27	4	0	1	0	0	3	0	0	0	4	5	0	0	0	1.13	0.54
133	K	-5	-1	0	-5	-7	-2	-3	-5	-3	-7	-7	8	-6	-7	-5	-4	-4	-7	-6	-7	0	2	3	0	0	1	0	0	0	0	0	93	0	0	0	1	0	0	0	0	2.33	0.89
134	A	-1	-6	-6	-7	-5	-5	-6	-6	-4	-1	2	-5	1	7	-6	-5	-4	-3	0	2	7	0	0	0	0	0	0	0	0	1	20	0	3	53	0	0	0	0	2	13	1.31	0.55
135	L	-3	-2	-5	-6	-3	-5	-5	-6	-5	-1	5	-4	4	2	-5	-4	-1	-4	-3	2	1	2	0	0	0	0	0	0	0	1	58	1	11	7	0	0	3	0	0	16	0.97	0.47
136	E	4	-1	0	-1	2	0	0	1	-2	-4	-4	-2	-2	-4	-3	3	1	-5	-3	-2	33	3	5	2	5	4	7	8	1	0	0	1	1	0	0	21	7	0	1	1	0.43	0.31
137	L	3	-2	1	-3	-1	-1	-2	1	0	0	0	-2	-1	-3	-3	0	-1	-4	-3	2	25	2	7	1	1	2	2	13	3	4	12	1	1	0	0	4	3	0	0	19	0.21	0.21
138	F	-3	-5	-6	-7	-4	-6	-6	-6	-6	2	1	-6	0	2	-6	-5	-4	-5	-4	6	1	0	0	0	0	0	0	0	0	11	12	0	2	8	0	0	0	0	0	66	1.32	0.57
139	R	4	0	-3	-4	2	-3	-3	0	-3	1	-3	-3	-3	-3	-4	2	-1	-4	2	1	38	6	1	0	4	0	0	5	0	8	0	0	0	1	0	17	2	0	7	10	0.47	0.35
140	K	1	-2	3	0	0	-2	-1	-2	3	-2	0	-1	-1	-3	-3	2	2	3	-2	-2	14	2	15	4	2	0	2	1	8	1	11	2	1	1	0	18	12	4	1	2	0.26	0.24
141	D	5	-4	-3	2	-2	-2	-3	-2	3	-2	-4	-4	-2	-4	-4	-1	-2	-6	-4	3	47	0	0	12	0	1	1	1	7	1	0	0	0	0	0	3	1	0	0	23	0.76	0.46
142	I	-4	-6	-7	-7	-5	-5	-6	-7	-6	2	6	-4	3	-3	-6	-6	-4	-5	-4	1	0	0	0	0	0	0	0	0	0	7	77	1	7	0	0	0	0	0	0	7	1.49	0.61
143	A	4	-3	-2	-1	-2	-2	-1	0	-4	-4	-4	-2	-3	-3	-3	2	4	-5	-4	-2	34	1	1	3	1	1	4	8	0	0	0	1	0	1	0	16	26	0	0	2	0.60	0.39
144	A	2	2	-1	0	-4	-1	3	-1	5	-5	-4	0	-4	-5	-3	2	-2	-5	-1	-3	17	10	1	4	0	1	24	3	13	0	1	4	0	0	1	16	1	0	2	1	0.49	0.33
145	K	0	2	-1	-3	-6	2	0	-3	-2	-4	-4	6	-3	-6	-4	-3	-2	-6	-2	-2	6	7	2	1	0	10	4	2	1	1	1	57	0	0	0	1	2	0	2	4	0.99	0.50
146	Y	-7	-7	-7	-8	-7	-6	-7	-8	-2	-5	-6	-7	-6	-1	-8	-6	-6	-2	10	-6	0	0	0	0	0	0	0	0	0	0	0	0	0	0	0	0	0	0	99	0	3.55	1.13
147	K	-3	7	-3	-4	-6	-1	-1	-5	-3	-6	-5	4	-4	-6	-5	-3	-2	-6	-5	-5	1	64	0	0	0	0	2	0	0	0	0	28	0	0	0	1	3	0	0	0	1.63	0.57
148	E	-3	-2	-2	1	-5	0	7	-4	-2	-5	-4	0	-4	-5	-3	-2	-2	-5	-4	-3	0	0	0	5	0	0	91	0	0	0	1	2	0	0	0	0	0	0	0	1	1.37	0.31
149	L	0	-3	-4	-4	-2	-3	-2	-2	-3	1	3	-3	0	2	-3	-2	-2	-3	-1	3	11	0	0	0	0	0	3	2	1	5	41	0	0	9	0	0	0	0	0	28	0.34	0.12
150	G	-1	-4	0	-3	-4	-3	-4	7	-4	-6	-5	-3	-4	-5	-4	-2	-3	-4	-5	-5	0	0	3	0	0	0	0	97	0	0	0	0	0	0	0	0	0	0	0	0	1.72	0.29
151	Y	-4	-5	-5	-6	-4	-4	-5	-5	-3	-3	-2	-5	-2	7	-6	-4	-4	10	2	-3	0	0	0	0	0	0	0	0	0	0	0	0	0	53	0	0	0	43	3	0	2.07	0.33
152	Q	2	-1	-1	-1	-2	4	0	-2	1	-3	-2	0	-2	-3	-2	1	3	-3	-2	-2	21	0	0	2	0	34	3	0	3	0	1	2	0	0	0	11	25	0	0	0	0.41	0.14
153	G	-1	-3	2	-2	-3	-2	-3	6	-2	-5	-5	-2	-4	-4	-3	-1	-2	-4	-4	-4	0	0	11	0	0	0	0	89	0	0	0	0	0	0	0	0	0	0	0	0	1.23	0.14

	K	Lambda
Standard Ungapped	0.1344	0.3180
Standard Gapped	0.0535	0.2670
PSI Ungapped	0.1747	0.3173
PSI Gapped	0.0535	0.2670

PSSM #4

Query protein:
Myoglobin from *Physeter macrocephalus*
(PDB 1MBN, chain A)

Target dataset:
UniRef25-2015, 5.02 million proteins
(full size)

PSSM generator:
PSI-BLAST

(see the next page)

Last position-specific scoring matrix computed, weighted observed percentages rounded down, information per position, and relative weight of gapless real matches to pseudocounts

	A	R	N	D	C	Q	E	G	H	I	L	K	M	F	P	S	T	W	Y	V	A	R	N	D	C	Q	E	G	H	I	L	K	M	F	P	S	T	W	Y	V		
1 V	-4	-5	-5	-6	-4	-4	-5	-6	-5	4	3	-5	7	-2	-5	-4	-3	-4	-4	2	0	0	0	0	0	0	0	0	0	21	32	0	34	0	0	0	0	0	0	13	1.16	0.48
2 L	-5	-7	-8	-8	-6	-7	-8	-8	-7	0	6	-7	5	2	-4	-7	-6	-6	-5	-1	1	0	0	0	0	0	0	0	0	5	69	0	13	7	1	0	0	0	0	4	1.71	0.79
3 S	-2	-3	1	-1	-6	-4	-4	-5	-6	-7	-7	-4	-6	-7	-6	5	6	-8	-7	-6	2	1	6	4	0	1	1	0	0	0	0	1	0	0	0	36	47	0	0	0	1.56	0.88
4 E	2	-1	-1	2	-5	1	3	-2	-1	-2	-2	0	0	-5	2	0	0	-3	-3	-2	16	3	2	12	0	6	17	3	2	3	4	6	2	0	11	8	4	0	1	2	0.29	0.27
5 G	2	2	-1	2	-5	1	3	-2	-1	-2	-4	2	-3	-2	-3	-1	-1	-3	-3	-3	16	12	3	11	0	5	23	2	2	2	1	11	0	2	1	5	3	0	1	1	0.41	0.33
6 E	-4	-3	-3	4	-4	6	5	-7	0	-6	-7	-3	-4	-8	-5	-3	-2	-8	-5	-4	1	1	1	21	0	29	36	0	2	0	0	1	0	0	0	1	3	0	0	1	1.44	0.81
7 W	-1	2	-5	-5	-2	0	-3	-5	-3	4	-2	4	0	-3	-6	-3	-2	1	-3	2	4	12	0	0	1	5	1	0	1	22	4	30	2	1	0	1	2	2	1	12	0.70	0.47
8 Q	2	1	0	2	-3	1	2	-2	-1	-2	-3	2	-2	-1	-4	0	-1	-5	-1	-2	18	7	4	9	1	6	15	2	2	2	2	14	1	2	0	5	4	0	3	2	0.27	0.26
9 L	1	-1	-1	-3	-1	-2	-1	-4	-2	3	3	-2	0	-2	-5	-2	0	-5	-2	1	13	3	3	1	1	1	4	1	1	14	35	2	2	1	0	2	6	0	1	8	0.41	0.32
10 V	-3	-7	-8	-8	-6	-7	-8	-8	-8	6	3	-7	1	-2	-7	-7	-3	-7	-6	5	2	0	0	0	0	0	0	0	0	36	23	0	3	1	0	0	1	0	0	34	1.49	0.73
11 L	-2	4	-2	-4	-4	4	0	-5	-1	-2	-1	4	-2	-4	-6	-2	-1	-7	-4	-1	3	25	2	1	0	19	5	1	2	2	6	23	1	1	0	2	4	0	0	5	0.77	0.53
12 H	0	1	2	1	-3	2	2	-2	0	-4	-4	2	-3	-3	-3	2	0	-6	-5	-4	9	6	10	9	1	7	12	2	2	0	1	13	1	1	1	17	6	0	0	0	0.38	0.33
13 V	-1	-6	-1	-1	-2	-1	-4	-3	0	-1	-3	-3	-6	-4	-6	5	4	-7	-4	-1	4	0	3	3	1	3	1	2	2	4	2	1	0	1	0	43	27	0	0	4	0.99	0.68
14 W	-3	-10	-10	-11	-3	-9	-10	-6	-9	-3	-4	-10	-3	4	-6	-7	-6	12	-1	-2	3	0	0	0	1	0	0	1	0	1	2	0	1	13	1	0	1	73	2	3	3.76	1.55
15 A	2	0	1	1	-2	1	1	-1	-1	-3	-3	2	-2	-4	1	0	-1	-5	-3	-2	17	4	6	8	1	5	11	4	1	1	3	16	1	1	7	7	4	0	0	3	0.24	0.24
16 K	0	0	-1	-1	-3	1	0	-1	0	1	1	2	0	-1	-2	0	0	-2	-1	0	9	5	2	3	0	6	5	5	2	8	14	16	3	2	2	5	5	1	2	7	0.07	0.11
17 V	-1	-4	-7	-5	-5	-4	-5	-7	-6	5	3	-6	2	0	-4	-4	-3	-6	-3	4	5	1	0	1	0	1	1	0	0	26	25	0	4	3	1	1	1	0	1	29	0.99	0.54
18 E	1	1	-1	-2	-2	1	1	-1	-2	-3	-1	3	0	0	-3	1	-1	1	1	-2	11	6	3	2	1	7	8	4	1	1	5	21	3	4	1	9	4	2	6	3	0.20	0.22
19 A	1	1	0	0	-1	1	1	0	-2	-2	-3	2	-2	-4	3	0	-1	-5	-4	-2	11	9	3	5	1	7	11	8	1	3	3	11	1	1	13	8	2	0	0	3	0.24	0.23
20 D	-2	1	4	5	-4	0	0	-2	3	-2	-4	0	-3	-1	-5	-1	-1	-6	-2	-2	2	6	20	27	0	4	4	3	7	2	2	6	1	3	0	4	4	0	1	3	0.61	0.42
21 V	1	0	-2	-2	-2	0	-1	-1	-2	1	1	0	1	-1	2	0	-1	1	0	-1	13	6	2	2	1	3	4	6	1	10	13	6	4	2	12	6	2	2	4	4	0.10	0.13
22 A	0	0	1	2	-4	1	2	-1	1	-1	-2	0	-1	-3	-1	0	1	-4	-1	0	9	5	5	12	0	5	17	4	3	3	4	6	2	1	3	6	8	0	2	6	0.17	0.17
23 G	1	0	0	2	-4	1	3	1	-1	-2	-3	1	-4	-2	-2	0	1	-1	-2	-2	12	4	4	13	0	6	17	9	1	2	3	9	0	1	1	5	8	1	1	2	0.24	0.23
24 H	0	-3	2	-2	-1	-3	-3	-4	3	3	0	-3	0	2	-3	0	0	0	1	2	8	1	10	1	1	0	1	0	7	17	10	1	2	10	1	6	7	1	4	12	0.27	0.24
25 G	2	-4	-4	-5	1	-3	-3	6	-3	-5	-4	-4	-3	-3	-3	-1	0	-3	-5	-3	16	1	1	0	2	1	2	55	1	1	2	1	1	2	2	5	6	0	0	2	1.12	0.63
26 Q	0	1	0	-1	-1	0	0	-2	-1	0	0	1	1	-2	-1	0	1	-2	0	1	9	8	4	3	1	5	6	2	2	5	9	9	5	1	3	5	8	0	3	12	0.06	0.09
27 D	0	1	1	1	-1	0	2	-2	1	0	0	1	-1	-1	-2	0	0	-2	-2	0	9	8	6	8	1	4	14	3	3	6	9	7	2	2	1	5	5	0	1	6	0.08	0.10
28 I	-1	-6	-3	-7	1	-6	-6	-6	-6	4	1	-4	4	5	-6	-4	0	-1	-1	2	6	0	1	0	3	0	0	0	0	21	11	1	9	25	0	1	6	1	1	14	0.85	0.48
29 L	-5	-7	-7	-8	-7	-7	-5	-8	-5	-2	2	-7	1	7	-8	-4	-5	0	6	-2	1	0	0	0	0	0	1	0	0	2	21	0	3	40	0	2	1	1	26	2	1.67	0.79
30 I	0	0	-1	0	-2	0	1	-1	0	1	1	0	1	-1	-2	0	0	-3	0	0	7	4	3	4	1	5	9	5	2	12	13	8	4	2	0	6	6	0	4	5	0.04	0.08
31 R	-2	5	0	-2	-3	0	-1	-1	0	-3	-2	3	0	-3	-5	0	-1	0	-2	-3	3	32	5	2	0	4	4	4	2	1	5	16	3	1	0	9	4	1	1	1	0.51	0.38
32 L	-2	-6	-7	-7	-2	-6	-4	-7	-6	1	5	-6	5	4	-6	-6	-4	0	1	-1	3	0	0	0	1	0	1	0	0	8	49	0	13	15	0	0	1	1	4	3	1.16	0.61
33 F	-4	-7	-7	-8	-2	-7	-5	-7	-2	-3	1	-5	0	9	-8	-4	-4	-4	1	-4	2	0	0	0	1	0	1	0	1	1	14	1	2	69	0	2	1	0	3	1	2.16	0.98
34 K	1	0	-1	1	-3	2	4	-3	0	-3	-3	2	-2	-2	-4	0	1	-5	-3	-2	11	4	3	7	1	7	25	1	2	2	3	11	1	2	0	7	10	0	1	3	0.34	0.32
35 S	0	1	0	1	-4	1	1	-3	0	0	0	2	1	-4	-3	0	0	-1	-2	-1	10	7	4	10	0	5	11	1	3	7	8	13	4	0	0	5	7	1	1	4	0.16	0.18
36 H	0	-2	3	2	-1	-2	-1	-4	6	-3	-3	-2	-2	1	-5	-1	-3	-4	5	-3	9	2	14	9	1	1	4	1	21	1	2	2	1	5	0	4	1	0	19	2	0.76	0.50
37 P	-3	-4	-3	-3	-7	-3	-3	-3	-1	-7	-5	-3	-7	-7	8	-3	-2	-1	-7	-5	3	1	2	1	0	1	2	2	2	0	2	2	0	0	74	3	3	1	0	1	2.19	1.08
38 E	-1	-1	0	3	-3	1	5	-2	1	-4	-3	-2	-5	-1	-3	0	-1	0	-2	-3	6	2	4	17	1	5	32	4	3	1	3	2	0	3	1	6	4	1	1	1	0.54	0.41
39 T	0	-2	0	-2	0	-3	-2	-3	-1	2	2	-3	1	1	-4	-1	2	0	2	1	7	2	4	2	2	0	3	2	2	10	19	1	3	5	0	5	13	1	8	12	0.21	0.21
40 L	-2	3	-3	-3	-2	4	-1	-3	-2	-1	0	4	0	-2	-5	-3	-2	-1	-2	-2	3	15	1	1	1	18	4	2	1	3	9	29	2	2	0	2	2	1	1	3	0.65	0.47
41 E	1	0	1	1	-4	2	1	-2	1	-2	-2	1	-1	-2	0	0	0	-4	0	-1	12	4	7	9	0	8	10	3	3	3	4	10	1	1	5	7	6	0	3	4	0.14	0.16
42 K	-2	-2	-1	-3	-2	-2	-3	-5	0	1	3	1	2	2	-2	-3	-2	0	4	0	4	2	3	1	1	2	2	0	2	8	24	8	6	8	2	2	2	1	16	6	0.39	0.29
43 F	-2	-7	-7	-2	-7	-7	-7	-7	1	-1	-2	-7	1	8	-8	-2	-7	4	1	-2	3	0	0	3	0	0	0	0	3	3	4	0	3	63	0	4	0	4	4	3	2.02	0.88
44 D	-1	-1	1	-1	0	0	0	0	0	0	-2	-3	0	0	-1	5	-1	0	-5	-1	0	5	3	6	3	2	4	7	8	3	3	3	7	2	3	24	5	5	0	2	7	0.31	0.23
45 R	-1	1	0	0	0	1	-1	-1	2	-1	0	2	0	0	0	-1	-1	1	0	-2	6	6	5	7	2	8	3	4	7	4	10	12	3	3	5	4	3	2	4	3	0.08	0.09
46 F	-1	-3	-2	-2	-2	-3	-2	-3	-2	0	1	-2	0	7	-2	-1	-2	-1	0	-1	5	1	2	3	1	1	3	3	1	5	13	3	2	39	2	5	3	1	2	5	0.75	0.35
47 K	0	3	-1	-1	-3	0	0	0	1	-2	-3	3	0	-2	-1	0	0	-3	-1	-1	8	16	2	4	0	3	5	7	3	2	3	19	2	2	3	8	6	0	2	4	0.24	0.18
48 H	-1	-1	3	3	-2	0	1	2	3	-2	-4	0	-2	-2	-2	0	-1	-4	-2	-3	5	3	12	17	1	4	7	13	8	2	2	5	1	2	2	8	3	0	1	2	0.33	0.23
49 L	-2	-1	-1	0	0	0	-1	-1	-1	2	1	-1	3	0	-2	-1	-1	-3	0	2	3	3	4	6	2	4	3	5	1	11	16	4	8	3	2	4	4	0	3	14	0.12	0.11
50 K	-1	-1	-1	2	-1	1	1	-3	-1	-2	-2	2	-1	-1	4	0	0	-1	-2	-1	5	3	3	11	1	5	8	3	1	2	4	10	2	2	18	8	6	1	2	4	0.27	0.19
51 T	-1	-1	2	2	-2	-1	-1	-1	0	0	-2	-2	0	-1	0	2	2	-1	-1	1	5	3	9	11	1	2	4	6	2	5	4	2	2	2	4	14	11	1	2	10	0.13	0.13
52 E	0	0	1	1	-2	1	2	-1	0	-2	0	1	-1	-1	0	0	-1	-2	-1	-2	9	5	7	7	1	7	12	5	3	3	11	8	2	2	4	8	3	1	2	3	0.08	0.10
53 A	0	-1	0	3	-2	1	2	1	-1	-2	-2	0	-1	-2	-1	0	-1	-4	-2	-1	9	2	4	15	1	6	16	9	1	3	4	6	1	1	3	7	4	0	2	5	0.19	0.17
54 E	-1	0	1	3	-2	2	3	-2	0	-2	-2	0	-1	-2	-1	0	-1	-4	-2	-2	5	4	7	17	1	8	21	3	2	3	4	4	1	2	3	6	4	0	1	3	0.34	0.25
55 M	-1	-3	-1	-2	-2	0	-3	-2	0	2	4	-1	3	-1	-2	-3	-2	0	-2	1	5	2	3	2	1	5	2	3	2	11	34	4	8	2	3	2	2	1	1	7	0.42	0.25
56 K	1	2	-1	-2	-2	0	0	-1	-1	0	-1	3	1	0	0	0	-1	1	-1	-1	10	12	3	1	1	4	6	4	1	5	7	17	3	4	5	6	3	2	2	5	0.14	0.14
57 A	0	0	2	1	-2	1	1	0	0	-2	-2	1	1	-1	-2	1	0	1	0	-2	6	6	9	8	1	6	7	8	2	2	4	7	4	2	2	12	7	2	3	3	0.11	0.12
58 S	-2	-2	4	2	-2	0	-1	-2	2	-3	-3	-2	-2	-3	-3	4	0	-5	-2	-3	4	3	18	11	1	4	4	3	4	2	3	2	1	1	2	27	6	0	1	3	0.54	0.40
59 E	0	-1	-1	0	-4	-1	1	-2	1	-2	-3	1	-1	-2	4	0	0	0	0	-1	8	4	2	5	0	3	11	3	3	2	2	8	2	2	21	7	7	2	3	4	0.31	0.27
60 D	0	2	1	1	0	2	1	-2	0	-1	-1	1	-1	-1	-2	0	-1	0	-1	0	8	11	5	9	2	9	9	2	2	3	5	8	2	1	2	7	3	1	2	7	0.13	0.14
61 L	-2	-3	-3	-5	-2	-2	-3	-3	-5	1	3	-5	3	5	-3	-3	-2	0	-1	1	3	2	2	0	1	2	2	2	0	9	27	0	8	26	2	2	2	1	1	8	0.72	0.42
62 K	0	3	-2	-2	-1	2	1	-2	1	-1	-1	2	-1	-2	-2	-1	-1	-1	-2	-1	9	19	2	2	1	8	8	3	3	3	7	14	2	2	1	5	4	1	1	5	0.23	0.21
63 K	2	1	0	0	-2	1	1	-1	0	-2	-1	1	-1	-1	-2	0	0	2	-1	-2	21	7	5	5	1	5	8	5	2	2	5	8	2	3	1	6	5	3	2	2	0.12	0.15
64 H	-2	-3	-5	-5	-6	4	-2	-3	9	-3	-1	-1	-1	-2	-3	-3	-5	-6	0	-4	5	2	0	0	0	16	2	3	49	2	6	4	1	2	1	2	0	0	4	2	1.73	0.87
65 G	4	-3	-3	-3	2	-2	-3	2	-5	-1	-2	-3	1	0	-3	0	-2	-1	-2	1	36	2	1	1	4	2	2	15	0	3	4	2	4	4	1	7	3	1	1	8	0.48	0.37
66 V	0	0	0	0	1	0	0	0	0	0	0	0	1	-1	-2	1	0	-3	0	0	8	6	5	3	3	4	5	8	3	5	7	7	5	3	0	10	7	0	3	6	0.03	0.06
67 T	0	0	0	0	2	0	0	-1	1	0	-1	0	1	0	0	0	0	-2	1	0	5	5	5	5	5	5	5	5	5	5	5	5	5	5	5	5	5	0	5	5	0.03	0.05
68 V	-1	-5	-5	-3	-1	-5	-3	-4	-5	3	1	-3	2	3	-5	-3	-2	-4	1	4	5	0	0	2	1	0	2	2	0	15	14	2	6	12	0	2	2	0	4	31	0.65	0.38
69 L	-1	-3	-2	-5	0	-2	-2	-2	-1	2	2	-3	7	2	-5	-2	-2	0	-1	1	5	2	2	0	2	2	3	3	2	13	15	2	24	9	0	3	3	2	2	9	0.62	0.39
70 T	0	0	1	0	0	0	0	0	0	0	-1	0	0	0	-1	-1	0	0	0	1	-1	6	4	9	6	2	4	6	9	3	2	8	7	3	2	2	8	6	1	7	5	0.04	0.07
71 A	3	-2	-2	-2	-1	-1	-1	0	-1	0	0	-2	2	0	-1	0	2	-4	1	1	23	2	2	2	1	2	3	6	2	4	7	2	6	4	3	7	12	0	4	9	0.17	0.19
72 L	-1	-2	-6	-6	0	-5	-2	-6	0	4	4	-5	2	1	-6	-3	-2	-5	1	2	5	2	0	0	2	0	3	0	2	19	34	0	4	5	0	2	2	0	4	13	0.67	0.40
73 G	0	-1	2	3	0	-1	1	0	0	0	-2	-1	1	-4	-3	1	0	-4	-2	-1	9	2	12	15	2	2	8	8	2	5	3	3	4	0	0	10	6	0	1	5	0.17	0.20
74 A	0	0	2	1	-1	1	1	-2	0	-1	-1	2	-1	0	-3	0	1	-1	0	-2	7	4	9	8	1	7	8	2	2	4	6	11	2	4	0	8	10	1	3	2	0.12	0.15
75 I	2	-4	-1	-4	2	-2	-4	-1	-1	2	1	-3	2	1	-4	-1	-1	0	0	2	18	0	4	0	4	2	0	5	2	12	15	2	5	5	0	3	4	2	3	15	0.28	0.23
76 L	-2	-6	-7	-7	-2	-6	-6	-5	-3	5	2	-6	2	1	-4	-4	-2	-6	-3	5	4	0	0	0	1	0	0	1	1	28	19	0	4	2	1	2	2	0	1	33	1.07	0.57
77 K	-1	0	2	4	-1	1	2	-1	1	-4	-3	1	-2	-3	-3	1	-1	-2	-2	-3	6	4	8	23	1	7	15	4	3	1	3	7	1	1	1	8	4	1	1	2	0.39	0.31
78 K	-1	0	5	2	-2	1	-1	-1	4	-4	-2	0	-3	-2	-2	1	-1	-6	0	-3	5	6	27	9	1	5	4	4	9	1	4	5	1	1	3	9	3	0	3	1	0.47	0.36
79 K	-2	-3	-5	-5	-1	-4	-4	-5	-6	3	5	-2	2	0	0	-3	-2	-2	-3	0	3	2	1	1	1	1	1	1	0	13	49	2	5	4	6	2	3	0	1	6	0.79	0.49
80 G	-3	-1	1	6	-7	-1	3	1	2	-7	-4	-3	-6	-3	-3	0	-2	-7	-3	-5	2	4	6	37	0	2	17	11	4	0	2	2	0	1	2	6	2	0	1	1	0.86	0.56
81 H	-1	0	4	5	-3	0	0	-2	1	-4	-4	1	-2	-3	-5	0	-1	-6	-2	-2	5	4	17	27	1	3	7	3	3	1	2	10	1	1	0	7	4	0	1	3	0.56	0.40
82 H	-2	-2	-1	-1	-2	-2	-1	-3	0	1	3	-2	3	-1	3	-2	-2	-2	-2	-1	4	2	3	4	1	2	4	2	2	7	30	2	8	2	16	3	2	1	1	4	0.40	0.31
83 E	-1	0	0	2	-2	0	4	-2	1	-3	-3	2	-1	-4	-2	0	-1	-6	-4	-1	5	5	4	13	1	4	26	3	4	2	2	11	2	1	2	7	4	0	1	5	0.39	0.33
84 A	2	-1	-2	0	0	0	2	-1	-1	-2	-2	1	-2	-3	2	1	1	-5	-2	-1	15	3	2	5	2	4	12	5	1	2	3	8	1	1	11	10	8	0	1	4	0.19	0.21
85 E	0	-1	-2	-1	-2	0	0	-3	0	2	1	-1	2	1	-2	-1	0	-3	1	1	7	3	2	3	1	5	7	2	3	11	15	4	5	7	1	3	5	0	5	12	0.11	0.13
86 L	-2	-2	-5	-7	1	-4	-5	-5	-7	1	5	-4	2	2	-7	-4	-3	0	-4	2	3	2	1	0	3	1	1	1	0	7	50	1	4	7	0	1	2	1	0	14	0.95	0.55
87 K	0	1	0	1	-1	1	2	-2	1	-1	-2	2	-1	-2	-1	0	0	-2	-2	-1	8	9	5	7	1	7	12	3	4	3	4	15	1	2	3	7	5	1	1	4	0.15	0.16
88 P	0	1	0	1	-3	0	2	-1	2	-1	-1	2	-1	-2	0	0	-1	-1	-1	-1	8	9	3	8	0	4	12	4	7	4	6	11	2	1	5	5	5	1	2	4	0.12	0.13
89 L	-3	-6	-7	-7	-6	-3	-5	-7	-7	3	5	-5	3	-1	-5	-5	-2	-6	-3	1	3	0	0	0	0	1	1	0	0	17	55	1	7	3	1	1	2	0	1	8	1.23	0.66
90 A	2	-2	-4	-4	-2	-1	-4	5	0	0	-2	-2	-3	-4	-5	0	-2	-2	-3	0	18	2	1	1	1	2	1	43	2	5	5	2	1	1	0	6	2	1	1	6	0.75	0.49
91 Q	0	3	1	-1	-4	2	1	-1	2	-3	-3	3	-2	-3	-3	-1	0	-5	-3	-1	8	14	5	4	0	8	10	4	6	1	2	19	1	1	1	5	6	0	1	4	0.32	0.28
92 S	-2	4	2	-1	-3	1	-1	-3	-1	-1	-1	2	0	-2	-4	1	0	-5	-1	-1	2	24	8	4	0	5	3	1	1	4	6	14	3	2	1	10	5	0	3	4	0.36	0.31
93 H	-2	-5	-3	-5	-8	-6	-4	-1	11	-3	-3	-5	-4	-5	-8	-5	-3	-8	-1	-4	3	0	2	1	0	0	1	5	75	2	3	1	0	0	0	1	2	0	2	2	2.99	1.32
94 A	2	1	-2	-2	1	-1	-2	0	-2	1	1	-1	2	-1	0	-2	-1	-1	-2	-2	2	18	8	1	2	3	2	2	7	1	7	6	12	1	4	1	4	4	0	1	16	0.14	0.16
95 T	0	1	2	0	-3	1	-1	0	1	-2	-2	1	0	-2	0	1	1	0	0	-2	8	9	9	5	0	5	3	6	4	2	3	7	3	2	5	9	10	1	3	3	0.11	0.13
96 K	-2	4	-2	-4	-2	0	-2	-3	4	0	-2	2	-1	0	-4	-2	-3	-1	4	0	3	24	1	1	1	5	3	2	9	6	4	13	1	4	1	2	1	1	13	6	0.55	0.37
97 H	-2	0	0	-3	-5	-3	-3	3	5	-2	1	-1	-2	1	-2	-3	-3	0	3	-3	3	6	4	2	0	1	2	23	16	2	13	3	1	7	2	1	1	1	10	2	0.52	0.37
98 K	-1	1	2	-1	-1	1	1	-2	4	0	-1	-3	2	-1	-4	-2	0	-2	-3	-3	2	5	7	9	3	1	6	2	18	2	4	2	14	1	0	1	6	2	0	1	14	0.24	0.22
99 I	-2	-2	-4	-4	-3	-2	-2	-5	-4	4	1	-4	2	1	-4	-3	1	-2	-5	4	3	3	1	1	0	2	2	1	0	22	10	1	4	5	1	2	8	1	0	33	0.68	0.44
100 P	-2	1	1	3	-6	1	0	-1	1	-4	-4	3	-5	-4	3	-1	0	-2	-5	-4	3	7	6	15	0	5	6	5	3	1	2	19	0	1	16	4	7	1	0	1	0.47	0.38
101 I	1	0	-3	-1	-5	0	1	-3	0	0	-1	0	0	-3	5	-1	-1	-2	-5	0	12	4	1	4	0	4	9	2	5	6	7	2	1	28	4	3	1	0	6	0.46	0.36
102 K	-1	0	-1	2	-2	3	4	-2	3	-4	-4	1	-2	-3	-2	1	-1	-6	-2	-1	4	4	2	12	1	11	23	3	6	1	2	9	1	1	2	9	4	0	1	4	0.43	0.36
103 Y	-3	-4	1	0	-6	0	-4	-4	7	-2	-2	-4	2	2	-6	-2	-3	-3	6	-3	2	1	8	5	0	5	1	1	23	2	5	1	5	8	0	3	1	0	27	2	1.09	0.64
104 L	-2	-4	-1	-4	-4	-1	-2	-2	1	0	0	-1	1	5	-1	-1	-1	4	3	0	4	0	3	0	0	3	3	3	3	5	7	5	4	28	4	4	4	5	9	5	0.47	0.33
105 E	-1	0	1	2	-4	1	1	-1	1	-1	-2	1	0	-1	1	0	-1	-1	3	0	-1	5	4	6	14	0	6	11	3	4	3	3	11	3	3	6	4	4	3	4	0.16	0.17
106 F	0	-1	-1	0	0	0	0	-1	0	0	1	-1	1	0	0	-1	0	2	0	1	11	3	3	4	2	3	6	3	3	7	14	4	4	5	4	4	5	3	4	10	0.02	0.04
107 I	-2	-3	-5	-3	-5	-3	-4	-3	-5	2	3	-3	5	4	-3	-5	-2	-4	0	2	4	1	0	2	0	1	1	2	0	11	24	1	12	17	1	0	2	0	3	16	0.68	0.42
108 S	0	1	-1	-2	-1	0	1	3	-1	-2	-2	1	0	0	-3	0	-1	2	-1	-1	8	8	3	1	3	8	24	1	3	4	9	2	4	1	6	4	3	2	5	0.20	0.19
109 E	-1	-1	1	1	-2	1	3	0	2	-3	-3	1	-2	-3	1	0	0	-5	-2	0	5	3	6	9	1	7	18	8	5	1	2	8	1	1	7	5	5	0	1	6	0.22	0.22
110 A	3	-3	-1	-3	5	-1	-2	-3	-1	1	-1	-4	-1	-2	0	1	0	-1	-2	2	25	1	3	1	11	3	2	2	2	7	5	0	2	1	4	9	6	1	1	13	0.38	0.33
111 I	-4	-8	-8	-9	0	-8	-8	-9	-8	4	4	-8	4	5	-8	-7	-3	1	-1	1	2	0	0	0	2	0	0	0	0	17	34	0	8	22	0	0	2	2	2	8	1.27	0.65
112 I	-2	-1	-2	-2	-1	-1	-2	-4	0	3	3	2	2	1	-2	-4	-1	-2	-2	2	3	3	2	2	1	3	3	1	3	15	30	3	5	2	2	1	4	1	1	14	0.44	0.29
113 H	1	-1	-1	1	-1	2	3	-2	1	-3	-3	1	-3	-1	-3	0	0	2	1	-1	10	4	3	9	2	8	17	3	3	1	3	10	1	3	1	6	5	3	5	4	0.21	0.21
114 V	2	-4	-4	-6	-2	-5	-3	-2	-1	-1	-1	-3	2	2	-5	0	3	-1	2	3	18	1	1	0	1	0	2	3	1	3	5	1	4	7	0	5	18	1	8	21	0.49	0.37
115 L	-2	-7	-7	-7	-1	-6	-7	-7	-7	4	4	-7	4	2	-7	-3	-4	3	-3	2	4	0	0	0	1	0	0	0	0	17	40	0	9	7	0	2	1	4	1	12	1.07	0.55
116 H	2	2	-1	-1	0	2	2	-1	-1	-2	-3	3	0	-5	-2	0	-1	-2	-2	-2	17	9	2	3	2	8	12	4	2	2	2	17	3	0	2	6	4	1	1	3	0.26	0.24
117 S	-1	0	-1	2	-2	2	4	-3	1	-2	-3	0	-2	-3	-2	2	0	-2	-2	-3	6	5	3	10	1	8	26	2	3	2	3	5	1	1	2	12	5	1	2	2	0.37	0.31
118 R	0	1	0	-2	0	0	0	-1	3	0	-1	1	-1	-1	-3	0	1	1	0	1	10	7	4	1	2	3	5	5	7	5	7	9	1	2	0	5	9	2	3	12	0.09	0.12

119	H	-1	-3	-2	-3	0	-2	-2	-2	-2	0	5	-4	1	2	-4	-2	-2	-5	0	0	4	1	2	1	2	2	3	4	1	4	48	1	3	9	1	3	2	0	3	6	0.64	0.38
120	P	-1	-2	-2	-1	-1	0	-1	5	-2	-3	-5	2	-2	-5	2	-2	-2	-5	-3	-4	5	2	2	4	1	4	5	42	1	1	1	11	1	0	10	3	3	0	1	1	0.73	0.44
121	G	-1	0	1	3	2	0	2	0	0	-2	-3	0	-2	-5	0	0	0	-5	-4	-2	6	4	7	18	5	4	13	7	2	2	3	6	1	0	5	8	5	0	0	3	0.28	0.23
122	D	0	1	1	2	0	2	2	-1	1	-2	-2	1	0	-2	-3	0	-1	-4	-2	-2	8	9	7	13	2	8	13	4	4	2	4	8	2	2	1	7	3	0	2	3	0.19	0.18
123	F	-2	-2	-4	-5	-1	-4	-3	-5	0	0	1	-3	2	6	-5	-3	-5	8	3	-1	4	2	1	1	2	1	2	1	2	5	11	2	5	28	1	3	1	18	8	4	1.11	0.57
124	G	-2	-3	2	3	-1	-5	-2	0	-6	-4	-6	-1	-6	-7	-3	2	6	-7	-6	-4	3	2	10	13	1	0	2	6	0	1	0	3	0	0	2	15	40	0	0	1	0.99	0.64
125	A	1	-1	-1	3	-4	0	2	0	-1	-4	-3	0	-1	-4	4	0	-1	-2	-2	-3	12	3	3	14	0	4	12	6	2	1	3	5	2	1	21	6	3	1	1	1	0.40	0.33
126	D	0	-2	1	3	-4	0	5	-4	-2	-4	-4	0	-6	-2	-2	-1	-1	-7	-3	-4	7	2	5	17	0	5	38	1	1	1	2	4	0	2	2	4	4	0	1	1	0.77	0.53
127	A	1	-3	-2	-1	0	-1	-1	-3	-1	2	1	-4	2	-2	-3	-2	2	-2	-2	3	12	1	2	4	2	3	4	2	1	10	10	1	6	1	2	2	14	1	1	21	0.31	0.26
128	Q	1	2	-1	0	0	2	3	-2	1	-1	-2	2	-1	-4	-3	-2	-1	-5	-2	-1	12	9	2	5	2	9	19	2	4	3	5	13	2	1	1	2	3	0	1	5	0.29	0.26
129	G	2	-1	1	2	0	2	2	-1	0	-3	-2	1	-1	-4	-4	1	-1	-5	-5	-2	15	3	5	11	2	8	16	4	2	2	5	8	2	1	0	9	4	0	0	3	0.26	0.25
130	A	6	-7	-4	-5	-2	-4	-6	-2	-7	0	-4	-7	-3	-8	-7	1	-1	-3	-7	-1	69	0	1	1	1	1	0	3	0	4	2	0	1	0	0	9	3	0	0	4	1.56	0.89
131	M	-10	-7	-6	-12	-4	-10	-11	-11	-10	-5	-4	-11	1	-1	-12	-7	-7	13	1	-6	0	0	1	0	1	0	0	0	0	1	2	0	3	2	0	0	1	84	5	1	4.56	1.85
132	N	-1	2	1	2	-2	0	1	-1	0	-1	-3	2	-2	-3	-4	1	2	-2	-3	-2	5	11	7	10	1	3	10	4	2	4	2	14	1	1	0	9	12	1	0	3	0.26	0.24
133	K	0	0	-1	0	-2	1	0	-4	1	-2	-3	5	-1	-6	-4	0	0	-1	-4	-2	8	5	3	6	1	5	6	1	3	3	3	36	2	0	0	6	7	1	1	2	0.56	0.41
134	A	2	-7	-7	-7	3	-6	-5	-4	-7	0	3	-6	0	4	-7	-4	1	-6	-1	2	17	0	0	0	5	0	0	1	0	4	27	0	2	17	0	1	7	0	2	15	0.80	0.49
135	L	-4	-8	-8	-9	-3	-7	-8	-4	-6	2	3	-8	2	5	-8	-7	-4	3	6	-1	1	0	0	0	1	0	0	2	0	12	26	0	5	22	0	0	1	4	23	5	1.32	0.67
136	E	0	1	1	3	-2	1	0	1	-1	-3	-3	1	-4	-3	-4	1	1	0	-2	-3	9	8	6	18	1	7	6	12	1	1	3	7	0	1	0	8	7	1	1	1	0.28	0.25
137	L	0	-3	-1	-3	-2	-2	-2	-2	1	1	0	-3	-1	4	-5	-2	0	-2	3	2	8	2	4	1	1	2	3	3	3	7	9	2	1	19	0	3	6	0	10	17	0.37	0.27
138	F	-2	-8	-8	-8	-1	-5	-8	-8	-3	6	2	-7	3	2	-8	-5	-4	-2	-6	4	4	0	0	0	1	1	0	0	1	36	19	0	7	8	0	1	1	1	0	22	1.23	0.62
139	R	3	-3	-2	-3	1	-1	-3	-1	-2	2	0	-2	1	1	-5	0	0	0	1	1	25	1	2	1	3	2	1	5	1	14	7	2	4	6	0	6	7	1	4	7	0.28	0.24
140	K	1	-1	2	2	-1	0	2	-1	1	-4	-4	1	-2	-2	-3	1	1	-1	-1	-2	14	3	9	13	1	5	13	5	3	1	1	7	1	2	1	11	7	1	3	2	0.27	0.25
141	D	0	-1	-1	-2	-1	0	0	-1	2	2	0	0	0	0	-2	-1	0	-4	-1	2	10	2	4	1	1	4	7	5	6	12	8	5	3	3	2	3	7	0	2	16	0.10	0.12
142	I	-5	-7	-8	-8	-3	-5	-8	-8	-8	5	3	-7	8	-1	-8	-6	-4	-7	-3	1	1	0	0	0	1	1	0	0	0	27	27	0	30	3	0	0	1	0	1	9	1.63	0.78
143	A	0	0	-1	0	-4	0	0	-4	-2	2	-1	2	1	1	-4	0	1	-5	-3	0	7	6	3	4	0	3	6	1	1	14	6	14	3	7	0	9	7	0	0	7	0.18	0.19
144	A	2	0	0	1	-5	2	3	-2	0	-3	-3	2	-3	-6	-4	1	0	-6	-5	-2	17	5	5	6	0	7	22	3	2	2	2	12	0	0	0	9	6	0	0	2	0.39	0.33
145	K	2	-2	0	-4	-5	-1	0	5	1	-3	-4	1	-5	-3	-1	-1	-2	-6	-4	-3	18	2	4	0	0	2	8	38	3	2	2	9	0	1	3	4	2	0	0	2	0.63	0.42
146	Y	0	-4	-5	-5	-1	0	-1	-1	1	-1	0	-4	2	2	-6	-3	-4	1	7	-3	8	1	0	0	1	5	5	5	2	3	10	1	5	8	0	2	0	2	41	1	0.98	0.57
147	K	0	3	0	3	-2	0	2	-4	-3	-4	-2	4	1	-5	-3	0	-2	-6	-5	-3	10	15	4	14	1	4	13	0	0	1	4	23	4	0	1	5	2	0	0	1	0.48	0.36
148	E	-3	0	-1	3	-7	3	5	-5	-2	-6	-6	2	-5	-7	-3	-1	-4	-7	-5	-4	1	5	2	15	0	14	42	0	1	0	0	14	0	0	1	4	0	0	0	1	1.07	0.61
149	L	4	-4	-2	-2	-1	-1	-1	-3	-2	1	2	-3	1	-2	-4	-1	0	-4	-2	0	36	0	2	1	1	2	4	0	1	8	22	0	4	1	0	3	6	0	1	7	0.38	0.31
150	G	-1	0	1	0	-4	-1	-2	6	-1	-2	-5	-1	-4	-5	-4	1	-3	-5	-2	-4	3	5	5	5	0	2	2	60	2	3	0	2	0	0	0	10	0	0	2	0	0.90	0.39
151	Y	2	-1	-3	-4	-3	-3	-1	-3	0	-3	-3	-3	-3	3	-4	-1	0	8	4	-2	24	4	0	0	0	0	3	0	2	0	0	0	0	14	0	3	8	21	18	2	0.80	0.33
152	Q	0	2	0	2	-4	5	3	-3	-2	-4	-4	0	0	-4	-3	-1	-2	-4	-3	-3	10	11	3	12	0	41	16	0	0	0	0	3	3	0	0	3	0	0	0	0	0.67	0.30
153	G	-1	-4	-2	-3	-4	-3	-4	7	-4	-5	-5	-3	-4	-5	-4	-2	-3	-4	-5	-5	0	0	0	0	0	0	0	100	0	0	0	0	0	0	0	0	0	0	0	0	1.72	0.31

	K	Lambda
Standard Ungapped	0.1344	0.3180
Standard Gapped	0.0427	0.2670
PSI Ungapped	0.1394	0.3176
PSI Gapped	0.0427	0.2670
