# Supplementary figures and images for: A simple strategy to enhance the speed of protein secondary structure prediction without sacrificing accuracy
Source: PLoS One. 2020 Jun 30;15(6):e0235153. doi: 10.1371/journal.pone.0235153 (PMC7326220; doi:10.1371/journal.pone.0235153)

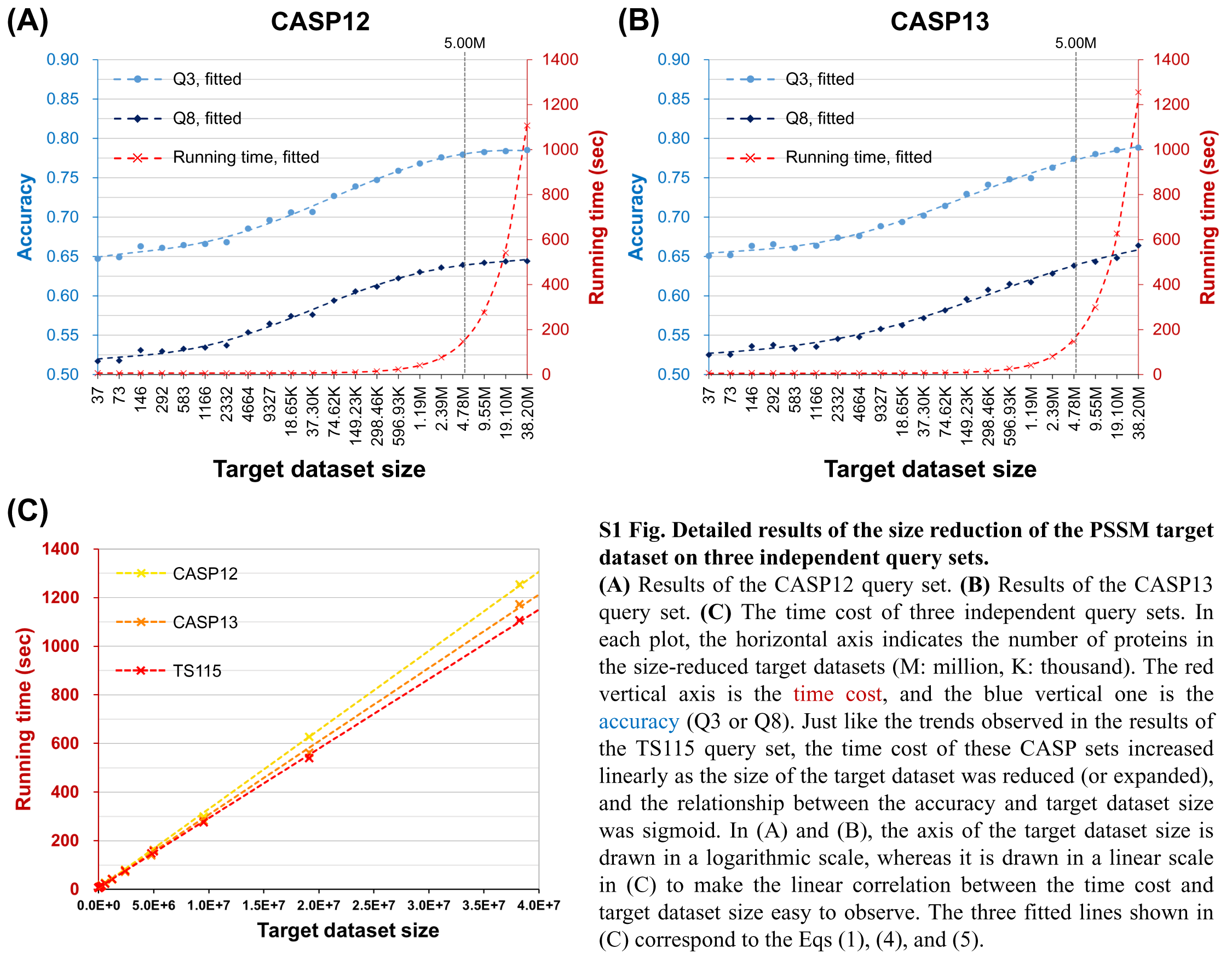

Supplement: S1 Fig — (TIF) [file pone.0235153.s003.tif]

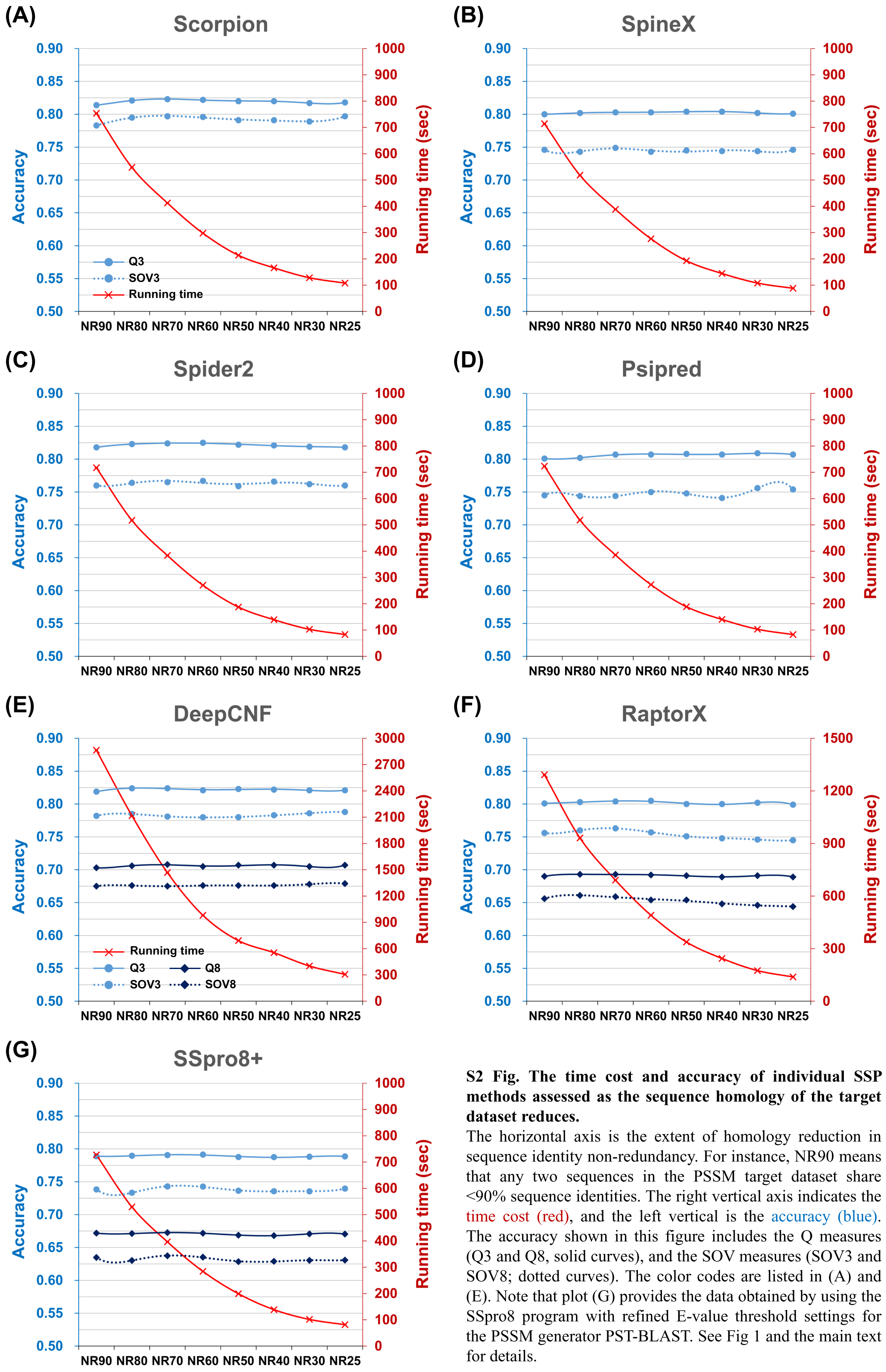

Supplement: S2 Fig — (TIF) [file pone.0235153.s004.tif]

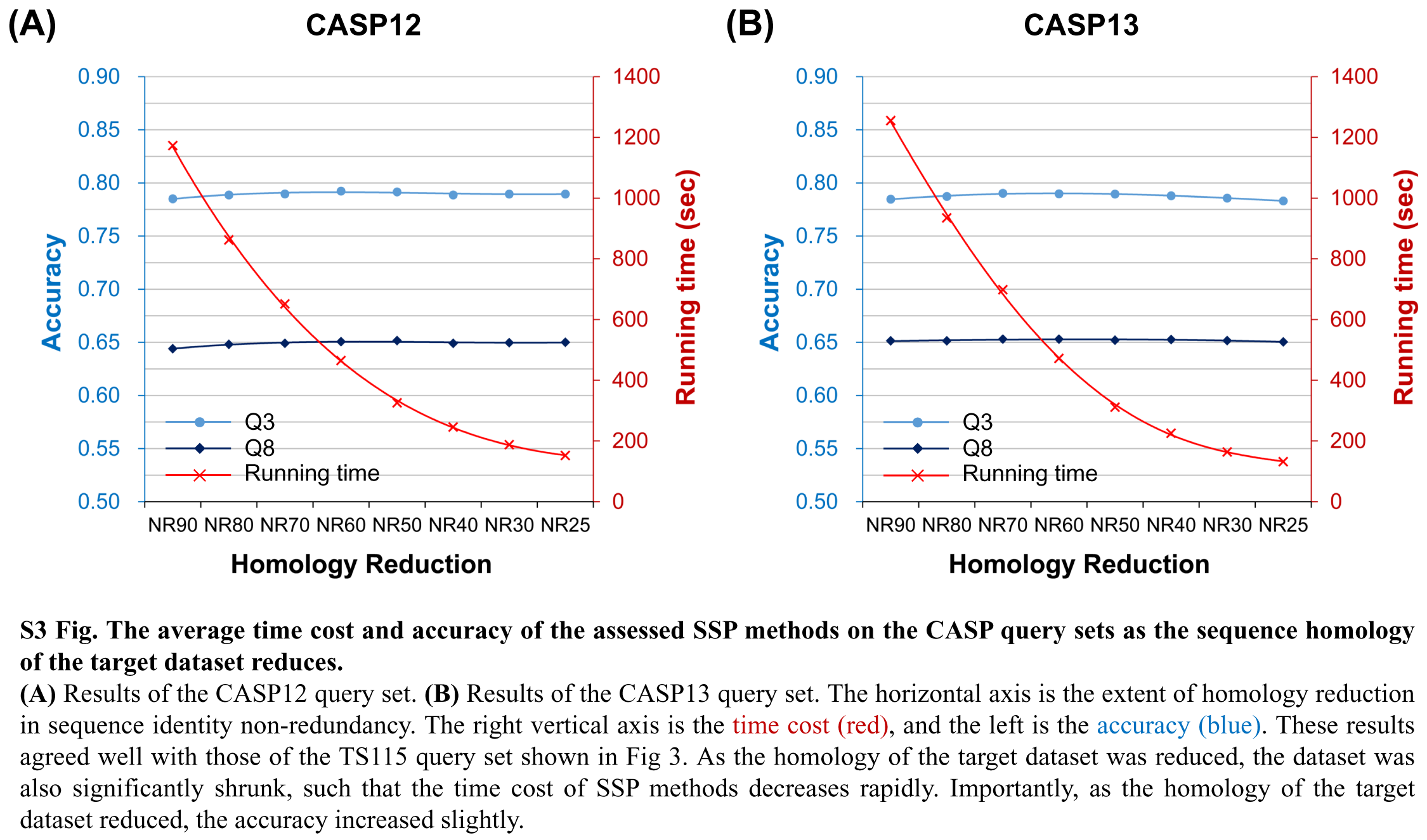

Supplement: S3 Fig — (TIF) [file pone.0235153.s005.tif]

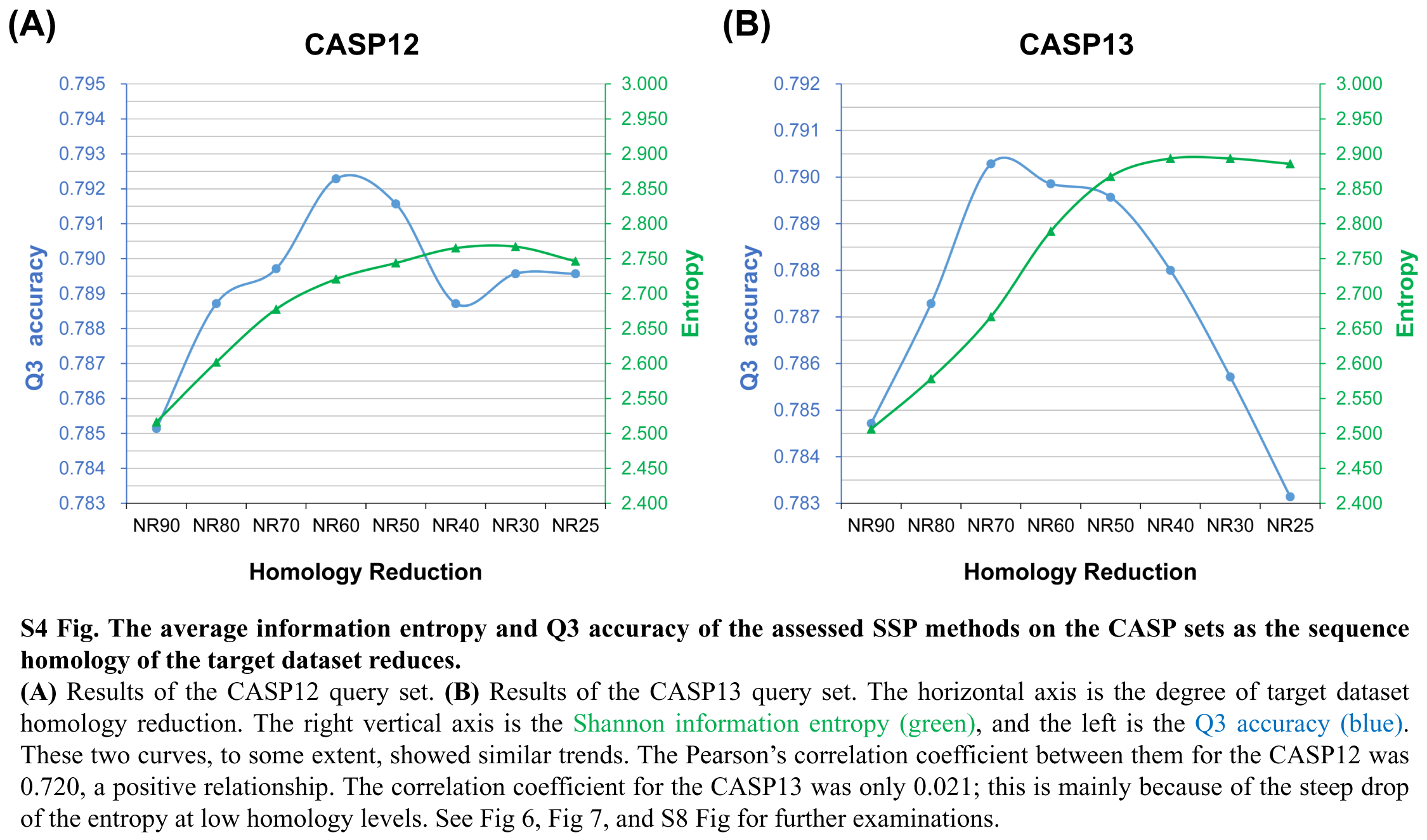

Supplement: S4 Fig — (TIF) [file pone.0235153.s006.tif]

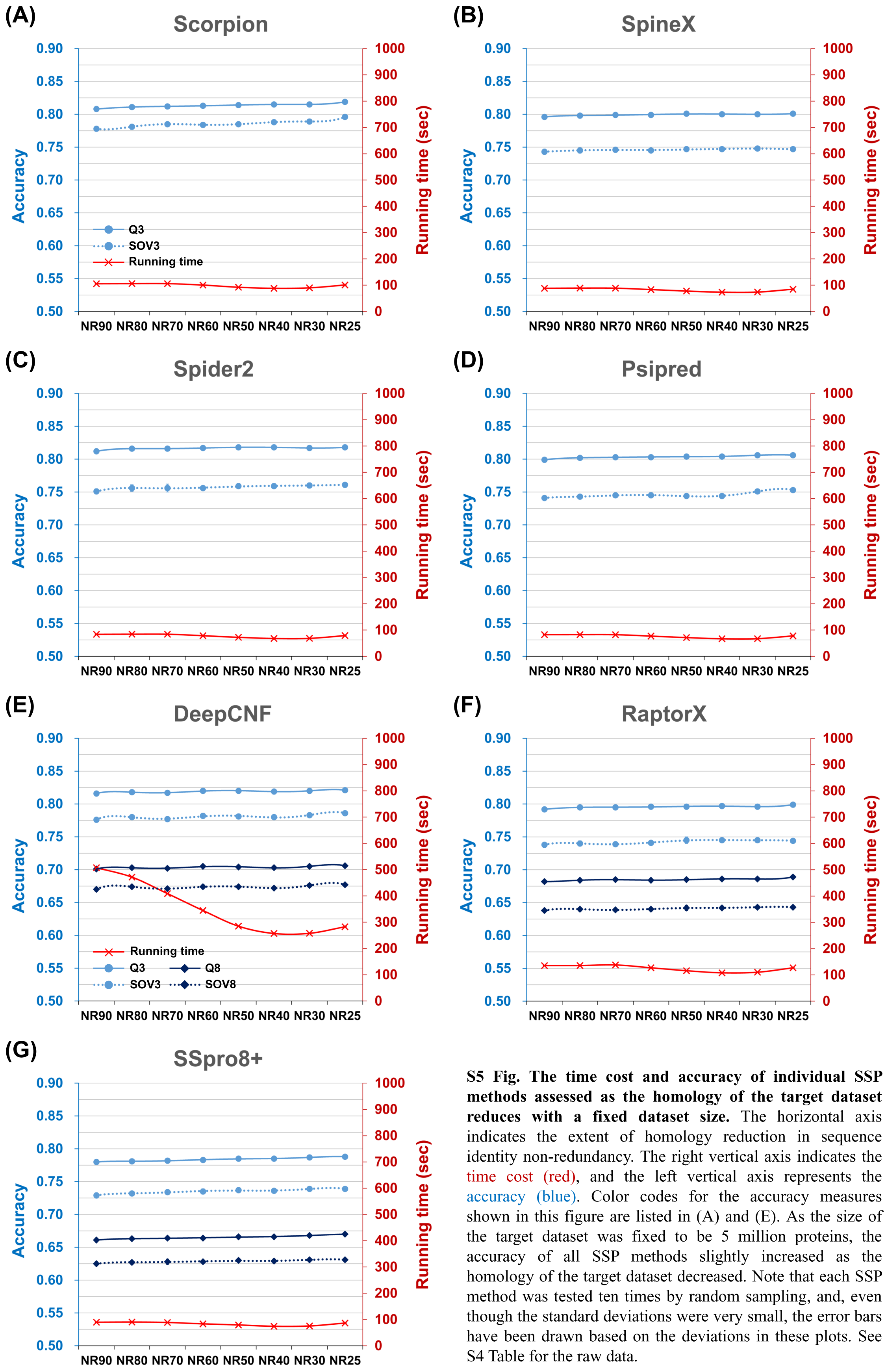

Supplement: S5 Fig — (TIF) [file pone.0235153.s007.tif]

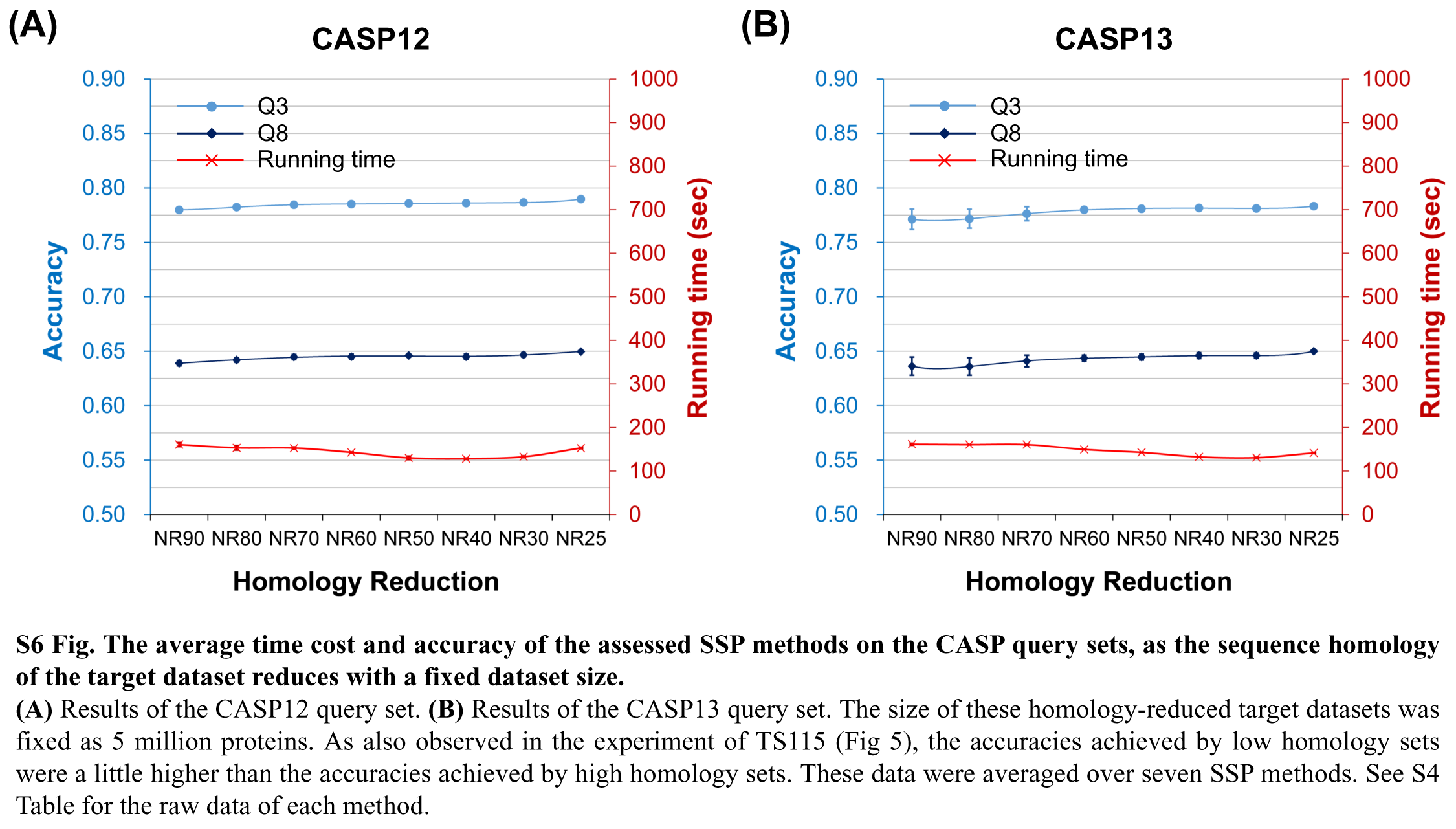

Supplement: S6 Fig — (TIF) [file pone.0235153.s008.tif]

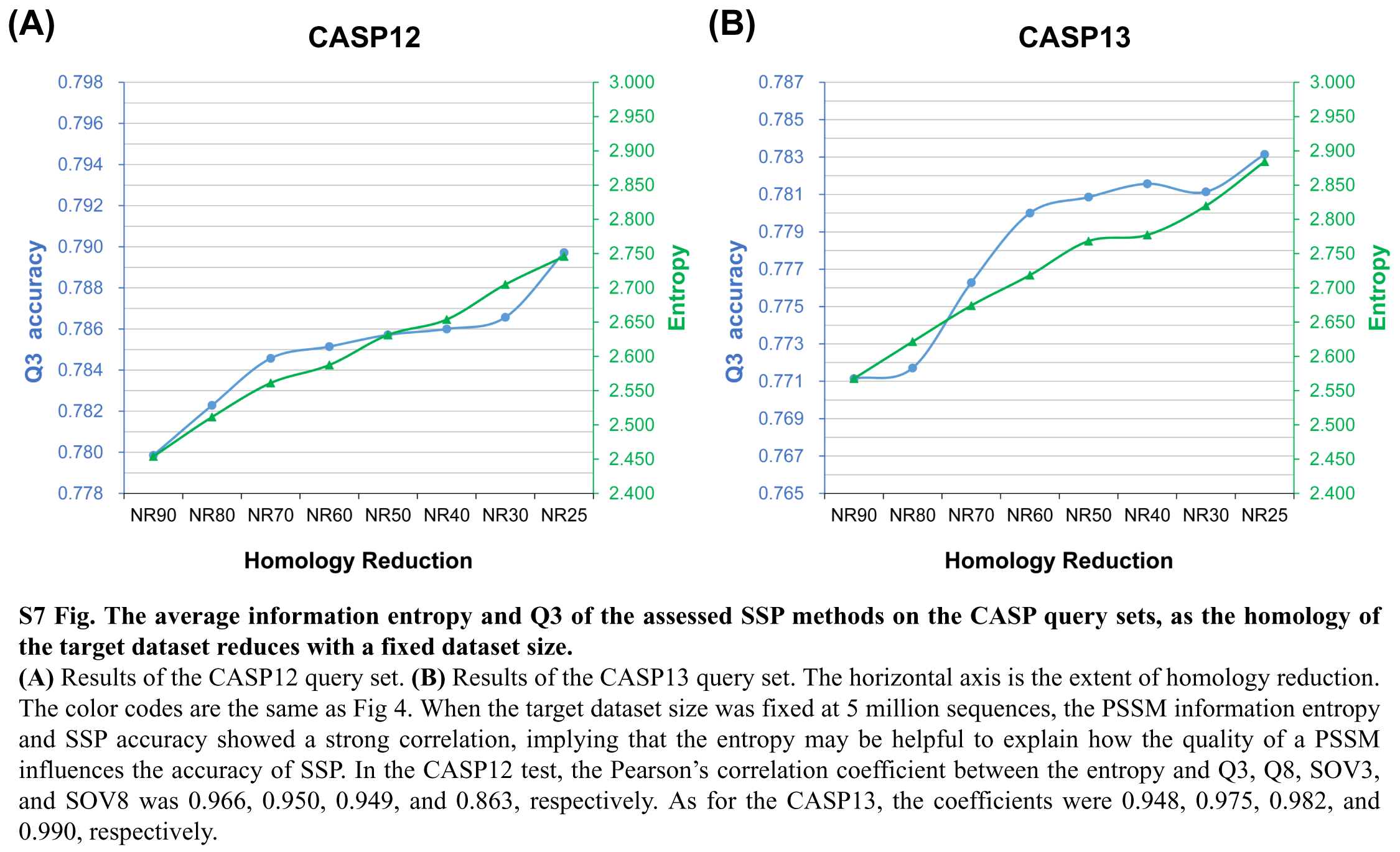

Supplement: S7 Fig — (TIF) [file pone.0235153.s009.tif]

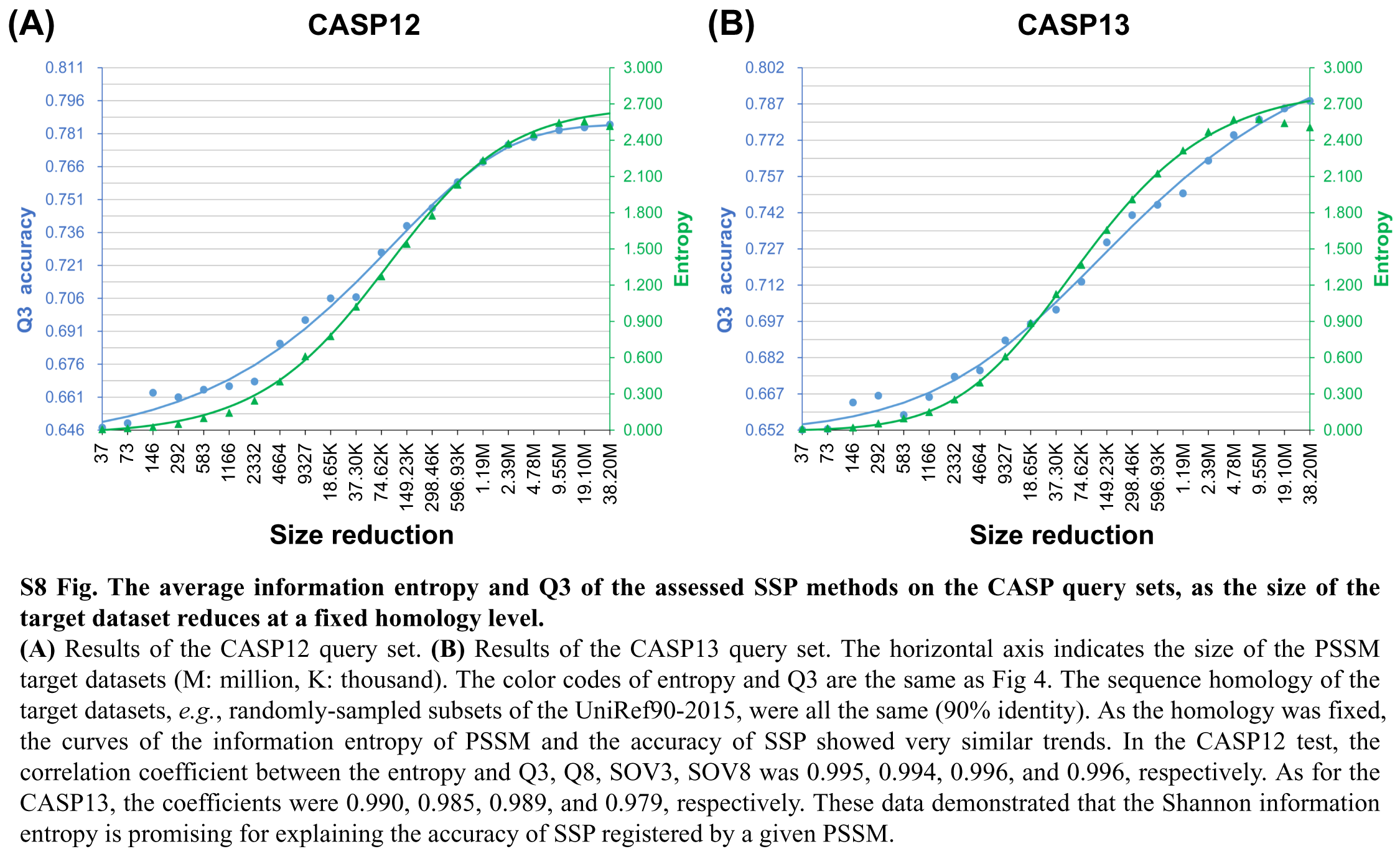

Supplement: S8 Fig — (TIF) [file pone.0235153.s010.tif]
